# The novel anti-phage system Shield co-opts an RmuC domain to mediate phage defense across *Pseudomonas* species

**DOI:** 10.1371/journal.pgen.1010784

**Published:** 2023-06-05

**Authors:** Elliot Macdonald, Rosanna Wright, James P. R. Connolly, Henrik Strahl, Michael Brockhurst, Stineke van Houte, Tim R. Blower, Tracy Palmer, Giuseppina Mariano

**Affiliations:** 1 Microbes in Health and Disease Theme, Newcastle University Biosciences Institute, Newcastle University, Newcastle upon Tyne, United Kingdom; 2 Division of Evolution, Infection and Genomics, School of Biological Sciences, University of Manchester, Manchester, United Kingdom; 3 Centre for Bacterial Cell Biology, Biosciences Institute, Faculty of Medical Sciences, Newcastle University, Newcastle upon Tyne, United Kingdom; 4 Environment and Sustainability Institute, University of Exeter, Penryn Campus, Penryn, Cornwall, United Kingdom; 5 Department of Biosciences, Durham University, Stockton Road, Durham, United Kingdom; Institut Cochin, FRANCE

## Abstract

Competitive bacteria-bacteriophage interactions have resulted in the evolution of a plethora of bacterial defense systems preventing phage propagation. In recent years, computational and bioinformatic approaches have underpinned the discovery of numerous novel bacterial defense systems. Anti-phage systems are frequently encoded together in genomic loci termed defense islands. Here we report the identification and characterisation of a novel anti-phage system, that we have termed Shield, which forms part of the *Pseudomonas* defensive arsenal. The Shield system comprises the core component ShdA, a membrane-bound protein harboring an RmuC domain. Heterologous production of ShdA alone is sufficient to mediate bacterial immunity against several phages. We demonstrate that Shield and ShdA confer population-level immunity and that they can also decrease transformation efficiency. We further show that ShdA homologues can degrade DNA *in vitro* and, when expressed in a heterologous host, can alter the organisation of the host chromosomal DNA. Use of comparative genomic approaches identified how Shield can be divided into four subtypes, three of which contain additional components that in some cases can negatively affect the activity of ShdA and/or provide additional lines of phage defense. Collectively, our results identify a new player within the *Pseudomonas* bacterial immunity arsenal that displays a novel mechanism of protection, and reveals a role for RmuC domains in phage defense.

## Author summary

The evolutionary pressure exerted by bacteriophages has driven bacteria to acquire numerous defense systems. Recent studies have highlighted the extraordinary diversity of these systems, uncovering exciting links between bacterial and eukaryotic immunity. Here we describe a novel anti-phage system, named Shield, found within *Pseudomonas* species. We identify several Shield subtypes, all harboring the same core component, and describe its mode of action. The growing instance of multidrug-resistant bacterial infections urgently requires the development of alternative treatments. Phage therapy is a particularly pertinent approach to treat multi-drug resistant *Pseudomonas aeruginosa* strains causing severe lung infection in cystic fibrosis patients. A detailed understanding of bacterial immunity and phage counter-strategies is an essential step to underpin the rational design of phage therapy to fight disease.

## Introduction

In response to continuous predation from bacteriophages (phages), bacteria have evolved numerous defense systems that, collectively, can be considered to provide ‘bacterial immunity’ [1–4]. Historically, the best described phage defense systems are the restriction-modification (RM) systems, which represent an example of bacterial innate immunity. Here the modification unit covalently modifies host DNA whilst the restriction component recognises specific sequence patterns on unmodified invading DNA to mediate its degradation [5]. The more recently discovered DISARM (defence island system associated with restriction–modification) and BREX (Bacteriophage Exclusion) system also adopt a step of DNA methylation to discriminate between self and non-self DNA, however their still uncharacterised mechanism of phage inhibition appears to be more complex [2,6,7]. In particular, the DISARM system is often found next to RM systems and can be found in 2 subtypes, all sharing the *drmABC* genes, which exhibit helicase (DrmA), a DUF1998 (DrmB) and a phospholipase D (DrmC) predicted domains. DrmAB were recently shown to recognise the 5’-end of single stranded unmodified phage DNA [6] but the exact mechanism of phage inhibition and the role of other DISARM components remain unknown [7] In contrast, the CRISPR-Cas systems represent the first example of bacterial adaptive immunity wherein guide RNAs, specific for invading phage DNA sequences, direct effector nucleases to foreign nucleic acids [8]. Understanding the mechanism of action of these systems has underpinned their utility as tools that revolutionised the field of gene editing. RM and CRISPR-Cas systems are usually considered part of the first line of defense, as their defense strategy is to swiftly remove invading phage DNA [9]. Conversely, other anti-phage systems adopt a strategy broadly defined as abortive infection (Abi), wherein infected cells undergo cell death to prevent release of mature phages within the population [9,10]. Abortive infection systems are considered part of a second line of defense, although the division between ‘first’ and ‘second’ lines of defense was recently shown to be less defined, with some instances of CRISPR-Cas subtypes also inducing bacterial stasis or death [11,12].

A defining trait of known anti-phage systems is that they are frequently encoded together at genomic hotspots, known as ‘defense islands’ [11]. This concept has been exploited in recent years to identify novel defense loci encoded close to known systems, revealing numerous additional anti-phage systems that were never described before [13–18]. This has uncovered further innovative defensive strategies including NAD^+^ depletion, mediated by the Thoeris and Defense-associated sirtuin (DSR) systems [19,20] and depletion of the cellular dNTP pool [21,22]. Depletion of cellular dNTPs is a conserved antiviral strategy also found in eukaryotes, demonstrating a clear link between prokaryotic and eukaryotic immunity [23]. Indeed, a striking characteristic that has emerged from recent studies is the evolutionary relatedness between many anti-phage systems and innate immune mechanisms in plants and animals [15,16,21,24–28]. Many of these new systems contain genes encoding related protein domains, indicating a shared distribution of biochemical activities in phage defense [29]. Additional examples of bacterial anti-phage defense systems that presumably are ancestors of eukaryotic immunity are represented by the viperins, gasdermins, NLR-related anti-phage systems, Toll/IL-1 receptor (TIR) domains (carried by the Thoeris system) and the bacterial cyclic oligonucleotide-based signalling system (CBASS) [19,21,25,26,28].

However, despite these recent discoveries, the defense mechanisms adopted by the majority of the newly-identified anti-phage systems remain unknown. Furthermore, these recent approaches have highlighted that many more genes involved in bacterial immunity still await discovery.

In this study we report the identification of a previously uncharacterised phage defense system that we have termed Shield. The system, which is encoded across *Pseudomonas* species, has an RmuC domain-containing protein, ShdA, as its active component. RmuC-domain containing proteins were first associated with regulation of the inversion rate of DNA short inverted repeats in *E*. *coli*, albeit their role in the process was not clearly defined [30]. Sequence similarity searches further revealed very weak homology between RmuC and the family of structural maintenance of chromosome proteins (SMC) [30]. *E*. *coli* RmuC contains a predicted N-terminal trans-membrane helix, a central α/β domain and a disordered C-terminus, placing the *E*. *coli* RmuC-domain containing protein within the PD-(D/E)XK nuclease superfamily [31]. Nevertheless, a direct role for RmuC-domain containing proteins in providing defense against phages and mobile genetic elements was not previously reported.

ShdA exhibits DNA-degrading activity *in vitro*, and expression *in vivo* leads to a remodelling of the host nucleoid structure. While ShdA alone is sufficient to confer phage defense, Shield occurs as four distinct subtypes, with additional components found in three of the subtypes. We demonstrate that additional components of two subtypes negatively affect ShdA activity or provide additional phage defense mechanisms. Collectively, our study uncovers the previously uncharacterised role of RmuC domains in bacterial immunity and describes the RmuC-mediated mechanism of phage inhibition.

## Results

### Genetic identification of a candidate novel anti-phage system

Previous studies have demonstrated that bacterial defense systems cluster together in chromosomal hotspots, defined as ‘defense islands’ and that their mobilisation is dependent on mobile genetic elements [17,32,33]. Genetic neighborhood analysis of known anti-phage systems has allowed the systematic discovery of many novel defense systems [2,4,13,14,16,17,32]. Following the same principle, we aimed to identify bacterial operons, situated in the context of defense islands, whose role has not yet been associated with defense against invasion of foreign DNA.

**Fig 1:**
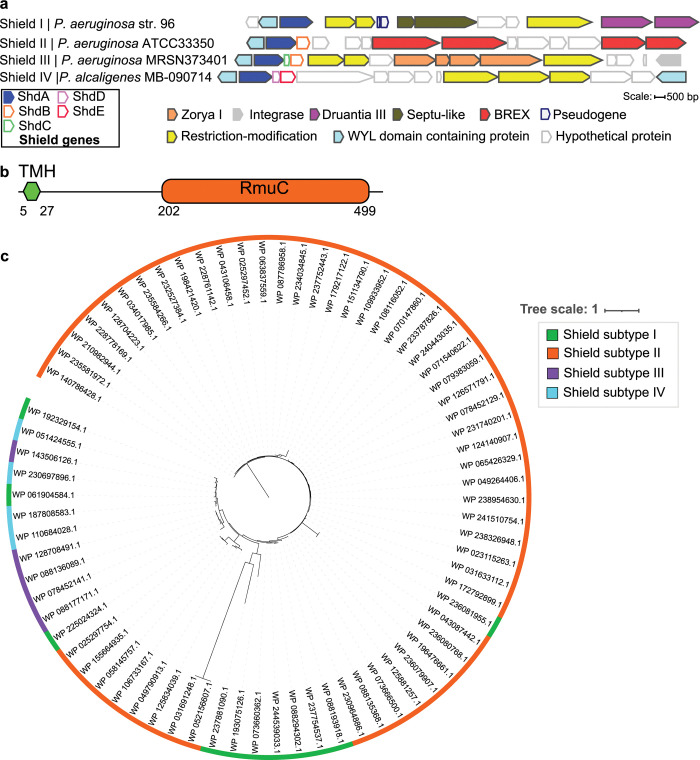
Identification of a novel anti-phage system. **(a) Schematic of the genomic neighbourhood of Shield systems in representative genomes**. Known defense genes were predicted using PFAM and Defense-finder [34]. ShdA is in blue and other Shield partners are represented with coloured outline, as indicated. The full set of Shield subtypes is shown in S2 Fig and known anti-phage systems annotations are reported in S6 Table. (b) Schematic representation of the predicted domain organisation of the candidate defence protein, ShdA. (c) Phylogenetic tree based on ShdA homologues. Coloured blocks indicate ShdA homologues belonging to each Shield subtype.

*Pseudomonas aeruginosa* is among the species that have been reported to encode many distinct anti-phage systems [34]. To identify new candidate anti-phage systems in this organism and across the genus we downloaded all available genome sequences of *Pseudomonas* species from the Refseq database to the ‘Scaffold’ assembly level (ftp://ftp.ncbi.nlm.nih.gov/genomes/ASSEMBLY_REPORTS/assembly_summary_refseq.txt, as of 11th June 2022/~3500 genomes), including entries that represent only portion of genomes (Scaffolds) that have been reconstructed from the assembly of whole-genome shotgun (WGS) sequencing data. Subsequently we used the Defense Finder tool to identify known anti-phage systems [34] (S1 Table). We then manually screened for flanking genes or operons that associated with the defense systems identified in S1 Table and whose function was either unknown (genes annotated to encode hypothetical proteins), associated with interaction/degradation of nucleic acids, or that could mediate cell death or stasis (i.e. peptidoglycan hydrolase, lipases, pore-formation, NAD^+^ depletion, etc.). Our search consistently revealed a gene that was encoded next to several known anti-phage systems (Fig 1A). We performed function predictions using the protein encoded by *P*. *aeruginosa* NCTC 11442/ATCC 33350 (assembly ID: GCF_001420205.1, locus tag: AN400_RS26690, protein ID: WP_023115263). This protein represents one of the most frequently occurring homologues of the new candidate defence gene we identified (S2 Table). Predictions with hmmscan against a PFAM database (26) revealed that the C-terminal region of AN400_RS26690 contains a PF02646 domain, typical of RmuC proteins (Fig 1B). Additionally, AN400_RS26690 harbors a predicted trans-membrane domain (TMH) at its N-terminus (Fig 1B).

Subsequently, to extend the search and detect a wider diversity of homologues, we built a Hidden Markov Model (HMM) model, including RmuC-like proteins from several species. We then queried a local Refseq of all bacterial genomes to the ‘contig’ assembly level (ftp://ftp.ncbi.nlm.nih.gov/genomes/ASSEMBLY_REPORTS/assembly_summary_refseq.txt, as of 11th June 2022/~150000 genomes). With the inclusion of contigs, which cover a smaller percentage of the full genome than Scaffolds, we aimed to expand the breadth of bacterial species considered in our search, to find more instances of RmuC-like domains associated with defense. The dataset was manually curated to include only those homologues found within defense islands (thus excluding housekeeping RmuC) (Figs 1A and S1). This resulted in ~70 unique protein identifiers and we renamed these protein homologues as ShdA. We subsequently used flanking gene (FlaGs) analysis [35] to define the genomic neighborhood of the *shdA* genes (Figs 1A and S1 and S3 and S4 Tables). From this we noted that although in some instances *shdA* could be found as an orphan gene, in other instances it co-occurred with subsets of additional genes which were always encoded downstream (Figs 1A and S1 and S3 Table). We renamed *shdA-*containing operons ‘Shield’ and classified them in 4 subtypes according to their gene composition. (Fig 1A and Table 1).

**Table 1:** Summary of predicted protein components for each Shield subtype.

Shield subtype	System components
Shield I	ShdA I
Shield II	ShdA II, ShdB II
Shield III	ShdA III, ShdB III, ShdC
Shield IV	ShdA IV, ShdD, ShdE

Despite the relaxed parameters used in search of RmuC homologues, Shield subtypes were, to date, only found in *Pseudomonas spp* and predominantly in *P*. *aeruginosa* (S2 Table). In particular, of ~14000 *Pseudomonas* sequences available to contig level in the Refseq database analysed, Shield subtypes were found in 6.8% of *Pseudomonas* genomes.

Where ShdA was encoded alone, we designated this Shield I. Of the other three candidate systems, Shield subtype II is the most widely distributed, representing 72% of the total Shield systems found and we renamed its partner gene *shdB*. Shield I, III and IV instead represented 15.1%, 6.8% and 5.5% of the total Shield systems found, respectively (Figs 1 and S1 and S2 Table). We named the other partner genes *shdC—shdE* according to the order they appeared in the FlaGs schematic representation of Shield subsystems (Fig 1A, Table 1). A BLAST search with strict query coverage and sequence similarity parameters (80–100% and 70–100%, respectively) revealed that ShdC, ShdD and ShdE proteins are predominantly found in association with Shield subsystems.

The 15 proteins encoded directly upstream and downstream of *shdA* were annotated using Defense-finder and PFAM predictions (Figs 1A and S1 and S4–S6 Tables), confirming that Shield subtypes localize adjacent to known anti-phage systems (Figs 1A and S1, and S4–S6 Tables). They also often associate with WYL-domain containing proteins, transcriptional regulators that are enriched in phage defense islands [3,36,37]. Furthermore, the genome neighborhood also encodes several viral proteins and integrases, in agreement with recent reports that mobile genetic elements (MGE) represent primary carriers of defense islands [17,32,33]. These findings further support the involvement of the Shield systems in bacterial immunity.

Alignment of ShdA homologues reveals a high degree of shared sequence identity, particularly towards the C-terminus (S2 and S3 Figs). A phylogenetic tree (based on the alignment in S3 Fig), reveals that although Shield subtype III is more similar to Shield subtype II in terms of gene composition (both having a ShdA and ShdB homologue), ShdA III homologues cluster closer to homologues from subtype I and IV (Fig 1C), whilst some ShdA I proteins are found in-between ShdA II branches (Fig 1C).

Within our ShdA homologues hits, we also found ~70 homologues embedded within DISARM-like operons (S4A and S5 Figs and S7 Table). DISARM-associated ShdA proteins exhibit a longer sequence length, with an additional sequence stretch at the N-terminus (S6 Fig). Phylogenetic analysis shows that DISARM-related ShdA homologues cluster separately from those of Shield, suggesting ShdA homologues associated to Shield and DISARM have diverged early (S4B Fig). Interestingly, the taxonomic distribution of DISARM-associated ShdA proteins is also exclusively limited to *Pseudomonas spp*. (S8 Table).

### ShdA is the core anti-phage defense module in Shield subtypes

**Fig 2:**
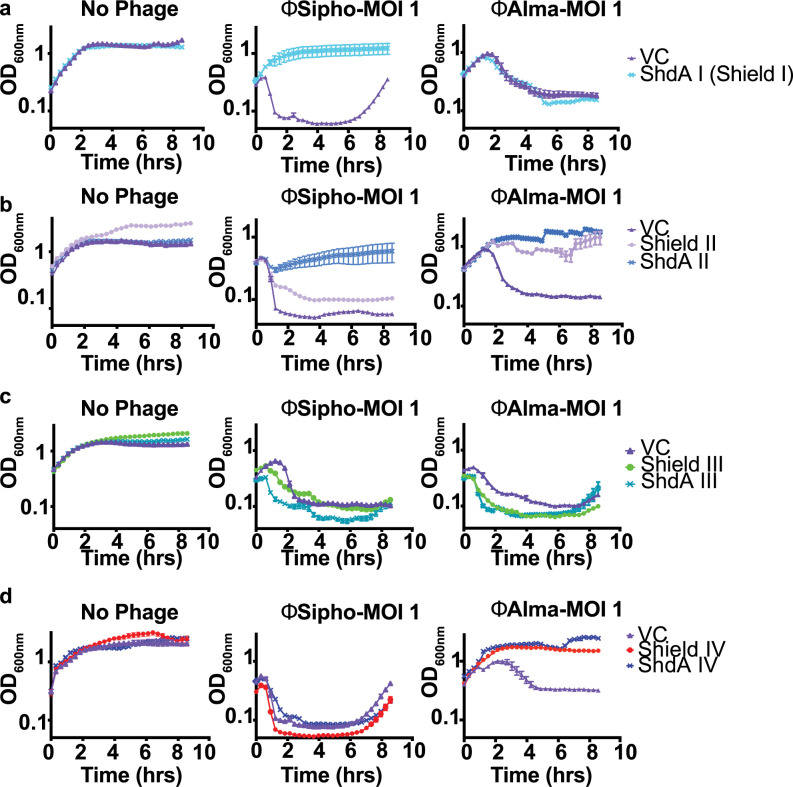
Shield I, Shield II and Shield IV prevent phage infection. Growth curves of *E*. *coli* MG1655 carrying empty pBAD18 (VC) or the same plasmid encoding **(a)** ShdA I (the sole component of the Shield I system), **(b)** the Shield II system or ShdA II only, **(c)** Shield III or ShdA III only, and **(d)** Shield IV or ShdA IV only. Strains were grown in LB medium supplemented with 0.2% L-arabinose and phages ϕSipho or ϕAlma were added at the start of the growth curve at a MOI = 1. Points show mean +/− SEM (n  =  3 biological replicates).

Since ShdA is the sole component of Shield I, we reasoned that this protein might represent the core ‘defense’ module of Shield systems. To investigate this, we cloned representative examples of *shdAI*—*shdAIV* under control of the arabinose-inducible promoter in plasmid pBAD18. We subsequently transformed *Escherichia coli* MG1655 with these constructs and tested their ability to protect *E*. *coli* growing in liquid culture from lysis by the unrelated lytic phages ϕSipho and ϕAlma (Fig 2).

We observed that Shield I, which only comprises the ShdA I module, confers protection against the phage ϕSipho (Fig 2A), but was ineffective against ϕAlma. Similarly, ShdA II and ShdA IV also provided defense against phage-mediated lysis (Fig 2B and 2D), with ShdA II protecting against the action of both ϕSipho and ϕAlma, and ShdA IV against ϕAlma only. We also tested the ability of the full Shield II and Shield IV systems to provide protection, and noted that in each case the cognate ShdA elicited at least similar levels of protection as the full system (Fig 2B and 2D). In addition, we also observed that for ϕSipho, ShdA II alone provided better protection compared to Shield II (Fig 2B). Conversely, ShdA III and Shield III were ineffective against both phages (Fig 2C). We conclude that at least three of the Shield subtypes are involved in bacterial immunity, and that the defense phenotype is dependent on the conserved component ShdA. We also conclude that the different subtypes we tested show phage-specific patterns of protection.

### Shield II and ShdA II mode of action involves reduction of phage burden

To better investigate the mode of action of Shield in phage defense *in vivo*, we focused on Shield II from *P*. *aeruginosa* NCTC 11442/ATCC 33350 (assembly ID: GCF_001420205.1), as this represents the most common Shield subtype (S2 Table).

**Fig 3:**
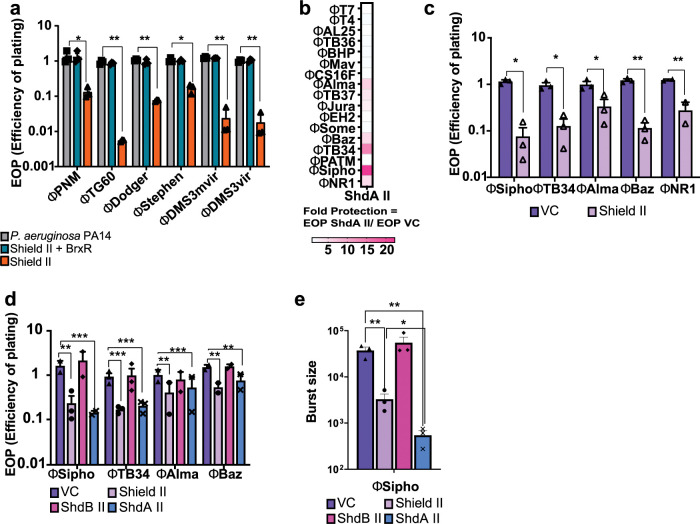
Characterisation of the anti-phage activity of Shield subtype II. **(a)** Efficiency of plating (EOP) measurement for *P*. *aeruginosa* PA14 csy3::LacZ (strain with a disrupted CRISPR-Cas system) carrying either a chromosomally-integrated Shield II with its native promoter, Shield II and the upstream WYL-domain containing protein or the empty transposon mini-Tn7 vector used for chromosomal integration. Strains were challenged with several phages as shown in panel a, Points show mean +/− SEM (n  =  3 biological replicates). (b) Evaluation of ShdA II fold protection against a suite of phages. Fold protection was obtained by dividing the value of the efficiency of plating (EOP) calculated for a strain expressing ShdA II by the EOP value of a strain carrying the empty vector, when infected with phages, as shown in panel b. (c) Efficiency of plating (EOP) measurement for *E*. *coli* MG1655 carrying empty vector (VC, pBAD18) or the same plasmid encoding Shield subtype II when infected with phages that showed susceptibility to ShdA II in Fig 2B. Points show mean +/− SEM (n  =  3 biological replicates). (d) Efficiency of plating (EOP) measurement for *E*. *coli* MG1655 carrying empty vector (VC, pBAD18) or the same plasmid encoding the Shield II system, ShdA II only or ShdB II only when challenged with phages ϕSipho, ϕTB34, ϕAlma, ϕBaz and ϕNR1. Points show mean +/− SEM (n  =  3 biological replicates) except for phages ϕSipho and ϕTB34 where n  =  4 biological replicates. *E*. *coli* MG1655 was used as a reference strain for all EOP measurements. (e) Average burst size assessment for the same strain and plasmid combinations as (c) following infection with ϕSipho at MOI 0.1. Points show mean +/− SEM (n  =  3 biological replicates). Statistical analysis for panel a-c was performed with GraphPad applying unpaired student t test. No significance was detected, unless indicated (*p ≤ 0.05). For panels d-e statistical relevance was measured using one-way ANOVA with Dunnett’s multiple comparison test. No significance was detected, unless indicated (*p ≤ 0.05).

In this strain, the Shield II locus is preceded by a WYL-domain containing protein. Sequence analysis by BLAST and structural predictions indicated this is an homologue of BrxR, a regulator frequently found in defence islands [3,36,37](Fig 1). Shield II alone or Shield II and the upstream BrxR were cloned with their native promoter in a transposon mini-Tn7 vector (S11 Table, see Material and Methods), for site-specific insertion into the chromosome of a *P*. *aeruginosa* PA14 strain with a non-functional CRISPR-Cas system (csy3::LacZ) [38,39]. We determined the EOP of Shield II-harboring strains against several *P*. *aeruginosa* phages and confirmed that Shield II is a bona fide anti-phage system in *Pseudomonas* (Fig 3A). We further observed that the upstream BrxR homologue appeared to repress Shield II-mediated protection (Fig 3A).

Having confirmed activity in the original host species, we returned to heterologous expression of Shield systems in *E*. *coli* for downstream studies. We first tested the ability of ShdA II and Shield II to provide protection against a suite of *E*. *coli* phages. For ShdA II we calculated a fold protection value (ratio between efficiency of plating (EOP) of ShdA II and the EOP of cells carrying empty vector) when challenged with a panel of phages. This ratio provided a quick read-out of ShdA II ability to confer protection against the group of phages tested (Fig 3B). We found that ShdA II conferred resistance to several different phages, which were also susceptible to full-length Shield II (Fig 3C).

Subsequently, we directly compared the EOP on strains producing either the full Shield II system or ShdA II or ShdB II alone when infected with phages ϕSipho, ϕTB34, ϕAlma, ϕNR1 or ϕBaz. We observed that both Shield II and ShdA II conferred a decrease in EOP, albeit with a more modest effect against ϕAlma and ϕBaz (Fig 3C and 3D). Furthermore, Shield II and ShdA II decreased the burst size of ϕSipho, with a more marked effect for ShdA II-harbouring cells (Figs 3E and S7A).

Next, we investigated Shield II and ShdA II-mediated defense in liquid culture against ϕSipho or ϕTB34. Cells harboring Shield II or ShdA II showed better survival than cells carrying empty vector (VC) or ShdB II only, especially at lower MOI values. Additionally, ShdA II- expressing cells showed a better growth rate than Shield II-harboring cells, during phage infection (S7B Fig).

**Fig 4:**
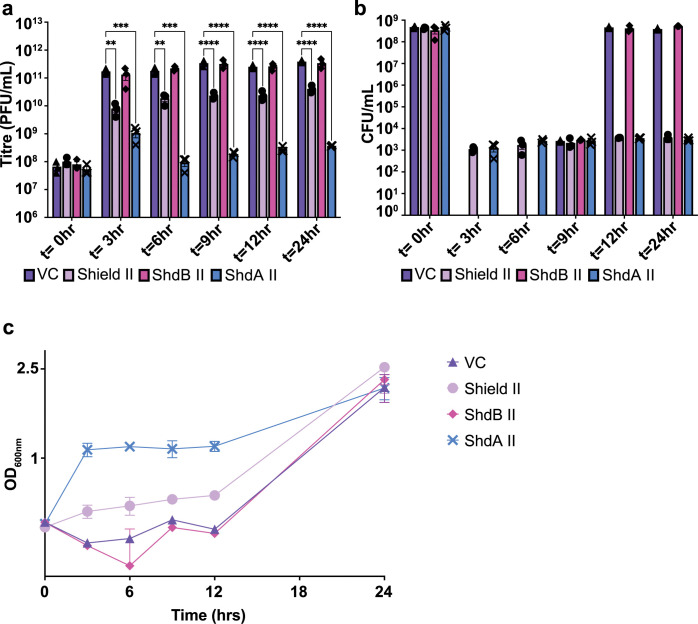
Shield II and ShdA II provide population immunity. *E*. *coli* MG1655 harboring pBAD18 (pBAD18) or the same plasmid encoding Shield II, ShdA II only or ShdB II only were cultured in LB medium containing 0.2% L-arabinose and infected with ϕSipho at MOI 0.1. Following infection, the a) titre (PFU/mL), b) cell counts (CFU/mL) and c) the growth rate (OD_600nm_) of each culture was measured at several timepoints, as shown in panels a-c, over the course of 24 hrs post infection. Points show mean +/− SEM (n  =  3 biological replicates). Statistical analysis for panel a was performed with GraphPad using one-way ANOVA with Dunnett’s multiple comparison test. No significance was detected, unless indicated (*p ≤ 0.05).

To better determine how Shield and ShdA II mediates phage defense, we assessed the progression of ϕSipho infection over the course of 24 hrs, measuring PFU/mL, CFU/mL and OD_600nm_ at several timepoints (Fig 4A–4C). At 3 and 6 hrs post-infection, cells containing vector only or producing ShdB II alone exhibited a complete culture collapse (Fig 4A–4C). At later timepoints, these strains formed small colonies that increased over time, an indication that escape mutants had arisen (Fig 4C). Conversely, cells harboring Shield II or ShdA II displayed an increased growth rate and CFU/mL counts compared to empty vector (Fig 4B). Nevertheless, at later time points the cell counts of Shield II and ShdA II cells were reduced compared to the same strains at t = 0 hrs, whereas their growth in liquid remained unaffected (Fig 4B and 4C).

Quantification of phage produced at each timepoint showed that in the presence of Shield II or ShdA II, ϕSipho replication was hindered despite the reduced cell survival (Fig 4A and 4B). Notably, at 24 hrs post infection the titre of ϕSipho on ShdA II-harboring cells did not show any increase compared to 0 hr (Fig 4A). Conversely, ϕSipho retained partial ability to replicate in cells carrying the full Shield II system (Fig 4A). Efficiency of Centre Of Infection (ECOI) measurements further confirmed that in presence of ShdA II a fewer number of infective centres were formed compared to the full Shield II (S7C Fig).

Taken together, our results show that Shield II and ShdA II promote population-wide immunity by decreasing the cell survival of infected cells (Fig 4B and 4C). Whilst the reduced survival of Shield II- and ShdA II-harboring cells could be part of the immunity mechanism, the phenotypes observed could be partially due to incomplete clearance of the tested phage infection, especially in the case of Shield II (Fig 4A–4C).

From the experiments evaluating the cell survival in liquid cultures, phage replication, burst size, and ECOI measurements of strains carrying Shield II and ShdA II, we consistently noted that presence of ShdA II alone reduces the amount of released phage particles to a greater extent than that observed for cells harboring Shield II. These results suggest that ShdB II may negatively affect the activity of ShdA II (Figs 2, 4 and S7B and S7C).

### ShdA is a membrane protein that has nuclease activity *in vitro*

**Fig 5:**
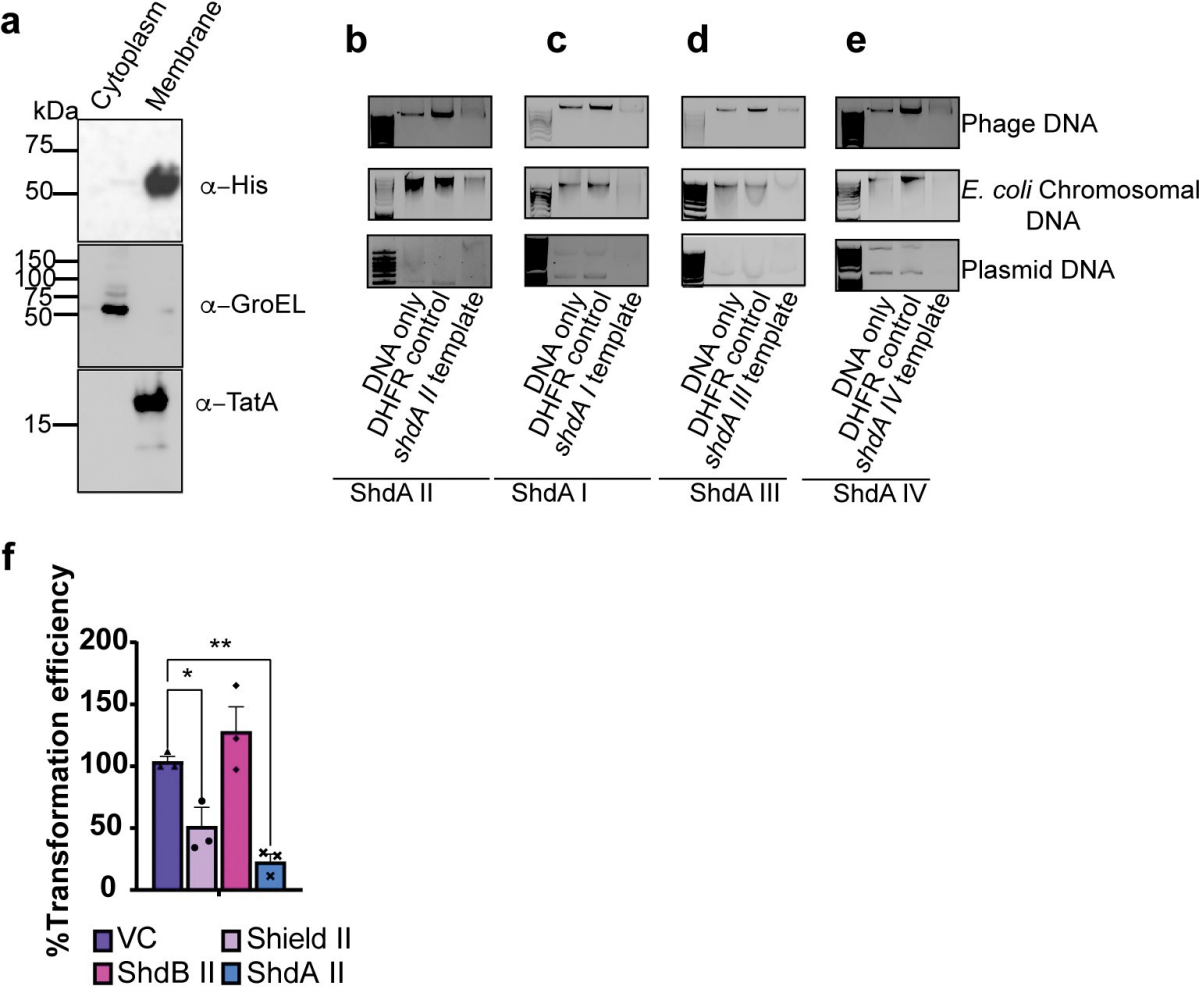
ShdA homologues exhibit nuclease activity *in vitro*. (a) Cells carrying plasmid pBAD18 encoding ShdA II-His_6_ were grown for 5 hrs in the presence of 0.2% L-arabinose. Cells were fractionated to produce soluble and membrane samples and analysed by immunoblot with antibodies to the His6 tag, GroEL (cytoplasmic control) and TatA (membrane control). (b-e) *In vitro* DNAse activity assays using (b) ShdA II_138-524_ (c) ShdA I_140-526_ (d) ShdA III_135-523_ and (e) ShdA IV_135-521_. ShdA proteins and DHFR were synthesised using the cell-free PURExpress kit (NEB). DNAse activity was tested against 10 ng of input DNA. DNA types tested were phage DNA, *E*. *coli* MG1655 chromosomal DNA and plasmid (pSG483) DNA. For ShdA I_140-526_, ShdA III_135-523_ and ShdA II_138-524_ phage DNA was from ϕSipho. For ShdA IV_135-521_ phage DNA was from ϕAlma. (f) Evaluation of transformation efficiency for *E*. *coli* MG1655 carrying empty vector (VC, pBAD18) or the same plasmid encoding Shield subtype II with plasmid DNA. For all panels, 0.2% L-arabinose was added at time zero to induce expression of the encoded genes in pBAD18. Points show mean +/− SEM (n  =  3 biological replicates). Statistical analysis was performed using one-way ANOVA with Dunnett’s multiple comparison test. No significance was detected, unless indicated (*p ≤ 0.05).

Since ShdA proteins are sufficient to elicit protection against phage infection (Fig 2), our next aim was to characterise ShdA activity. Most of ShdA homologues we identified are predicted to have a transmembrane domain at their N-terminus (S9 Table). To confirm that this prediction is correct, we fractionated *E*. *coli* cells producing C-terminally His-tagged ShdA II. Western blot analysis revealed that the protein was only detected in the membrane fraction (Fig 5A). We then asked the question whether membrane-localization of ShdA is essential for its anti-phage activity. To this end we attempted to clone truncated *shdA* genes lacking the 5′ regions encoding the membrane anchor. However, all our efforts were unsuccessful, even when we used tightly repressible vectors, suggesting that the encoded proteins were toxic to *E*. *coli*. Thus, it seems likely that anchoring of ShdA to the membrane modulates its toxicity.

It was previously shown that *E*. *coli* RmuC displays a restriction endonuclease-like fold and is predicted to regulate genome inversion events through DNA cleavage [40]. We therefore next sought to test whether the RmuC domain in ShdA has nuclease activity. As we were unable to clone the truncated *shdA* alleles, we used cell-free synthesis to generate the proteins. Following cell-free synthesis using a template for ShdA II_138-524_, the products caused degradation of phage ϕSipho DNA, *E*. *coli* chromosomal DNA and plasmid DNA (Fig 5B). No degradation was observed when using products generated with the kit-provided template for the control protein dihydrofolate reductase (DHFR) (Fig 5B).

To confirm whether this was a common feature of ShdA proteins, we also tested the ability of *in vitro* synthesised ShdA I_140-526_, ShdA III_135-523_ and ShdA IV_135-521_ to degrade DNA. Whilst the yield of each was low, the products all showed DNase activity (Fig 5C–5E), confirming that DNA degradation is a common feature of ShdA proteins. Interestingly, although we were not able to identify any phages in our collection that were sensitive to Shield III, the ShdA III protein was capable of partially degrading DNA from phage ϕSipho. This suggests that ShdA III is also likely to be active in phage defense, but that our phage collection does not contain representative phage that are sensitive to its activity.

As ShdA homologues exhibited the conserved ability to degrade plasmid DNA, we tested whether Shield II/ShdA II could affect the efficiency of plasmid DNA acquisition during transformation. Indeed, both Shield II and ShdA II decreased transformation efficiency (Fig 5F).

### ShdA induces bacterial nucleoid re-arrangement that is modulated by ShdB

**Fig 6:**
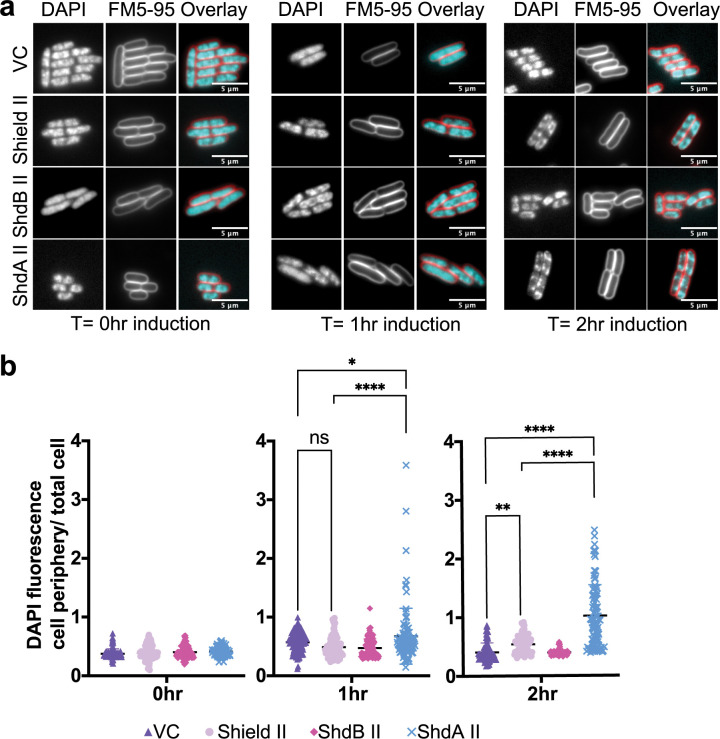
ShdA alters bacterial nucleoid morphology. **(a).** *E*. *coli* MG1655 harboring pBAD18 (pBAD18) or the same plasmid encoding Shield II, ShdA II only or ShdB II only were cultured in LB medium containing 0.2% L-arabinose for 2 hours. At t = 0 hours, t = 1 hour and t = 2 hours an aliquot of each culture was removed and stained with DAPI (for DNA visualisation) and FM 5–95 dye (to stain cell periphery/ outer membranes). Cells were subsequently imaged using fluorescence microscopy (b) The ratio between DAPI fluorescence intensity at the cell periphery and the whole cell was quantified to assess ShdA II-mediated recruitment of DNA at the cell periphery for strains in (a). Points show mean +/− SEM (n  =  100 cells). Statistical analysis was performed using one-way ANOVA with Dunnett’s multiple comparison test. No significance was detected, unless indicated (*p ≤ 0.05).

The *in vitro* nuclease assays (Fig 5B–5E) indicated that the RmuC domain of ShdA has DNase activity that can be active against host chromosomal DNA. To determine whether cells producing ShdA II were anucleate or exhibited other types of aberrant cellular DNA organisation such as changes in DNA condensation, we examined them using the DNA-specific stain 4′,6-diamidino-2-phenylindole (DAPI) and fluorescence microscopy. We observed that over a time-course of ShdA II induction, *E*. *coli* chromosomal DNA was not detectably depleted/degraded, as indicated by the absence of anucleate cells. However, the bacterial nucleoid underwent drastic spatial rearrangement within the cell (Fig 6A). After 2 hr of induction, the nucleoid of ShdA II-expressing cells adopted a distinct morphology and cellular localisation, with an increasingly clustered (S8A Fig) and peripheral DNA distribution pattern (Fig 6A and 6B). This data suggests that ShdA II can disturb the native organisation of the nucleoid and recruit chromosomal DNA to the cell periphery, most likely through direct protein-DNA interactions. Cells producing Shield II showed a less extensive redistribution of DAPI fluorescence, again consistent with a role for ShdB II in negatively impacting the function of ShdA II. As evidenced by unaltered or increased overall DAPI staining levels (S8B Fig), the ShdA-mediated disturbances in host chromosome organisation are not associated with extensive degradation of chromosomal DNA, although DNA fragmentation cannot be ruled out. Measurements of the cell-to-cell correlation of fluorescence variance and integrated intensity allowed to confirm that the increased variance in ShdA- and Shield-expressing cells (S8A Fig) is not due to increased DAPI staining levels (S8C–S8F Fig).

### Accessory Shield components may modulate ShdA-mediated defense or provide additional defence components

To investigate the role of ShdB in the Shield II system we generated a high confidence structural model using AlphaFold (S9A–S9C Fig) and used it to search the Dali server [41,42]. This predicted structural similarity to the metallopeptidase M15 family and to several carboxypeptidases (S9A–S9C Fig and S10 Table). While M15 family proteins are normally involved in peptidoglycan biosynthesis and turnover, ShdB harbors no detectable signal peptide that would direct it to the periplasm. Furthermore, when we provided ShdB II with the signal peptide from *E*. *coli* OmpA protein, to mediate its secretion to the periplasm through the general secretory (Sec) pathway, we observed no effect on the growth of *E*. *coli*, suggesting that the protein is unlikely to cleave peptidoglycan (S10A Fig).

Interestingly, some toxin-antitoxin systems have antitoxins with predicted metallopeptidase activity that exert their effect through degradation of the toxin partner [16,43,44]. We therefore reasoned that ShdB may similarly be a protease whose specific substrate is ShdA. To explore this further we first tested the ability for ShdA II and ShdB II to interact using the bacterial two hybrid assay (BTH) [45]. For this purpose, ShdA II and ShdB II were fused to the two fragments of the catalytic domain of *Bordetella pertussis* adenylate cyclase, carried by the pUT18 and pT25 plasmids [45]. For a detailed description of the bacterial two hybrid principle, see Materials and Methods. We observed an interaction between ShdA II and ShdB II when the T25 fragment of the adenylate cyclase was fused to ShdA II and the UT18 fragment to ShdB II (S10B Fig). The T25 domain is fused to the N-terminus of candidate proteins whereas the UT18 domain is at the C-terminus. Hence, we concluded that the N-terminus of ShdA II and the C-terminus of ShdB are involved in the interaction. Additionally, a self-interaction between ShdB proteins was also observed (S10B Fig). Furthermore, when we modified *shdAII* in the constructs that were used to assess anti-phage activity to produce a protein with a C-terminal hexahistidine epitope, a decrease in ShdA-His_6_ protein levels was observed when co-produced with ShdB II (S10C Fig). Taken together our results are consistent with the hypothesis that ShdB II negatively affects the activity of ShdA II through direct interaction and possibly, degradation of ShdA.

**Fig 7:**
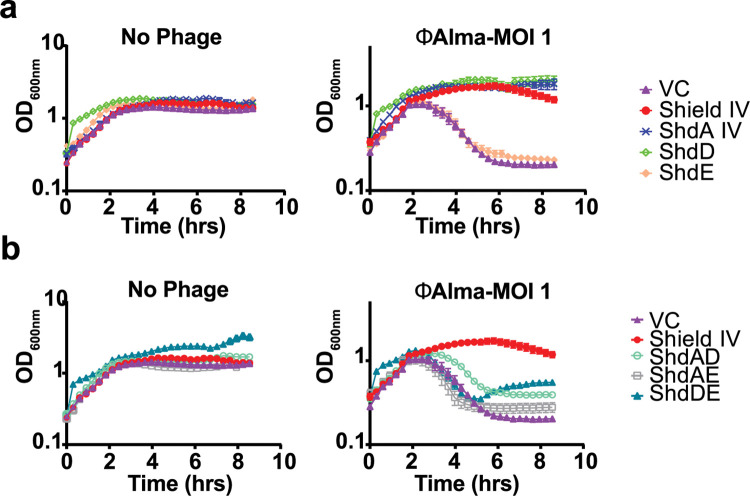
ShdA-associated components in Shield subtypes regulate ShdA activity and/or provide additional defence modules. **(a)**. Growth in liquid LB supplemented with 0.2% L-arabinose of *E*. *coli* MG1655 carrying (VC, pBAD18) or plasmids directing the expression of the Shield IV system (Shield IV), ShdAIV only (ShdAIV), ShdD only (ShdD), ShdE only (ShdE). Strains were infected with MOI = 1 of ϕAlma as described in Methods. Point show mean +/− SEM (n = 3 biological replicates). (b) *E*. *coli* MG1655 carrying (VC, pBAD18) or plasmids directing the expression of the Shield IV system (Shield IV), the ShdA IV and ShdD pair (ShdAD), ShdA IV and ShdE pair (ShdAE), or the ShdA and ShdE pair (ShdDE) were grown in LB medium supplemented with 0.2% L-arabinose and infected with MOI = 1 of ϕAlma as indicated in Methods. Point show mean +/− SEM (n = 3 biological replicates).

Finally, we sought to determine whether a similar regulatory mechanism is found for Shield IV, a subtype that contains the ShdD and ShdE components. Neither of these proteins have any identifiable domains or predicted functions, and share no homology with ShdB. Using phage ϕAlma, we found that ShdD alone was, like ShdA IV, capable of providing protection (Fig 7A), indicating that two phage defense proteins are part of Shield IV. However, while the full Shield IV system was competent to mediate resistance to ϕAlma infection, none of the pairwise combinations conferred any significant protection, suggesting that unlike Shield II, the regulation and fine-tuning of Shield IV anti-phage activity is more complex (Fig 7B).

## Discussion

Here we report the discovery of a previously uncharacterised anti-phage system, Shield, demonstrating that it reduces the release of mature viral particles during phage infection by conferring population-level immunity (Figs 3,4 and 6). The basic defense effector of Shield is ShdA, a membrane-bound protein with a cytoplasmically-located RmuC domain (Figs 3–5). When produced *in vitro*, ShdA RmuC domains have non-specific (endo)nuclease activity and can degrade phage, plasmid and chromosomal DNA (Fig 5). Our inability to clone or express truncated forms of ShdA lacking the membrane anchor suggests that cytoplasmic ShdA is genotoxic and implies that membrane attachment is key to Shield function (Fig 5). In agreement with this, full length ShdA is noticeably less toxic when produced *in vivo*. However, full length ShdA does trigger striking recruitment of chromosomal DNA to the cell periphery *in vivo* (Fig 6). This implies that some feature of the membrane, for example steric hindrance, may regulate ShdA DNase activity. It is worth highlighting that while the observed recruitment of DNA to the membrane is indicative of a ShdA-DNA interactions, the extent of this recruitment is likely exacerbated by high expression levels. Certain other phage defense systems also rely on nuclease activity, for example NucC from the CBASS system, to decrease cell survival and prevent phage propagation [46]. However, examples of anti-phage defense mediated by a co-opted RmuC domain were not reported before.

While ShdA is clearly a central component of Shield-mediated defense, Shield is found as at least four distinct subtypes, with three of them having additional component/s. Two of the three, Shield II and Shield III, contain the ShdB protein. ShdB is a negative regulator of ShdA, and is structurally related to metallopeptidases. Our experimental findings indicate that ShdB interacts with ShdA and likely degrades it to modulate the cellular level of ShdA. Another intriguing, though speculative, possibility is that ShdB can release ShdA from the membrane upon phage infection, thereby activating ShdA’s nuclease activity. The Shield IV system comprises ShdA and two further components, ShdD and ShdE. Like ShdB, both ShdD and ShdE negatively affect ShdA activity, however the situation is more complex because ShdD can also act as a phage defense module when expressed in isolation. Further work would be required to elucidate the roles of ShdD and ShdE in Shield IV-mediated defense.

From the data shown in this work we suggest a model that may account for the activity of the Shield I and Shield II systems. We suggest that, under non-infection conditions, ShdA is present in the membrane and interacts with, but does not degrade, chromosomal DNA. We suggest that the initial stages of phage infection result in changes at the cell envelope, for example phage invasion triggering membrane depolarisation or bulging of the cytoplasmic membrane associated with tail tube fusion [47–49]. Associated changes in membrane physical properties may activate the nuclease activity of membrane-bound ShdA through conformational change or proteolytic cleavage, resulting in phage DNA degradation. Despite the chromosomal rearrangements mediated by ShdA and its promiscuous nuclease activity when dissociated from the membrane *in vitro*, we did not detect host DNA depletion *in vivo* (Figs 6 and S8). Recently, the antiphage protein Hna was shown to cause premature cell death in response to phage infection without causing immediate lysis or liquid culture collapse [50]. The host chromosomal rearrangements caused by ShdA could hinder the ability of the cells to segregate DNA and divide properly, causing the growth defect observed in Fig 4 but, without leading to immediate cell lysis. It is plausible, however, that only during phage infection, a viral trigger will cause ShdA activity to shift from binding and rearranging host chromosome, to degrading both phage and chromosomal DNA.

For the Shield II system, we propose that ShdB oligomers maintain ShdA at a less active state, either through direct interaction or, putatively through controlling the levels of ShdA protein. We suggest that while this level may potentially be sufficient to provide some degree of protection, the ShdB component provides a second checkpoint—for example titration of ShdB away from ShdA by a phage component would allow ShdA to accumulate to higher levels ensuring protection.

Interestingly, we found a subset of ShdA homologues encoded within a DISARM locus, divergent from Shield-related ShdA proteins (S3 Fig). These could potentially represent a fifth additional Shield subtype found associated with DISARM, cooperating with the latter to offer a wider spectrum of protection. A similar cooperation has been observed to some RM systems and other defense systems [2,7,51,52]. Concerted action against invading phages was also observed in a recent study for several newly-identified defense systems [53].

Recent reports highlighted how anti-phage system can adopt similar protein folds/ domains, in different combinations, to mediate immunity [13,14,16,17,29]. A recent example is represented by the CBASS effector NucC, which was also adopted by a subset of CRISPR-Cas systems [46]. Thus, the DISARM-associated ShdA homologues could alternatively represent a case where a DISARM accessory component has acquired a RmuC domain to contribute to phage defense, independently from Shield subtypes. A RmuC-like domain was previously associated with few members of a group of prokaryotic reverse-transcriptases involved in phage defense. However in these cases, the reverse-transcriptase modules were the only ones necessary for defense and the role of RmuC was not explored [54]. Finally, the presence of a RmuC-like domain was also predicted for anti-phage systems PD-T7-1 and PD-T7-5, found in *E*. *coli* [17], but with a much lower probability score and coverage than ShdA II (AN400_RS26690). Furthermore, PD-T7-1 and PD-T7-5 show no sequence similarity to ShdA II, nor a shared predicted fold. Future investigation will establish whether the loci shown in S3 Fig represent another Shield subtype or an example where RmuC domains were independently acquired for bacterial immunity by DISARM components.

Nevertheless, these reports indicate that RmuC domains have been independently acquired for bacterial immunity and our data demonstrate that in Shield systems the RmuC plays a direct and central role in phage inhibition.

To date, despite being functional in both the original host *Pseudomonas* and *E*. *coli*, Shield homologues are only found in *Pseudomonas spp*. While it is difficult to speculate on the reason why this system has not been acquired by other species, other homologues with low sequence but high structural similarities might be present in other genomes and thus future structural studies might aid in finding more ShdA-like proteins with low sequence similarity. We also note that previous studies showed that the number of anti-phage systems per strain, as well as the specific types of systems present can deeply vary within *Pseudomonas* strains [34]. Rare distribution of some anti-phage systems has been reported previously and for some systems, their sparse distribution could be reflective of a compromise between quick development of phage counter-measures, and maintaining the system in circulation within the species ‘pan-immune’ system [1,14,16,34].

The narrow and strain specific distribution of Shield subtypes might be a result of a complex and multi-factorial balance that takes into account the type of mobilisation elements that might be responsible for its horizontal transfer. Other factors could be the potential reduction in cell survival rates and plasmid transfer resulting from acquisition of Shield in more strains/species. This would be especially deleterious should these impacts not be counter-balanced by a significantly stronger phage protection, or if there is a rapid rise of phage counter-measures.

In summary, we describe the discovery and defense mechanism of a previously uncharacterised anti-phage system, which utilises RmuC domains for bacterial immunity. Our study describes a unique defense strategy, adding new information to the complex bacterial immunity landscape.

## Material and methods

### Bacterial strains, plasmids and culture conditions

The strain *P*. *aeruginosa* NCTC 11442 (equivalent to ATCC 33350) was grown overnight at 37°C for chromosomal DNA extraction, whereas of *P*. *aeruginosa* PA14 *csy3*::*lacZ* was grown at 30°C. *E*. *coli* BL21 (DE3) and MG1655 were grown at 37°C on either solid media or liquid culture, shaking at 200 rpm. For liquid growth, Luria broth (LB) was used as the standard medium. For growth on solid media, LB was supplemented with 1.5% (w/v) or with 0.35% (w/v) agar for solid or soft agar, respectively. When required, LB was supplemented with ampicillin (Amp, 50 μg/mL), chloramphenicol (Chl, 25 μg/mL), gentamycin (Gm, 30 μg/mL), isopropyl-β-D-thiogalactopyranoside (IPTG, 0.5 mM), L-arabinose (0.2% w/v) or D-glucose (0.2% w/v). Strains and plasmids used in this study are listed in S11 Table.

### Construction of *P*. *aeruginosa* mutants

Shield II or Shield II with the upstream WYL-containing domain were amplified from *P*. *aeruginosa* NCTC 11442 and cloned into pUC18-mini-Tn7T-Gm [38] using NEBuilder HiFi assembly (S11 and S12 Tables). For each construct the regions upstream and downstream were included to encompass the promoter and terminator of each locus. Four mL of *P*. *aeruginosa* PA14 *csy3*::*lacZ* (S11 and S12 Tables) were washed twice with 300 mM sucrose and resuspended in a final volume of 200 μl of 300 mM sucrose. Cells were electroporated following addition of 50 ng of each pUC18-mini-Tn7T construct and 50 ng of pTNS2 helper plasmid, as previously described [38].

Selection of mutants with a chromosomally integrated construct was performed on LB agar supplemented with 30 μg/ mL of gentamycin.

### Measurement of bacterial growth

Overnight cultures were diluted 1:200 in fresh medium and grown until an OD_600nm_ of 0.3. Cultures were infected with phage at an MOI of 1, 0.1 and 0.01 in a final volume of 200 μL and aliquoted into a 96-well plate. The plate was incubated with continuous shaking in a TECAN infinite nano M+ and absorbance at 600 nm was measured every 20 min.

### DNA manipulation and transformation

The chromosomal DNA of *P*. *aeruginosa* NCTC 11442 was extracted using the GenElute Bacterial Genomic DNA kit (Merck). Plasmid backbones and inserts for cloning were amplified from the purified chromosomal DNA using Q5 High-Fidelity DNA Polymerase (NEB). PCR products and plasmids were purified with Monarch DNA kits (NEB). Overlapping primers for amplification were designed using the NEBuilder assembly tool (https://nebuilderv1.neb.com/) (S12 Table). Plasmids and inserts were assembled using NEBuilder HiFi DNA Assembly (NEB), followed by incubation at 50°C for 20 min. Point mutations or small deletions in pGM34 and pGM71 (S11 and S12 Tables) were performed using the KLD enzyme mix (NEB)

### Phage propagation and lysate preparation

Coliphage lysates were diluted in phage buffer (10 mM Tris–HCl pH 7.4, 10 mM MgSO4, 0.1% gelatin) and propagated in *E*. *coli* DH5α. *Pseudomonas* phages were propagated in *P*. *aeruginosa* PA01 in King’s B media.

For this purpose, neat lysates or their dilutions were added to 200 μL of *E*. *coli* DH5α or in *P*. *aeruginosa* PA01. The mixture was added to 5 mL of soft agar and poured onto LB agar plates. Plates were incubated at 37°C overnight. Following incubation, the top agar containing confluent phage plaques was scraped off and added to 3 mL of phage buffer and 500 μL of chloroform. Samples were vortexed for 2 minutes and then incubated at 4°C for 30 min. Samples were subsequently centrifuged at 4000 x *g* for 20 min and the supernatant collected and added to 100 μL of chloroform for storage.

### Efficiency of plating measurement and fold protection calculation

To measure the efficiency of plating (EOP), 10 μL of phage lysate was added to 200 μL of an overnight culture of *E*. *coli* MG1655 carrying empty pBAD18 or pBAD18 encoding the Shield II system, ShdAII or ShdB II only. Five mL of soft agar was added to each culture and poured onto LB agar plates. As a control strain, plasmid-free MG1655 was used. EOP was measured as the number of PFU mL^-1^ of a test strain divided by the number of PFU/mL of the control strain.

For *P*. *aeruginosa*, 10 μL of phage lysate was added to 100 μL of an overnight culture of *P*. *aeruginosa csy3*::*lacZ* or the same strain carrying chromosomal integrations of either Shield II or WYL-Shield II. Five mL of soft agar was added to each culture and poured onto LB agar plates and incubated at 30°C. *P*. *aeruginosa csy3*::*lacZ* was used as a control and EOP was measured as above.

For the calculation of fold protection, the EOP values calculated for ShdA II and empty vector when challenged with the phage panel indicated in Fig 3A were used. The ratio between ShdA II and empty vector EOP values was used to calculate fold protection.

### Efficiency of centre of infection

*E*. *coli* MG1655 harboring pBAD18 or the same vector encoding Shield II, ShdA II or ShdB II was grown in LB with ampicillin and 0.2% arabinose to an OD_600nm_ of ~ 0.6 and infected with ϕSipho at MOI of 0.1 for ECOI measurement and MOI 2 for cell survival assessment. Infected cells were incubated for 15 min at 37°C shaking at 200 rpm to favour adsorption. Following incubation, cells were washed with ice-cold phage buffer twice and then serially diluted in phage buffer. For ECOI, 10 μL of the desired dilution was added to 200 μL of *E*. *coli* DH5α in 5 mL of soft agar. Infective centres were measured as the number of PFUs on each plate. ECOI measurement are reflective of the proportion of productive infections and generally represent a more absolute measure of phage viability compared to EOP, which is always calculated in relation to a reference strain.

### Measurement of PFU/mL, CFU/mL and growth rate during phage infection

*E*. *coli* MG1655 harboring pBAD18 or the same vector encoding Shield II, ShdA II or ShdB II was grown in LB with ampicillin and 0.2% arabinose to an OD_600nm_ of ~ 0.6 and infected with ϕSipho at MOI of 0.1. An aliquot of each culture was collected at t = 0 hr, t = 3 hr, t = 6 hr, t = 9 hr, t = 12 hr and t = 24 hr and the OD_600nm_ was measured. For each timepoint, the cultures’ aliquots were also serially diluted and plated on LB agar plates to measure CFU/mL or plated onto *E*. *coli* DH5α top lawns to measure phage titre (PFU/mL).

### One step growth curves

To calculate the burst size, *E*. *coli* MG1655 carrying pBAD18 or the same vector encoding Shield II, ShdA II or ShdB II were grown to an OD_600nm_ of ~0.6. ϕSipho was added to an MOI of 0.1. Cultures were incubated at 37°C at 200 rpm and duplicate samples were collected at t  =  5 min, t  =  10 min, t  =  15 min, t  =  20 min, t  =  30 min, t  =  45 min, t  =  60 min, t  =  75 min, t  =  90 min, t  =  105 min and t  =  120 min post infection. For each timepoint, one sample was immediately serially diluted and plated onto *E*. *coli* DH5α top lawns, accounting for free phages and phage-infected cells. For t  =  5 min, a second duplicate sample was collected and treated with chloroform before being plated as above, to represent the adsorption control. The PFU/mL from the adsorption control was subtracted from the average PFU/mL of the bottom plateau shown in S7A Fig (Beginning of infection cycle). Burst size was calculated by dividing this value into the average PFU/mL of the top plateau (S7A Fig) reached at the end of the infection cycle.

### Cell free protein synthesis and Nuclease assay

*In vitro* synthesis of the RmuC domains of ShdA I, ShdA II, ShdA III and ShdA IV was performed using the PURExpress cell-free transcription/translation kit (NEB). Protein synthesis was performed with either 250 ng and 500 ng of DNA template. Synthesis was performed for 4 hr at 37°C according to the manufacturer’s recommendation. Following incubation, 10 mM MgCl_2_ was added to the reaction and the final volume was adjusted to 10 μL. Ribosomes were removed through centrifugation for 60 min at 15,000 rpm at 4°C, through an Amicon Ultracel 0.5mL spin concentrator with a 100 KDa filter (Merck). The flowthrough was collected and the His-tagged PURExpress kit components were removed from the reaction following incubation with Ni-NTA agarose beads (Thermo) for 45 min at 4°C. Agarose beads were removed through centrifugation with Biorad micro Bio-spin columns at 15,000 g for 10 min at 4°C. As a control, the same reactions were performed in parallel with the dihydrofolate reductase (DHFR) control provided by the PURExpress kit.

To test for nuclease activity the in vitro synthesised ShdA proteins or DHFR control were incubated with 20 ng of phage ϕSipho, *E*. *coli* MG1655 chromosome or plasmid pSG483 DNA, followed by agarose gel electrophoresis and staining with GelRed (Cambridge Bioscience).

### Subcellular fractionation

Overnight cultures of MG1655 carrying ShdA II with a C-terminal His_6_ tag were diluted in 25 mL LB containing 0.2% L-arabinose and grown for 5 hrs. Following growth, cells were centrifuged at 4000 x g for 10 min at 4°C. Cells were resuspended in 1 mL of Tris HCl pH 8 and lysed by sonication. Cellular debris were removed by centrifugation at 14,000 x g for 10 min at 4°C. The supernatant was collected and subjected to ultracentrifugation (200,000 x g, 30 min, 4°C). The supernatant was collected, representing the cytoplasmic fraction and the pellet resuspended in 1 mL of Tris HCl pH 8, representing the total membrane fraction. His_6_ tagged proteins and GroEL (used as cytoplasmic control) were detected as above. TatA was used as membrane control protein and detected with anti-TatA serum as previously described [55].

### Bacterial two hybrid assay

ShdA II and ShdB II were fused to the two fragments of adenylate cyclase encoded on plasmids pT25 and pUT18 (SI Appendix, S12 Table) [45]. Plasmids were introduced into *E*. *coli* BTH101 and selected on MacConkey medium (Difco) supplemented with maltose (1%), Amp, 50 μg/mL and Chl 25 μg/mL. Plates were incubated for 48 hrs at 30°C and positive interactions were identified as dark red colonies. When an interaction occurs between two proteins, the two adenylate cyclase fragments are brought in proximity, producing the cAMP signal. This in turns activates the operon involved in maltose catabolism and produces red colonies on MacConkey medium.

### Cell free protein synthesis and Nuclease assay

*In vitro* synthesis of the RmuC domains of ShdA I, ShdA II, ShdA III and ShdA IV was performed using the PURExpress cell-free transcription/translation kit (NEB). Protein synthesis was performed with either 250 ng and 500 ng of DNA template. Synthesis was performed for 4 hr at 37°C according to the manufacturer’s recommendation. Following incubation, 10 mM MgCl_2_ was added to the reaction and the final volume was adjusted to 10 μL. Ribosomes were removed through centrifugation for 60 min at 15,000 rpm at 4°C, through an Amicon Ultracel 0.5mL spin concentrator with a 100 KDa filter (Merck). The flowthrough was collected and the His-tagged PURExpress kit components were removed from the reaction following incubation with Ni-NTA agarose beads (Thermo) for 45 min at 4°C. Agarose beads were removed through centrifugation with Biorad micro Bio-spin columns at 15,000 g for 10 min at 4°C. As a control, the same reactions were performed in parallel with the dihydrofolate reductase (DHFR) control provided by the PURExpress kit.

To test for nuclease activity the in vitro synthesised ShdA proteins or DHFR control were incubated with 20 ng of phage ϕSipho, *E*. *coli* MG1655 chromosome or plasmid pSG483 DNA, followed by agarose gel electrophoresis and staining with GelRed (Cambridge Bioscience).

### Fluorescence microscopy and quantification of DAPI fluorescence

Overnight cultures (5 ml) were diluted into 25 mL LB containing 0.2% arabinose and 50 μg/mL ampicillin and grown for 2 hr. 200 μL of each culture were collected at timepoints t = 0 hr, t = 1 hr and t = 2 hr and stained with 4′,6-diamidino-2-phenylindole (DAPI) at a final concentration of 5 μg /mL and F M5-95(Thermo) at 200 μg /mL. Staining was carried out at 37°C for 15 min and then 2 μL of each culture was transferred on a microscope slide with a pad of 1% UltraPure agarose (Invitrogen) in H_2_O. Imaging was carried out on Nikon Eclipse Ti equipped with CoolLED pE-300^white^ light source, Nikon Plan Apo 100×/1.40 NA Oil Ph3 objective, and Photometrics Prime sCMOS, and Chroma 49008 (Ex 560/40, Dm 585, Em 630/75) filter set for FM 5–95 and Chroma 49000 (Ex 350/50, DM 400, EM 460/50) filter set for DAPI. The images were captured using Metamorph 7.7 (Molecular Devices) and analysed using Fiji [56].

Quantification of DAPI fluorescence was performed using Fiji. In brief, individual cells were identified from thresholded phase contrast images and converted to regions of interest (ROI). The ROI areas associated with the cell periphery were defined as a 3-pixel wide band extending towards the cell interior from the cell boundary which, in turn, was determined by a steep change in phase contrast. These peripheral cell areas represent approx. 200 nm wide sections along the shorter cell axis, flanking the approx. 800 nm wide cell interior region. These whole cell and cell periphery -ROIs, and background-subtracted fluorescence images were then used to quantify the integrated density of DAPI fluorescence signals for the whole cell, and for the respective cell periphery. At last, the ratio of DAPI fluorescence signals between the cell periphery and the whole cell, the integrated density (sum of pixel values) of the whole cell, and the standard deviation thereof were measured, followed by calculation of the variance of cell DAPI fluorescence. The ratio of cell periphery and whole cell fluorescence, the whole cell integrated density itself, and variance thereof were represented as swarm plots, and further analysed by one-way ANOVA with Dunnett’s multiple comparison tests. The potential correlation between the integrated density and variance values for each strain was analysed visually by means of a scatter plot, together with a linear regression between the data points and calculation of the coefficient of determination (R^2^). The linear regression analysis was carried out with GraphPAd Prism 9.

### ShdA II detection

ShdA II with a C-terminal His_6_ tag was expressed from the arabinose inducible plasmid, pBAD18 alone or in presence of ShdB II. Induction was performed with 0.2% L-arabinose for 5 hrs. Following induction, cells were harvested by centrifugation at 14,000 x g for 10 min at 4°C and resuspended in Laemmli buffer. His_6_ tagged proteins were detected with anti-His_6_ primary antibody (Pierce; 1:6,000) and anti-mouse secondary antibody (Biorad; 1:10,000). GroEL was detected with anti-GroEL primary antibody (Pierce; 1:10,000) and anti-rabbit secondary antibody (Biorad; 1:20,000).

### Homology searches and gene neighbourhood analysis

ShdA alignments were generated using MUSCLE (v3.8.1551) [57]. Alignments were used to generate Hidden Markov Models with the HMMER suite (v 3.3.2) [58]. The obtained models were used to query a local Refseq database of *Pseudomonas spp* or all bacterial genomes.The cutoff value was set to a bit score of 30 over the overall sequence/profile comparison. Efetch from the entrez utilities [59] was employed to retrieve an identical protein group report (IPG) for each hit protein obtained from the HMMER searches. Neighboring genes to Shield system were identified using FlaGs version 1.2.7 [35] with a non-redundant ShdA protein set as query. Neighboring genes were analysed using the Defense-finder tool to determine their association to known anti-phage systems.

### Annotation of anti-phage systems

Flanking genes of Shield systems were retrieved using the FlaGs tool [35]. Clustered flanking genes retrieved by FlaGs were subjected to searches against a local PFAM database [60]. Additionally, assembly IDs of genomes encoding Shield homologues, retrieved using Efetch, were used to download genome proteomes. Proteomes were then used as an input to predict anti-phage systems with Defense-finder [34]. Genome neighbourhoods were scanned and anti-phage system predictions were manually curated by comparing Defense-finder results with PFAM predictions [60]. For multi-gene loci where PFAM and Defense-finder predictions were discordant, the prediction that best matched their operon organisation was chosen.

### Protein function prediction

Where possible, protein function predictions were performed using a local PFAM database [60]. Protein structures were predicted using Alphafold and the Dali server (50, 51). Presence of signal peptides and transmembrane domains were predicted using DeepTMHMM and SignalP 6.0 [61,62]. [60]

### Phylogenetic analysis

The alignment of ShdA proteins was used to build a maximum likelihood phylogenetic tree with IQTREE (v 2.1.4) [63] with 1000 ultrafast bootstraps. Trees were plotted and annotated in iTOL [64].

## Supporting information

S1 Table Known anti-phage systems in *Pseudomonas* species predicted by Defense-finder. (XLSX)

S2 Table Identical protein group report for ShdA homologues across Shield subtypes. (XLSX)

S3 Table FlaGs output reporting flanking genes of Shield subtypes. ShdA homologues were used as a query for FlaGs.
(XLSX)

S4 Table Informations about flanking genes for each ShdA query. For each unique ShdA protein identifier, FlaGs authomatically chose an Assembly and the identifier is shown in S2 Table.
(XLSX)

S5 Table Known defence systems predicted using PFAM and Defense-finder for FlaGs-clustered genes. (XLSX)

S6 Table Defense-finder predictions for all genomes that encode Shield subtypes. (XLSX)

S7 Table FlaGs output reporting flanking genes of DISARM-associated ShdA homologues. (XLSX)

S8 Table Identical protein group report for ShdA encoded within DISARM-like operons. (XLSX)

S9 Table TMHMM prediction for ShdA homologues. (XLSX)

S10 Table List of hits from Dali server searches for AN400_RS26695 predicted. (XLSX)

S11 Table Strains and plasmids used in this study. (DOCX)

S12 Table Oligonucleotide primers and additional details for plasmid construction. (DOCX)

S1 Fig Definition of Shield subtypes. FlaGs output representing the genomic neighbourhood of *shdA* genes belonging to different subtypes. The spectrum of genomes encoding these homologues is reported in S2–S5 Tables. FlaGs-grouped genes are numbered and coloured by FlaGs according to their association to a certain cluster. FlaGs-numbering of clustered genes is reported in S4 Table. Clustered genes were annotated as belonging to a specific antiphage system using PFAM and Defense-finder. These annotations are reported in S4 Table. *shdA* genes are coloured in blue and partner genes have a coloured outline as indicated on the figure.
(PDF)

S2 Fig Alignment of one representative ShdA homologue from each Shield subtype. Representative alignments of ShdA homologues across Shield subtypes. One representative ShdA homologue was chosen for each Shield subtype and aligned using MUSCLE. A full alignment, involving all homologues, is shown in S2 Fig.
(PDF)

S3 Fig Alignment of ShdA homologues from different subtypes. Alignment of ShdA homologues using MUSCLE. The alignment was visualised in Boxshade and coloured by percentage of identity.
(PDF)

S4 Fig ShdA homologues are encoded in DISARM loci. **(a)** Schematic representation of ShdA homologues encoded within DISARM operons. The full set of DISARM-associated ShdA loci is shown in S4 Fig. **(b)** Phylogenetic tree based on the ShdA homologues from S1 Fig in addition to DISARM-associated ShdA. Coloured blocks were used to show ShdA homologues belonging to distinct Shield subtypes or DISARM-like loci.
(PDF)

S5 Fig Distant ShdA homologues can be encoded in DISARM-like operons. FlaGs output representing the genomic neighbourhood of distant ShdA homologues shows that these can be encoded within DISARM-like operons. Neighbouring genes are clustered together based on similarity and each cluster is numbered. Definition of neighbouring gene clusters is reported in S8 Table.
(PDF)

S6 Fig Alignment of ShdA homologues from different Shield subtypes and DISARM- associated ShdA. Alignment of ShdA II homologues using MUSCLE. The alignment was coloured in Boxshade by percentage of identity. Sequence conservation between Shield- and DISARM-associated ShdA homologues is still present but is reduced to what observed in S2 Fig.
(PDF)

S7 Fig ShdA II reduces the phage burden. **(a)** One-step growth curve for *E*. *coli* MG1655 carrying pBAD 18 (VC,) or the same plasmid encoding Shield II, ShdA II only or ShdB II only when infected with φSipho. Strains were grown in LB supplemented with 0.2% L- arabinose and infected at time zero with MOI = 0.1. PFU/mL were evaluated at t = 5 min, t = 10 min, t = 15 min, t = 20 min, t = 30 min, t = 45 min, t = 60 min, t = 75 min, t = 90 min, t = 105 min and t = 120 min. **(b)** Growth curves of *E*. *coli* MG1655 carrying pBAD 18 (VC,) or the same plasmid encoding Shield II, ShdA II only or ShdB II only. Strains were grown in LB supplemented with 0.2% L-arabinose and infected at time zero with MOI = 1, MOI = 0.1, MOI = 0.01 of φSipho and φTB34. **(c)** Evaluation of efficiency of centre of infection (ECOI) for *E*. *coli* MG1655 carrying pBAD 18 (VC) or the same plasmid encoding Shield II, ShdA II only or ShdB II only when challenged with phage φSipho at MOI = 0.1. grown in LB supplemented with 0.2% L-arabinose and assays were performed as described in Material and Methods. For all panels, points show mean +/- SEM (n = 3 biological replicates). Statistical relevance was measured using one-way ANOVA with Dunnett’s multiple comparison test. No significance was detected unless indicated (*p≤0.05).
(PDF)

S8 Fig ShdA II mediates chromosome re-arrangements but does not lead to degradation *in vivo*. The same region of interest (ROI) used for quantification of the DAPI fluorescence intensity at the cell periphery shown in Fig 6B were used to quantify the **a)** the variance of pixel intensity and **b)** the integrated pixel intensity of the cellular DAPI signals for the t = 2 hrs timepoint (see Material and Methods for details). Statistical analysis was performed using one-way ANOVA with Dunnett’s multiple comparison test. No significance was detected, unless indicated (*p ≤ 0.05). **(c-f)** To test if the variance of pixel intensity observed in cell expressing ShdA or Shield is influenced by the concurrent increased in DAPI staining, the correlation was analysed. The graphs depict the cell-to-cell correlation of fluorescence variance and integrated intensity, together with a linear regression and its R^2^ values. Only a very low correlation was observed with between DAPI intensity and its variance throughout the tested strains, thus ruling out that the increased variance observed ShdA or Shield -expressing cells is due to higher overall DAPI staining levels.
(PDF)

S9 Fig ShdB II structural prediction suggests a peptidase M15 fold. **(a)** Alphafold predicted structure of ShdB II was used for Dali predictions (S4 Table), showing it harbours a predicted peptidase M15 domain. **(b)** Local Distance Difference Test (lDDT) relative to ShdB II predicted structure. IDDT shows a per-residue measure of local confidence for the prediction and it is high across the whole ShdB II structure. **(c)** Predicted Aligned Error (PAE) for ShdB II Alphafold-predicted structure. PAE reports the expected error at each residue position. For ShdB II structure PAE was low across the whole sequence.
(PDF)

S10 Fig ShdB is a probable peptidase that negatively affects the cellular level of ShdA. **(a)** ShdB II expression with a signal peptide does not impair cell growth. Growth in liquid LB media of *E*. *coli* MG1655 carrying (VC, pBAD18), ShdB II or ShdB II with an OmpA signal sequence (sp-ShdB II). Expression was repressed with 0.2% D-glucose or induced with addition of 0.2% L-arabinose. Points show mean +/− SEM (n = 3 biological replicates). **(b)** Analysis of *E*. *coli* BTH101 carrying combinations of ShdA II and ShdB II, when cloned in bacterial two-hybrid vectors pUT18 or pT25 vectors as indicated. Cloning in pUT18 and pT25 allows fusion of candidate proteins to the UT18 and T25 fragments of the adenylate cyclase. Upon interaction of candidate proteins, the adenylate cyclase is reconstituted, producing the cAMP signal, in turn activating the maltose catabolism operon, resulting in red colonies on MacConkey medium. The empty pUT18 or pT25 vectors were used as negative controls, while the interaction between NarG and NarJ proteins was employed as a positive control (58). **(c)** ShdA II-His_6_ or ShdA II-His_6_ + ShdB II were expressed from an arabinose-inducible plasmid pBAD18. and ShdA II-His_6_ levels were assessed by western blot analysis (See Material and Methods). GroEL was used as loading control.
(PDF)
