# Supplementary material for: The novel anti-phage system Shield co-opts an RmuC domain to mediate phage defense across Pseudomonas species
Source: PLoS Genet. 2023 Jun 5;19(6):e1010784. doi: 10.1371/journal.pgen.1010784 (PMC10270631; doi:10.1371/journal.pgen.1010784)
Supplement: S11 Table — (DOCX) [file pgen.1010784.s011.docx]

xS11 Table. Strains and plasmids used in this study

| **Name** | **Description** | **Reference** |  |
| --- | --- | --- | --- |
| **Strains** |  |  | |
| ***Pseudomonas aeruginosa*** |  |  | |
| NCTC 11442 | *Pseudomonas aeruginosa* strain NCTC 11442/ATCC 33350 | NCTC | |
| - | *P. aeruginosa* UCBPP-PA14 *csy3::LacZ* | [1] | |
| GM11 | Coding sequence for Shield II and WYL-containing protein (AN400_RS26685, AN400_RS26690 and AN400_RS26695) from *P. aeruginosa* ATCC33350 integrated in *P. aeruginosa* UCBPP-PA14 *csy3::LacZ* chromosome through pUC18-mini-Tn7T-Gm | This study | |
| GM12 | Coding sequence for Shield II (AN400_RS26690 and AN400_RS26695) from *P. aeruginosa* ATCC33350 integrated in *P. aeruginosa* UCBPP-PA14 *csy3::LacZ* chromosome through pUC18-mini-Tn7T-Gm | This study | |
| ***Escherichia coli*** |  |  | |
| MG1655 | Wild type (model K-12 strain) | [2] | |
| DH5α | Cloning strain, F– φ80lacZΔM15 Δ(lacZYA-argF)U169 recA1 endA1 hsdR17(rK–, mK+) phoA supE44 λ–thi-1 gyrA96 relA1 | New England Biolabs | |

| **Plasmids** |  |  |
| --- | --- | --- |

| pBAD18 | Arabinose-inducible expression vector (Kn^R^); gene of interest is cloned downstream of the P*_ara_* promoter | [3] |
| --- | --- | --- |
| pUT18 | Bacterial Two Hybrid plasmid (for fusion of target protein with C-terminal T18 fragment of CyaA; Amp^R^) | [4] |
| pT25 | Bacterial Two Hybrid plasmid (for fusion of target protein with Nterminal T25 fragment of CyaA; Cm^R^) | [4] |
| pGM34 | Coding sequence for Shield II (AN400_RS26690 and AN400_RS26695) from *P. aeruginosa* ATCC33350 in pBAD18 | This study |
| pGM42 | Coding sequence for ShdB II (AN400_RS26695) from *P. aeruginosa* ATCC33350 in pBAD18 | This study |
| pGM43 | Coding sequence for ShdA II (AN400_RS26690) from *P. aeruginosa* ATCC33350 in pBAD18 | This study |
| pGM122 | Coding sequence for ShdA I (DL351_RS13220) from *P. aeruginosa* BH9 in pBAD18 | This study |
| pGM133 | Coding sequence for Shield III (EQ826_RS12590, EQ826_RS12595 and EQ826_12600) from *P. mendocina* FFL34 in pBAD18 | This study |
| pGM134 | Coding sequence for ShdA III (EQ826_RS12590) from *P. mendocina* FFL34 in pBAD18 | This study |
| pGM126 | Coding sequence for Shield IV (A9179_RS12860, A9179_RS12855, A9179_RS12850) from *P.* *alcaligenes* AVO110 in pBAD18 | This study |
| pGM127 | Coding sequence for ShdA IV (A9179_RS12860) from *P.* *alcaligenes* AVO110 in pBAD18 | This study |
| pGM128 | Coding sequence for ShdD (A9179_RS12855) from *P.* *alcaligenes* AVO110 in pBAD18 | This study |
| pGM139 | Coding sequence for ShdE (A9179_RS12850) from *P.* *alcaligenes* AVO110 in pBAD18 | This study |
| pGM130 | Coding sequence for ShdAD (A9179_RS12860, A9179_RS12855) from *P.* *alcaligenes* AVO110 in pBAD18 | This study |
| pGM131 | Coding sequence for ShdAE(A9179_RS12860, A9179_RS12850) from *P.* *alcaligenes* AVO110 in pBAD18 | This study |
| pGM132 | Coding sequence for ShdDE (A9179_RS12855, A9179_RS12850) from *P.* *alcaligenes* AVO110 in pBAD18 | This study |
| pGM107 | Coding sequence for ShdA II (AN400_RS26690) fused with a C-terminal His_6_ tag and coding sequence of ShdB II (AN400_RS26695) in pBAD18 | This study |
| pGM116 | Coding sequence for ShdA II (AN400_RS26690) fused with a C-terminal His_6_ tag in pBAD18 | This study |
| pGM117 | Coding sequence for ShdA II (AN400_RS26690) in pUT18 | This study |
| pGM118 | Coding sequence for ShdB II (AN400_RS26695) in pUT18 | This study |
| pGM119 | Coding sequence for ShdA II (AN400_RS26690) in pT25 | This study |
| pGM120 | Coding sequence for ShdB II (AN400_RS26695) in pT25 | This study |
| pUT18-NarG | Coding sequence of NarG in pUT18 | [5] |
| pT25-NarJ | Coding sequence of NarJ in pT25 | [5] |
| pUC18-mini-Tn7T-Gm | Vector containing a mini-Tn7 system with single attTn7 site for integration in *P. aeruginosa* chromosome | [6] |
| pGM197 | Coding sequence of WYL-domain containing protein and Shield II from from *P. aeruginosa* ATCC33350 in pUC18-mini-Tn7T-Gm | This study |
| pGM198 | Coding sequence of Shield II in pUC18-mini-Tn7T-Gm | This study |
| pTNS2 | Helper plasmid for integration of pUC18-mini-Tn7T-Gm | [6] |

**REFERENCES**

1. Landsberger M, Gandon S, Meaden S, Rollie C, Chevallereau A, Chabas H, et al. Anti-CRISPR Phages Cooperate to Overcome CRISPR-Cas Immunity. Cell. 2018;174: 908. doi:10.1016/j.cell.2018.05.058

2. Blattner FR, Plunkett G, Bloch CA, Perna NT, Burland V, Riley M, et al. The Complete Genome Sequence of Escherichia coli K-12. Science. 1997;277: 1453–1462. doi:10.1126/science.277.5331.1453

3. Guzman LM, Belin D, Carson MJ, Beckwith J. Tight regulation, modulation, and high-level expression by vectors containing the arabinose PBAD promoter. J Bacteriol. 1995;177: 4121–4130. doi:10.1128/jb.177.14.4121-4130.1995

4. Karimova G, Pidoux J, Ullmann A, Ladant D. A bacterial two-hybrid system based on a reconstituted signal transduction pathway. Proc Natl Acad Sci. 1998;95: 5752–5756. doi:10.1073/pnas.95.10.5752

5. Ize B, Coulthurst SJ, Hatzixanthis K, Caldelari I, Buchanan G, Barclay EC, et al. Remnant signal peptides on non-exported enzymes: implications for the evolution of prokaryotic respiratory chains. Microbiology. 2009;155: 3992–4004. doi:10.1099/mic.0.033647-0

6. Choi K-H, Schweizer HP. mini-Tn7 insertion in bacteria with single attTn7 sites: example Pseudomonas aeruginosa. Nat Protoc. 2006;1: 153–161. doi:10.1038/nprot.2006.24
