# Supplementary material for: The novel anti-phage system Shield co-opts an RmuC domain to mediate phage defense across Pseudomonas species
Source: PLoS Genet. 2023 Jun 5;19(6):e1010784. doi: 10.1371/journal.pgen.1010784 (PMC10270631; doi:10.1371/journal.pgen.1010784)
Supplement: S12 Table — (DOCX) [file pgen.1010784.s012.docx]

Table S12. Oligonucleotide primers and additional details for plasmid construction.

| **Plasmid** | **Sequence of relevant primers (5’-3’)**^a^ | **Description** |
| --- | --- | --- |
| pGM34 | GGTACCCGGGGATCCTC | Forward primer to clone AN400_RS26690 and AN400_RS26695 in pBAD18 by NEBuilder HiFi DNA Assembly. Primer is used to linearise vector |
|  | GAATTCGCTAGCCCAAAAAAAC | Reverse primer to clone AN400_RS26690 and AN400_RS26695 in pBAD18 by NEBuilder HiFi DNA Assembly. Primer is used to linearise vector |
|  | GTTTTTTTGGGCTAGCGAATTCATGATTGGATTGTCGTGGATAGTGATTAG | Forward primer to clone AN400_RS26690 and AN400_RS26695 in pBAD18 by NEBuilder HiFi DNA Assembly. Primer is used to amplify genes of interest |
|  | AGGATCCCCGGGTACCCTAGTTGAGACTCGCCAACCATGGGC | Reverse primer to clone AN400_RS26690 and AN400_RS26695 in pBAD18 by NEBuilder HiFi DNA Assembly. Primer is used to amplify genes of interest |
| pGM43 | GGTACCCGGGGATCCTC | Forward primer to clone AN400_RS26690 in pBAD18 by NEBuilder HiFi DNA Assembly. Primer is used to linearise vector |
|  | GAATTCGCTAGCCCAAAAAAAC | Reverse primer to clone AN400_RS26690 in pBAD18 by NEBuilder HiFi DNA Assembly. Primer is used to linearise vector |
|  | GTTTTTTTGGGCTAGCGAATTCATGATTGGATTGTCGTGGATAGTGATTAG | Forward primer to clone AN400_RS26690 in pBAD18 by NEBuilder HiFi DNA Assembly. Primer is used to amplify genes of interest |
|  | AGGATCCCCGGGTACCTCACGCCTGCTGCTCAG | Reverse primer to clone AN400_RS26690 in pBAD18 by NEBuilder HiFi DNA Assembly. Primer is used to amplify genes of interest |
| pGM42 | GGTACCCGGGGATCCTC | Forward primer to clone AN400_RS26695 in pBAD18 by NEBuilder HiFi DNA Assembly. Primer is used to linearise vector |
|  | GAATTCGCTAGCCCAAAAAAAC | Reverse primer to clone AN400_RS26695 in pBAD18 by NEBuilder HiFi DNA Assembly. Primer is used to linearise vector |
|  | CGTTTTTTTGGGCTAGCGAATTCATGAAAAAACCAGCAACGGTAC | Forward primer to clone AN400_RS26690 in pBAD18 by NEBuilder HiFi DNA Assembly. Primer is used to amplify genes of interest |
|  | AGGATCCCCGGGTACCTCACGCCTGCTGCTCAG | Reverse primer to clone AN400_RS26695 in pBAD18 by NEBuilder HiFi DNA Assembly. Primer is used to amplify genes of interest |
| pGM122 * | \| GAATTCGCTAGCCCAAAAAAAC \| \| --- \| \|  \| | Forward primer to clone DL351_RS13220 pBAD18 by NEBuilder HiFi DNA Assembly. Primer is used to linearise vector |
|  | GGTACCCGGGGATCCTCTAG | Reverse primer to clone DL351_RS13220 in pBAD18 by NEBuilder HiFi DNA Assembly. Primer is used to linearise vector |
|  | CGTTTTTTTGGGCTAGCGAATTCATGTCCTGGGCAGTAATG | Forward primer to clone DL351_RS13220 in pBAD18 by NEBuilder HiFi DNA Assembly. Primer is used to amplify genes of interest |
|  | CTAGAGGATCCCCGGGTACCTTACTGCGCCATTTCGCTG | Reverse primer to clone DL351_RS13220 in pBAD18 by NEBuilder HiFi DNA Assembly. Primer is used to amplify genes of interest |
| pGM133 * | GAATTCGCTAGCCCAAAAAAAC | Forward primer to clone EQ826_RS12590, EQ826_RS12595 and EQ826_12600 in pBAD18 by NEBuilder HiFi DNA Assembly. Primer is used to linearise vector |
|  | GGTACCCGGGGATCCTCTAG | Reverse primer to clone EQ826_RS12590, EQ826_RS12595 and EQ826_12600 in pBAD18 by NEBuilder HiFi DNA Assembly. Primer is used to linearise vector |
|  | CGTTTTTTTGGGCTAGCGAATTCATGTCCTGGGCAGTAATG | Forward primer to clone EQ826_RS12590, EQ826_RS12595 and EQ826_12600 in pBAD18 by NEBuilder HiFi DNA Assembly. Primer is used to amplify genes of interest |
|  | CTAGAGGATCCCCGGGTACCTCAGCCCAGGCTAGCTAG | Reverse primer to clone EQ826_RS12590, EQ826_RS12595 and EQ826_12600 in pBAD18 by NEBuilder HiFi DNA Assembly. Primer is used to amplify genes of interest |
| pGM134 * | GAATTCGCTAGCCCAAAAAAAC | Forward primer to clone EQ826_RS12590 in pBAD18 by NEBuilder HiFi DNA Assembly. Primer is used to linearise vector |
|  | GGTACCCGGGGATCCTCTAG | Reverse primer to clone EQ826_RS12590 in pBAD18 by NEBuilder HiFi DNA Assembly. Primer is used to linearise vector |
|  | CGTTTTTTTGGGCTAGCGAATTCATGTCCTGGGCAGTAATGGG | Forward primer to clone EQ826_RS12590in pBAD18 by NEBuilder HiFi DNA Assembly. Primer is used to amplify genes of interest |
|  | CTAGAGGATCCCCGGGTACCTGCTTCCTCCTGCGCCTC | Reverse primer to clone EQ826_RS12590in pBAD18 by NEBuilder HiFi DNA Assembly. Primer is used to amplify genes of interest |
| pGM126 * | GAATTCGCTAGCCCAAAAAAAC | Forward primer to clone A9179_RS12860, A9179_RS12855 and A9179_RS12850 in pBAD18 by NEBuilder HiFi DNA Assembly. Primer is used to linearise vector |
|  | GGTACCCGGGGATCCTCTAG | Reverse primer to clone A9179_RS12860, A9179_RS12855 and A9179_RS12850 in pBAD18 by NEBuilder HiFi DNA Assembly. Primer is used to linearise vector |
|  | CGTTTTTTTGGGCTAGCGAATTCATGTCTTGGGGAGTAGCGG | Forward primer to clone A9179_RS12860, A9179_RS12855 and A9179_RS12850 in pBAD18 by NEBuilder HiFi DNA Assembly. Primer is used to amplify genes of interest |
|  | CTAGAGGATCCCCGGGTACCTCATCCTTGGCCGGCGAG | Reverse primer to clone A9179_RS12860, A9179_RS12855 and A9179_RS12850 in pBAD18 by NEBuilder HiFi DNA Assembly. Primer is used to amplify genes of interest |
| pGM127 * | GAATTCGCTAGCCCAAAAAAAC | Forward primer to clone A9179_RS12860 in pBAD18 by NEBuilder HiFi DNA Assembly. Primer is used to linearise vector |
|  | GGTACCCGGGGATCCTCTAG | Reverse primer to clone A9179_RS12860 in pBAD18 by NEBuilder HiFi DNA Assembly. Primer is used to linearise vector |
|  | CGTTTTTTTGGGCTAGCGAATTCATGTCTTGGGGAGTAGCGG | Forward primer to clone A9179_RS12860 in pBAD18 by NEBuilder HiFi DNA Assembly. Primer is used to amplify genes of interest |
|  | CTAGAGGATCCCCGGGTACCTTACTGCGGCAATTCGCTG | Reverse primer to clone A9179_RS12860in pBAD18 by NEBuilder HiFi DNA Assembly. Primer is used to amplify genes of interest |
| pGM128 * | GAATTCGCTAGCCCAAAAAAAC | Forward primer to A9179_RS12855 in pBAD18 by NEBuilder HiFi DNA Assembly. Primer is used to linearise vector |
|  | GGTACCCGGGGATCCTCTAG | Reverse primer to clone A9179_RS12855 in pBAD18 by NEBuilder HiFi DNA Assembly. Primer is used to linearise vector |
|  | CGTTTTTTTGGGCTAGCGAATTCATGCAGGAGTCGTACGACTTTG | Forward primer to clone A9179_RS12855 in pBAD18 by NEBuilder HiFi DNA Assembly. Primer is used to amplify genes of interest |
|  | CTAGAGGATCCCCGGGTACCCTACCAACCTCGACGGGC | Reverse primer to clone A9179_RS12855 in pBAD18 by NEBuilder HiFi DNA Assembly. Primer is used to amplify genes of interest |
| pGM129 * | GAATTCGCTAGCCCAAAAAAAC | Forward primer to clone A9179_RS12850 in pBAD18 by NEBuilder HiFi DNA Assembly. Primer is used to linearise vector |
|  | GGTACCCGGGGATCCTCTAG | Reverse primer to clone A9179_RS12850 in pBAD18 by NEBuilder HiFi DNA Assembly. Primer is used to linearise vector |
|  | CGTTTTTTTGGGCTAGCGAATTCATGCAGTCGTTGCATATAAGACAGC | Forward primer to clone A9179_RS12850 in pBAD18 by NEBuilder HiFi DNA Assembly. Primer is used to amplify genes of interest |
|  | CTAGAGGATCCCCGGGTACCTCATCCTTGGCCGGCGAG | Reverse primer to clone A9179_RS12850 in pBAD18 by NEBuilder HiFi DNA Assembly. Primer is used to amplify genes of interest |
| pGM130 * | GAATTCGCTAGCCCAAAAAAAC | Forward primer to clone A9179_RS12860 and A9179_RS12855 in pBAD18 by NEBuilder HiFi DNA Assembly. Primer is used to linearise vector |
|  | GGTACCCGGGGATCCTCTAG | Reverse primer to clone A9179_RS12860 and A9179_RS12855 in pBAD18 by NEBuilder HiFi DNA Assembly. Primer is used to linearise vector |
|  | CGTTTTTTTGGGCTAGCGAATTCATGTCTTGGGGAGTAGCGG | Forward primer to clone A9179_RS12860 and A9179_RS12855 in pBAD18 by NEBuilder HiFi DNA Assembly. Primer is used to amplify genes of interest |
|  | CTAGAGGATCCCCGGGTACCCTACCAACCTCGACGGGC | Reverse primer to clone A9179_RS12860 and A9179_RS12855 in pBAD18 by NEBuilder HiFi DNA Assembly. Primer is used to amplify genes of interest |
| pGM131 * | GAATTCGCTAGCCCAAAAAAAC | Forward primer to clone A9179_RS12860 and A9179_RS12850 in pBAD18 by NEBuilder HiFi DNA Assembly. Primer is used to linearise vector |
|  | GGTACCCGGGGATCCTCTAG | Reverse primer to clone A9179_RS12860 and A9179_RS12850 in pBAD18 by NEBuilder HiFi DNA Assembly. Primer is used to linearise vector |
|  | CGTTTTTTTGGGCTAGCGAATTCATGTCTTGGGGAGTAGCGG | Forward primer to clone A9179_RS12860 and A9179_RS12850 in pBAD18 by NEBuilder HiFi DNA Assembly. Primer is used to amplify A9179_RS12860 |
|  | ACGACTGCATTTACTGCGGCAATTCGCTG | Reverse primer to A9179_RS12860 and A9179_RS12850 in pBAD18 by NEBuilder HiFi DNA Assembly. Primer is used to amplify A9179_RS12860 |
|  | GCCGCAGTAAATGCAGTCGTTGCATATAAGACAGC | Forward primer to A9179_RS12860 and A9179_RS12850 in pBAD18 by NEBuilder HiFi DNA Assembly. Primer is used to amplify A9179_RS12850 |
|  | CTAGAGGATCCCCGGGTACCTCATCCTTGGCCGGCGAG | Reverse primer to A9179_RS12860 and A9179_RS12850 in pBAD18 by NEBuilder HiFi DNA Assembly. Primer is used to amplify A9179_RS12850 |
| pGM132 * | GAATTCGCTAGCCCAAAAAAAC | Forward primer to clone A9179_RS12855 and A9179_RS12850 in pBAD18 by NEBuilder HiFi DNA Assembly. Primer is used to linearise vector |
|  | GGTACCCGGGGATCCTCTAG | Reverse primer to clone A9179_RS12855 and A9179_RS12850 in pBAD18 by NEBuilder HiFi DNA Assembly. Primer is used to linearise vector |
|  | CGTTTTTTTGGGCTAGCGAATTCATGCAGGAGTCGTACGACTTTG | Forward primer to clone A9179_RS12855 and A9179_RS12850 in pBAD18 by NEBuilder HiFi DNA Assembly. Primer is used to amplify genes of interest |
|  | CTAGAGGATCCCCGGGTACCTCATCCTTGGCCGGCGAG | Reverse primer to clone A9179_RS12855 and A9179_RS12850 in pBAD18 by NEBuilder HiFi DNA Assembly. Primer is used to amplify genes of interest |
| pGM117 | AGTCGACCTGCAGGCATG | Forward primer to clone AN400_RS26690 in pUT18 by NEBuilder HiFi DNA Assembly. Primer is used to linearise vector |
|  | CTAGAGGATCCCCGGGTAC | Reverse primer to clone AN400_RS26690 in pUT18 by NEBuilder HiFi DNA Assembly. Primer is used to linearise vector |
|  | TGCATGCCTGCAGGTCGACTATGATTGGATTGTCGTGGATAGTGATTAGC | Forward primer to clone AN400_RS26690 in pUT18 by NEBuilder HiFi DNA Assembly. Primer is used to amplify gene of interest |
|  | GGTACCCGGGGATCCTCTAGCGCCTGCTGCTCAGCTGG | Reverse primer to clone AN400_RS26690 in pUT18 by NEBuilder HiFi DNA Assembly. Primer is used to amplify genes of interest |
| pGM118 | AGTCGACCTGCAGGCATG | Forward primer to clone AN400_RS26695 in pUT18 by NEBuilder HiFi DNA Assembly. Primer is used to linearise vector |
|  | CTAGAGGATCCCCGGGTAC | Reverse primer to clone AN400_RS26695 in pUT18 by NEBuilder HiFi DNA Assembly. Primer is used to linearise vector |
|  | TGCATGCCTGCAGGTCGACTATGAAAAAACCAGCAACGGTAC | Forward primer to clone AN400_RS26695 in pUT18 by NEBuilder HiFi DNA Assembly. Primer is used to amplify gene of interest |
|  | GGTACCCGGGGATCCTCTAGGTTGAGACTCGCCAACCATG | Reverse primer to clone AN400_RS26695 in pUT18 by NEBuilder HiFi DNA Assembly. Primer is used to amplify genes of interest |
| pGM119 | AGTCGACCCTGCAGCCCG | Forward primer to clone AN400_RS26690 in pT25 by NEBuilder HiFi DNA Assembly. Primer is used to linearise vector |
|  | CTAGAGGATCCCCGGGTAC | Reverse primer to clone AN400_RS26690 in pT25 by NEBuilder HiFi DNA Assembly. Primer is used to linearise vector |
|  | GGCGGGCTGCAGGGTCGACTATTGGATTGTCGTGGATAGTGATTAGC | Forward primer to clone AN400_RS26690 in pT25 by NEBuilder HiFi DNA Assembly. Primer is used to amplify gene of interest |
|  | GGTACCCGGGGATCCTCTAGTCACGCCTGCTGCTCAGC | Reverse primer to clone AN400_RS26690 in pT25 by NEBuilder HiFi DNA Assembly. Primer is used to amplify genes of interest |
| pGM120 | AGTCGACCCTGCAGCCCG | Forward primer to clone AN400_RS26695 in pT25 by NEBuilder HiFi DNA Assembly. Primer is used to linearise vector |
|  | CTAGAGGATCCCCGGGTAC | Reverse primer to clone AN400_RS26695 in pT25 by NEBuilder HiFi DNA Assembly. Primer is used to linearise vector |
|  | GGCGGGCTGCAGGGTCGACTAAAAAACCAGCAACG | Forward primer to clone AN400_RS26695 in pT25 by NEBuilder HiFi DNA Assembly. Primer is used to amplify gene of interest |
|  | GGTACCCGGGGATCCTCTAGCTAGTTGAGACTCGCCAAC | Reverse primer to clone AN400_RS26695 in pT25 by NEBuilder HiFi DNA Assembly. Primer is used to amplify genes of interest |
| pGM107 | AGTACTGCCAAGGAGCAAAAC | Forward primer to clone AN400_RS26690 with a C-termina His_6_ tag in pGM42 upstream of AN400_RS26695 by NEBuilder HiFi DNA Assembly. Primer is used to linearise vector |
|  | AGTACTGCCAAGGAGCAAAAC | Reverse primer to clone AN400_RS26690 with a C-termina His_6_ tag in pGM42 upstream of AN400_RS26695 by NEBuilder HiFi DNA Assembly. Primer is used to linearise vector |
|  | CGTTTTTTTGGGCTAGCGAATTCATGATTGGATTGTCGTGG | Forward primer to clone AN400_RS26690 with a C-termina His_6_ tag in pGM42 upstream of AN400_RS26695 by NEBuilder HiFi DNA Assembly. Primer is used to linearise vector |
|  | CTGTTTTGCTCCTTGGCAGTACTTTAGTGATGGTGATGGTG | Reverse primer to clone AN400_RS26690 with a C-termina His_6_ tag in pGM42 upstream of AN400_RS26695 by NEBuilder HiFi DNA Assembly. Primer is used to linearise vector |
| pGM116 | AGTACTGCCAAGGAGCAAAAC | Forward primer to clone AN400_RS26690 with a C-termina His_6_ tag in pBAD18 by NEBuilder HiFi DNA Assembly. Primer is used to linearise vector |
|  | AGTACTGCCAAGGAGCAAAAC | Forward primer to clone AN400_RS26690 with a C-termina His_6_ tag in pBAD18 by NEBuilder HiFi DNA Assembly. Primer is used to linearise vector |
|  | CCGTTTTTTTGGGCTAGCGAATTCGAATGATTGGATTGTCGTGG | Forward primer to clone AN400_RS26690 with a C-termina His_6_ tag in pBAD18 by NEBuilder HiFi DNA Assembly. Primer is used to linearise vector |
|  | AGGATCCCCGGGTACCGAGCTTAGTGATGGTGATGGTG | Forward primer to clone AN400_RS26690 with a C-termina His_6_ tag in pBAD18 by NEBuilder HiFi DNA Assembly. Primer is used to linearise vector |
| pGM197 | GGATCCACTAGTGAGCTC | Forward primer to clone AN400_RS26685, AN400_RS26690 and AN400_RS26695, including upstream and downstream promoter and terminator regions in pUC18-mini-Tn7T-Gm by NEBuilder HiFi DNA Assembly. Primer is used to linearise vector |
|  | CCCGGGCTGCAGGAATTC | Reverse primer to clone AN400_RS26690 and AN400_RS26695, including upstream and downstream promoter and terminator regions in pUC18-mini-Tn7T-Gm by NEBuilder HiFi DNA Assembly. Primer is used to linearise vector |
|  | atgagctcactagtggatccTTAGTGATAGAGCCAGGG | Forward primer to clone AN400_RS26685, AN400_RS26690 and AN400_RS26695, including upstream and downstream promoter and terminator regions in pUC18-mini-Tn7T-Gm by NEBuilder HiFi DNA Assembly. |
|  | aggaattcctgcagcccgggCTGACTGAACTTCTTACAAAC | Forward primer to clone AN400_RS26685, AN400_RS26690 and AN400_RS26695, including upstream and downstream promoter and terminator regions in pUC18-mini-Tn7T-Gm by NEBuilder HiFi DNA Assembly. |
| pGM198 | GGATCCACTAGTGAGCTC | Forward primer to clone AN400_RS26690 and AN400_RS26695, including upstream and downstream promoter and terminator regions in pUC18-mini-Tn7T-Gm by NEBuilder HiFi DNA Assembly. Primer is used to linearise vector |
|  | CCCGGGCTGCAGGAATTC | Reverse primer to clone AN400_RS26690 and AN400_RS26695, including upstream and downstream promoter and terminator regions in pUC18-mini-Tn7T-Gm by NEBuilder HiFi DNA Assembly. Primer is used to linearise vector |
|  | atgagctcactagtggatccGCCCCTCGATATGCGGGG | Forward primer to clone AN400_RS26690 and AN400_RS26695, including upstream and downstream promoter and terminator regions in pUC18-mini-Tn7T-Gm by NEBuilder HiFi DNA Assembly. |
|  | aggaattcctgcagcccgggCTGACTGAACTTCTTACAAACCAGGC | Forward primer to clone AN400_RS26690 and AN400_RS26695, including upstream and downstream promoter and terminator regions in pUC18-mini-Tn7T-Gm by NEBuilder HiFi DNA Assembly. |

* For the following plasmids the full length insert was synthetised by GenScript and the genes of interest were cloned in pBAD18 by NEBuilder HiFi DNA Assembly

| **Templates for cell-free *in vitro* protein synthesis** | **Sequence of relevant primers (5’-3’)**^a^ | **Description** |
| --- | --- | --- |
| ShdA II | GCGAATTAATACGACTCACTATAGGGCTTAAGTATAAGGAGGAAAAAATATGCTGCAGGATCGCTTTGCGGCCC | Forward primer to generate a DNA template for the *in vitro* synthesis of ShdA II (AN400_RS26690) from aminoacid 138 to 524 |
|  | AAA CCC CTC CGT TTA GAG AGG GGT TAT GCT AG TTA CACGCCTGCTGCTCAGCTGGCATC | Reverse primer to generate a DNA template for the *in vitro* synthesis of ShdA II (AN400_RS26690) from aminoacid 138 to 524 |
| ShdA I | GCGAATTAATACGACTCACTATAGGGCTTAAGTATAAGGAGGAAAAAAATGCTGCAGGATCGCTTCGCGGCCCTG | Forward primer to generate a DNA template for the *in vitro* synthesis of ShdA I (DL351_RS13220) from aminoacid 140 to 526 |
|  | AAACCCCTCCGTTTAGAGAGGGGTTATGCTATTACTGCGCCATTTCGCTGGACTC | Reverse primer to generate a DNA template for the *in vitro* synthesis of ShdA I (DL351_RS13220) from aminoacid 140 to 526 |
| ShdA III | GCGAATTAATACGACTCACTATAGGGCTTAAGTATAAGGAGGAAAAAAATGCAGGACAAATTCGCCAACCTGTC | Forward primer to generate a DNA template for the *in vitro* synthesis of ShdA III (EQ826_RS12590) from aminoacid 135 to 523 |
|  | AAACCCCTCCGTTTAGAGAGGGGTTATGCTAGTTATGCTTCCTCCTGCGCCTCGAAGGC | Reverse primer to generate a DNA template for the *in vitro* synthesis of ShdA III (EQ826_RS12590) from aminoacid 135 to 523 |
| ShdA IV | GCGAATTAATACGACTCACTATAGGGCTTAAGTATAAGGAGGAAAAAAATGCAGGATAAGTTCGCCAATCTGTCCAAG | Forward primer to generate a DNA template for the *in vitro* synthesis of ShdA IV (A9179_RS12860) from aminoacid 135 to 521 |
|  | AAACCCCTCCGTTTAGAGAGGGGTTATGCTAG TTACTGCGGCAATTCGCTGGGCTCATG | Reverse primer to generate a DNA template for the *in vitro* synthesis of ShdA IV (A9179_RS12860) from aminoacid 135 to 521 |
