# Supplementary material for: The novel anti-phage system Shield co-opts an RmuC domain to mediate phage defense across Pseudomonas species
Source: PLoS Genet. 2023 Jun 5;19(6):e1010784. doi: 10.1371/journal.pgen.1010784 (PMC10270631; doi:10.1371/journal.pgen.1010784)
Supplement: S1 Fig — FlaGs output representing the genomic neighbourhood of shdA genes belonging to different subtypes. The spectrum of genomes encoding these homologues is reported in S2–S5 Tables. FlaGs-grouped genes are numbered and coloured by FlaGs according to their association to a certain cluster. FlaGs-numbering of clustered genes is reported in S4 Table. Clustered genes were annotated as belonging to a specific antiphage system using PFAM and Defense-finder. These annotations are reported in S4 Table. shdA genes are coloured in blue and partner genes have a coloured outline as indicated on the figure. (PDF) [file pgen.1010784.s013.pdf]

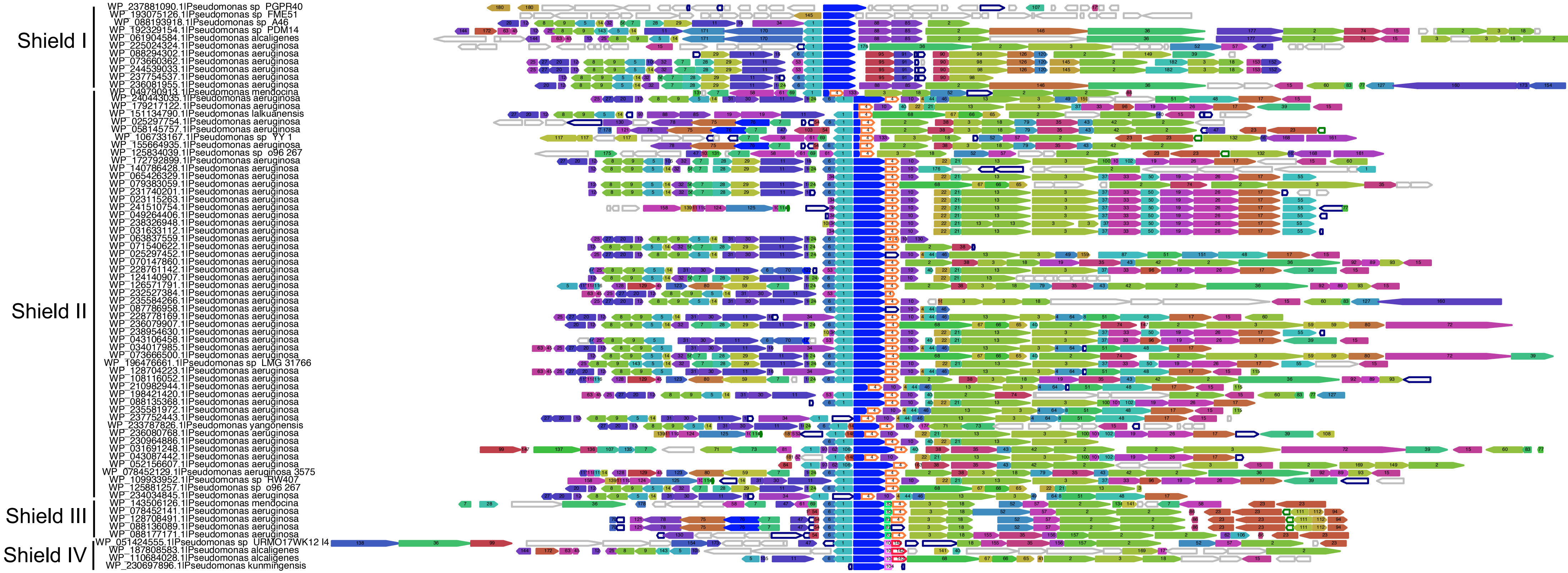
Shield I
Shield II
Shield III
Shield IV
WP_237881090.1|Pseudomonas sp PGPR40
WP_193075126.1|Pseudomonas sp FME51
WP_088193918.1|Pseudomonas sp A46
WP_192329154.1|Pseudomonas sp PDM14
WP_061904584.1|Pseudomonas alcaligenes
WP_225024324.1|Pseudomonas aeruginosa
WP_088294302.1|Pseudomonas aeruginosa
WP_073660362.1|Pseudomonas aeruginosa
WP_244539033.1|Pseudomonas aeruginosa
WP_237754537.1|Pseudomonas aeruginosa
WP_236081955.1|Pseudomonas aeruginosa
WP_049790913.1|Pseudomonas mendocina
WP_240443035.1|Pseudomonas aeruginosa
WP_179217122.1|Pseudomonas aeruginosa
WP_151134790.1|Pseudomonas lalkuanensis
WP_025297754.1|Pseudomonas aeruginosa
WP_058145757.1|Pseudomonas aeruginosa
WP_106733167.1|Pseudomonas sp YY 1
WP_155664935.1|Pseudomonas aeruginosa
WP_125834039.1|Pseudomonas sp o96 267
WP_172792899.1|Pseudomonas aeruginosa
WP_140786428.1|Pseudomonas aeruginosa
WP_065426329.1|Pseudomonas aeruginosa
WP_079383059.1|Pseudomonas aeruginosa
WP_231740201.1|Pseudomonas aeruginosa
WP_023115263.1|Pseudomonas aeruginosa
WP_241510754.1|Pseudomonas aeruginosa
WP_049264406.1|Pseudomonas aeruginosa
WP_238326948.1|Pseudomonas aeruginosa
WP_031633112.1|Pseudomonas aeruginosa
WP_063837559.1|Pseudomonas aeruginosa
WP_071540622.1|Pseudomonas aeruginosa
WP_025297452.1|Pseudomonas aeruginosa
WP_070147860.1|Pseudomonas aeruginosa
WP_228761142.1|Pseudomonas aeruginosa
WP_124140907.1|Pseudomonas aeruginosa
WP_126571791.1|Pseudomonas aeruginosa
WP_232527384.1|Pseudomonas aeruginosa
WP_235584266.1|Pseudomonas aeruginosa
WP_087786958.1|Pseudomonas aeruginosa
WP_228778169.1|Pseudomonas aeruginosa
WP_236079907.1|Pseudomonas aeruginosa
WP_238954630.1|Pseudomonas aeruginosa
WP_043106458.1|Pseudomonas aeruginosa
WP_034017985.1|Pseudomonas aeruginosa
WP_073666500.1|Pseudomonas aeruginosa
WP_196476661.1|Pseudomonas sp LMG 31766
WP_128704223.1|Pseudomonas aeruginosa
WP_108116052.1|Pseudomonas aeruginosa
WP_210982944.1|Pseudomonas aeruginosa
WP_198421420.1|Pseudomonas aeruginosa
WP_088135368.1|Pseudomonas aeruginosa
WP_235581972.1|Pseudomonas aeruginosa
WP_237752443.1|Pseudomonas aeruginosa
WP_233787826.1|Pseudomonas yangonensis
WP_236080768.1|Pseudomonas aeruginosa
WP_230964886.1|Pseudomonas aeruginosa
WP_031691248.1|Pseudomonas aeruginosa
WP_043087442.1|Pseudomonas aeruginosa
WP_052156607.1|Pseudomonas aeruginosa
WP_078452129.1|Pseudomonas aeruginosa 3575
WP_109933952.1|Pseudomonas sp RW407
WP_125881257.1|Pseudomonas sp o96 267
WP_234034845.1|Pseudomonas aeruginosa
WP_143506126.1|Pseudomonas mendocina
WP_078452141.1|Pseudomonas aeruginosa
WP_128708491.1|Pseudomonas aeruginosa
WP_088136089.1|Pseudomonas aeruginosa
WP_088177171.1|Pseudomonas aeruginosa
WP_051424555.1|Pseudomonas sp URMO17WK12 I4
WP_187808583.1|Pseudomonas alcaligenes
WP_110684028.1|Pseudomonas alcaligenes
WP_230697896.1|Pseudomonas kunmingensis

Figure S1. Definition of Shield subtypes. FlaGs output representing the genomic neighbourhood of *shdA* genes belonging to different subtypes. The spectrum of genomes encoding these homologues is reported in Tables S2-S5. FlaGs-grouped genes are numbered and coloured by FlaGs according to their association to a certain cluster. FlaGs-numbering of clustered genes is reported in Table S4. Clustered genes were annotated as belonging to a specific antiphage system using PFAM and defense-finder. These annotations are reported in Table S4. *shdA* genes are coloured in blue and partner genes have a coloured outline as indicated on the figure.
