# Supplementary material for: The novel anti-phage system Shield co-opts an RmuC domain to mediate phage defense across Pseudomonas species
Source: PLoS Genet. 2023 Jun 5;19(6):e1010784. doi: 10.1371/journal.pgen.1010784 (PMC10270631; doi:10.1371/journal.pgen.1010784)
Supplement: S2 Fig — Representative alignments of ShdA homologues across Shield subtypes. One representative ShdA homologue was chosen for each Shield subtype and aligned using MUSCLE. A full alignment, involving all homologues, is shown in S2 Fig. (PDF) [file pgen.1010784.s014.pdf]

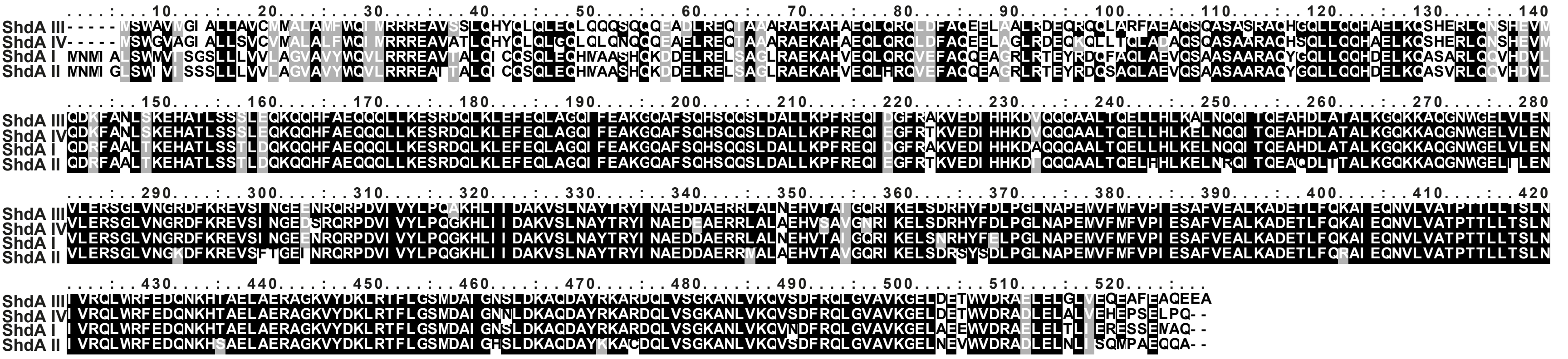
ShdA III
ShdA IV
ShdA I
ShdA II

Figure S2 Alignment of one representative ShdA homologue from each Shield subtype. Representative alignments of ShdA homologues across Shield subtypes. One representative ShdA homologue was chosen for each Shield subtype and aligned using MUSCLE. A full alignment, involving all homologues, is shown in Figure S2
