# Supplementary material for: The novel anti-phage system Shield co-opts an RmuC domain to mediate phage defense across Pseudomonas species
Source: PLoS Genet. 2023 Jun 5;19(6):e1010784. doi: 10.1371/journal.pgen.1010784 (PMC10270631; doi:10.1371/journal.pgen.1010784)
Supplement: S3 Fig — Alignment of ShdA homologues using MUSCLE. The alignment was visualised in Boxshade and coloured by percentage of identity. (PDF) [file pgen.1010784.s015.pdf]

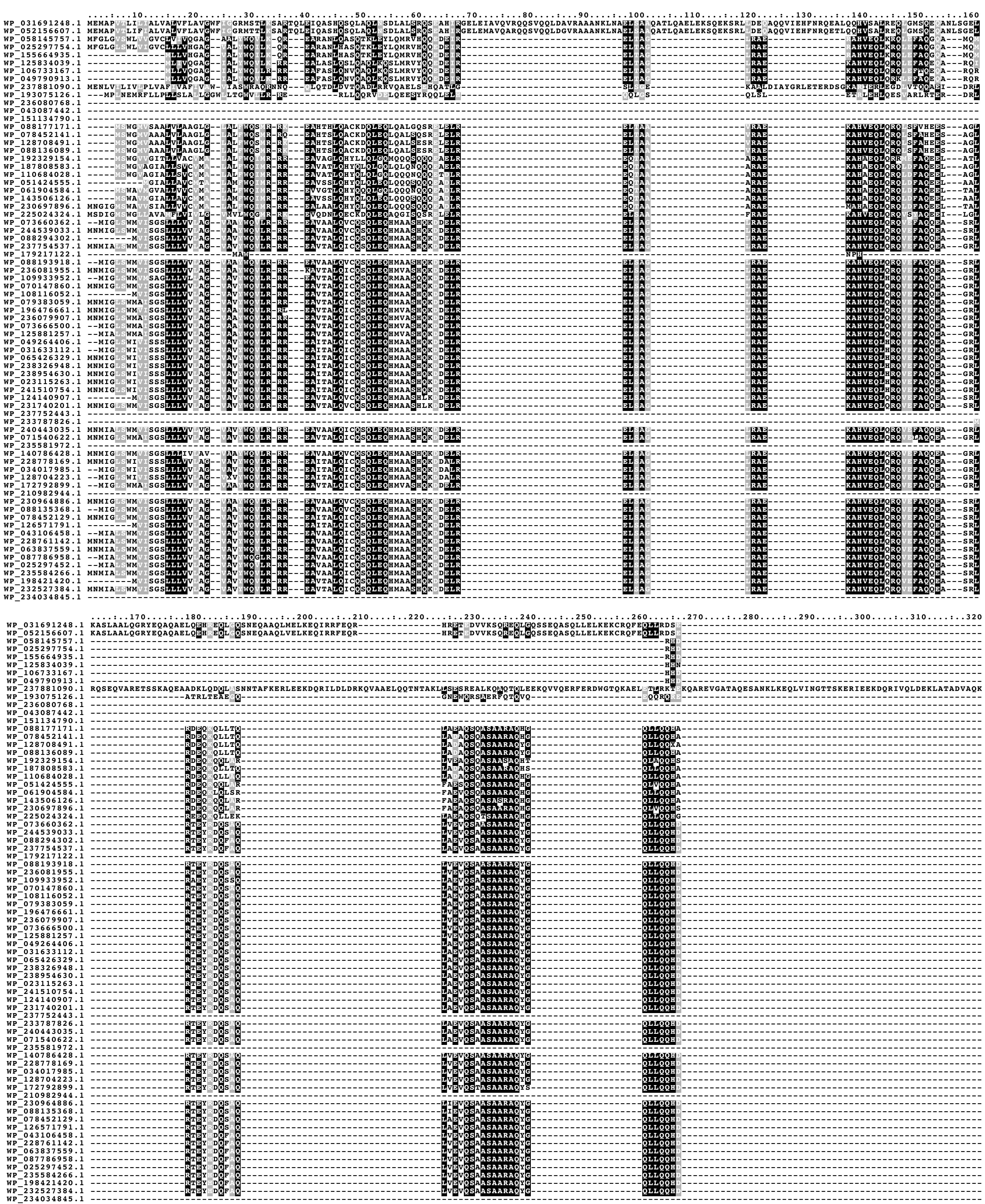

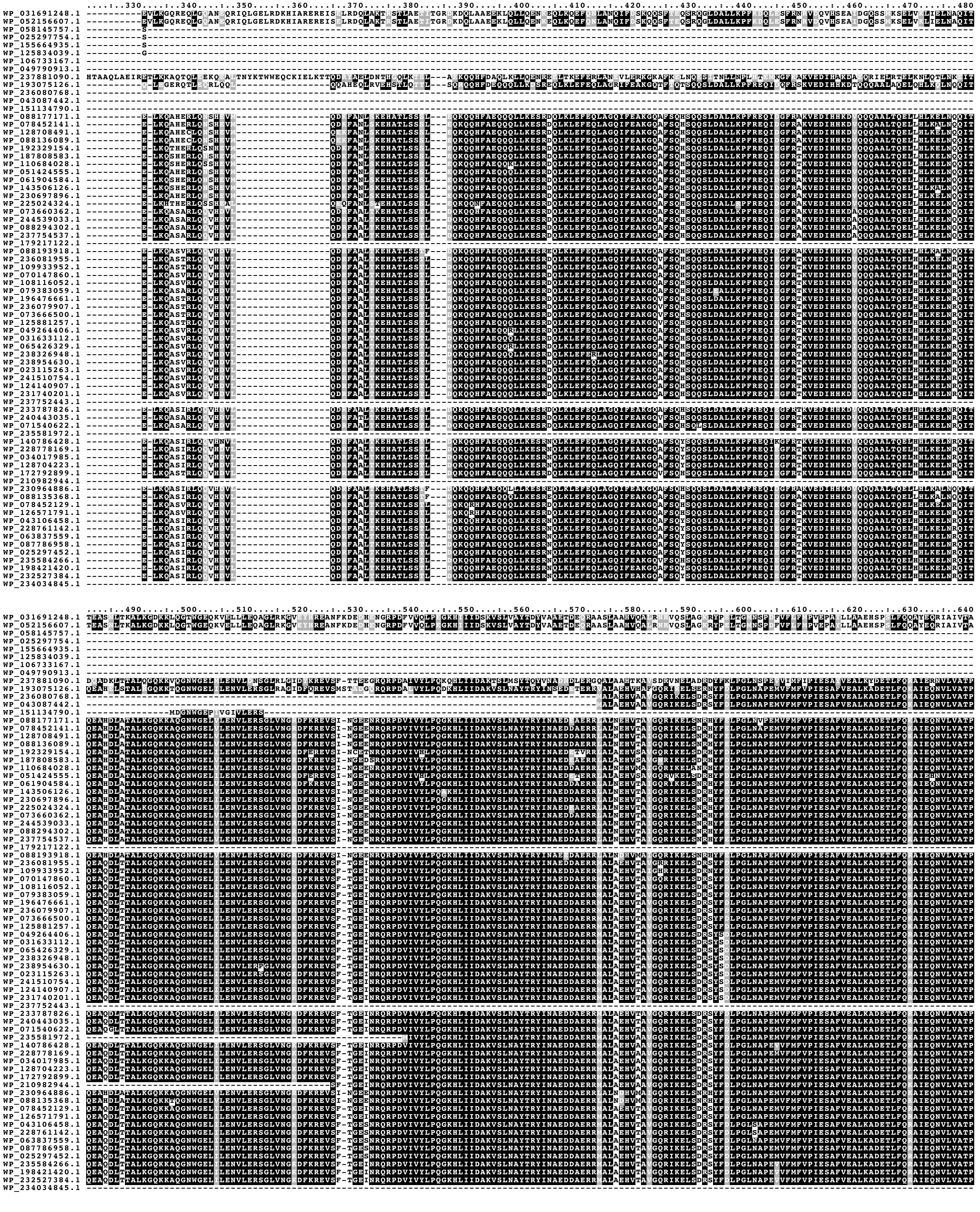

```
                ....:..650....:..660....:..670....:..680....:..690....:..700....:..710....:..720....:..730....:..740....:..750....:..760...
WP_031691248.1  TTLLPVLRVVANLWSIQRQNQSTLQLAQQASSVYNKLRVFVEKMEK--LGNQIGTVQKTYADTFNTLTGPHGSLTRTVDKFVDLGVKVVKRLPASVVGAGDDPAGEGDVLAMEETTE------
WP_052156607.1  TTLLPVLRVVANLWSIQRQNQSTLQLAQQASSVYNKLRVFVEKMEK--LGNQIGTVQKTYADTFNTLTGPHGSLTRTVDKFVDLGVKVVKRLPASVVGAGDDPAGEGDVLAMEETTE------
WP_058145757.1  ---------------------------------------------------------------------------------------------------------------------------
WP_025297754.1  ---------------------------------------------------------------------------------------------------------------------------
WP_155664935.1  ---------------------------------------------------------------------------------------------------------------------------
WP_125834039.1  ---------------------------------------------------------------------------------------------------------------------------
WP_106733167.1  ---------------------------------------------------------------C-----------------------------------------------------------
WP_049790913.1  ---------------------------------------------------------------C-----------------------------------------------------------
WP_237881090.1  TTLLTSLNIVRQLWRFEDQNKHTAELANRAEKFYNKLNGFLTSMQG--VGNQLNRARESYDKAFGQLYIGKGNLIKQAAEFKDLGVSVQKELPVDLVERANLELSSAVAELAEIEATVLIASS
WP_193075126.1  TTLLTSLNIVRQLWRFEDQNRHTAELAERAARIHDKLRTFLGSMDQ--IEKGLDRAGEAYRRARDQLINGRGNLVKQVQDFKELGVSVKGELGEHWTDRAELEL---GHDASADTL-------
WP_236080768.1  TTLLTSLNIVRQLWRFENQNKHTAELAERAGKVYDKLRAAWTPSATAWTRRRMPTRKPTISWSVARLTWSSKSVISVSWAWR---------LKEVWVDRADLELNLISQMPAEQQA-------
WP_043087442.1  TTLLTSLNIVRQLWRFEDQNKHTAELAERAGKVYDKLRAAWTPSATAWTRRRMPTRKPTISWSVARLTWSSKSVISVSWAWR---------LKEVWVDRADLELNLISQMPAEQQA-------
WP_151134790.1  ---------------------------------------------------------------C--LVNGKAILVKQVSDFRQLGVSVKGELNEVWLDRADLELNLISQVPAEEQA-------
WP_088177171.1  TTLLTSLNIVRQLWRFEDQNKHTAELAERAGKVYDKLRTFLGSMDA--IGNSLDKAQDAYRKACDQLLSGKANLVKQVSDFRQLGVAVKGELDETWVDRAELELGLVEQEAFEAQEET-----
WP_078452141.1  TTLLTSLNIVRQLWRFEDQNKHTAELAERAGKVYDKLRTFLGSMDA--IGNSLDKAQDAYRKARDQLVSGKANLVKQVSDFRQLGVAVKGELDETWVDRAELELGLVEQEAFEVQEQA-----
WP_128708491.1  TTLLTSLNIVRQLWRFEDQNKHTAELAERAGKVYDKLRTFLGSMDA--IGNSLDKAQDAYRKARDQLVSGKANLVKQVSDFRQLGVAVKGELDETWVDRAELELGLVEQEAFEAQEQA-----
WP_088136089.1  TTLLTSLNIVRQLWRFEDQNKHTAELAERAGKVYDKLRTFLGSMDA--IGNSLDKAQDAYRKARDQLVSGKANLVKQVSDFRQLGVAVKGELDETWVDRAELELGLVEQEAFEAQEQA-----
WP_192329154.1  TTLLTSLNIVRQLWRFEDQNKHTAELAERAGKVYDKLRTFLGSMDA--IGNSLDKAQDAYRKARDQLVSGKANLVKQVNDFRQLGVAVKGELAGEWVDRADLELALVGHEPNEILQ-------
WP_187808583.1  TTLLTSLNIVRQLWRFEDQNKHTAELAERAGKVYDKLRTFLGSMDA--IGNNLDKAQDAYRKARDQLVSGKANLVKQVSDFRQLGVAVKGELDETWVDRADLELALVEHEPSELPQ-------
WP_110684028.1  TTLLTSLNIVRQLWRFEDQNKHTAELAERAGKVYDKLRTFLGSMDA--IGNSLDKAQDAYRKARDQLVSGKANLVKQVNDFRQLGVAVKGELDETWVDRADLELALVEHEPSESAQ-------
WP_051424555.1  TTLLTSLNIVRQLWRFEDQNKHTAELAERAGKVYDKLRTFLGSMDA--IGNSLDKAQDAYRKARDQLVSGKANLVKQVSDFRQLGVAVKGELDETWVDRADLELTLVGHKTSEMAQ-------
WP_061904584.1  TTLLTSLNIVRQLWRFEDQNKHTAELAERAGKVYDKMRTFLGSMDA--IGNSLDKAQDAYRKARDQLVSGKANLVKQVNDFRQLGVAVKGELAEEWVDRADLELVLVGHEPNEILQ-------
WP_143506126.1  TTLLTSLNIVRQLWRFEDQNKHTAELAERAGKVYDKLRTFLGSMDA--IGNSLDKAQDAYRKARDQLVSGKANLVKQVSDFRQLGVAVKGELDETWVDRAELELGLVEQEAFEAQEEA-----
WP_230697896.1  TTLLTSLNIVRQLWRFEDQNKHTAELAERAGKVYDKLRTFLGSMDA--IGNSLDKAQDAYRKARDQLVSGKANLVKQVSDFRQLGVAVKGELDETWVDRADLELALVEREPSELAQ-------
WP_225024324.1  TTLLTSLNIVRQLWRFEDQNKHTAELAERAGKVYDKLRTFLGSMDA--IGNSLDKAQDAYRKARDQLVSGKGNLVKQVSDFRQLGVAVKGELDESWVDRADLELSLIEQGSLETQE-------
WP_073660362.1  TTLLTSLNIVRQLWRFEDQNKHTAELAERAGKVYDKLRTFLGSMDA--IGNSLDKAQDAYRKARDQLVSGKANLVKQVNDFRQLGVAVKGELAEEWVDRAELELTLIERESSEMAQ-------
WP_244539033.1  TTLLTSLNIVRQLWRFEDQNKHTAELAERAGKVYDKLRTFLGSMDA--IGNSLDKAQDAYRKARDQLVSGKANLVKQVNDFRQLGVAVKGELAEEWVDRAELELTLIERESSEMAQ-------
WP_088294302.1  TTLLTSLNIVRQLWRFEDQNKHTAELAERAGKVYDKLRTFLGSMDA--IGNSLDKAQDAYRKARDQLVSGKANLVKQVNDFRQLGVAVKGELAEEWVDRAELELTLIERESSEMAQ-------
WP_237754537.1  TTLLTSLNIVRQLWRFEDQNKHTAELAERAGKVYDKLRTFLGSMDA--IGNSLDKAQDAYRKARDQLVSGKANLVKQVNDFRQLGVAVKGELAEEWVDRAELELTLIERESSEMAQ-------
WP_179217122.1  --------------------------------------------------HSLDKAQDAYKKACDQLVSGKANLVKQVSDFRQLGVAVKGELNEVWVDRADLELNLISQMPAEQQA-------
WP_088193918.1  TTLLTSLNIVRQLWRFEDQNKHTAELAERAGKVYDKLRTFLGSMDV--IGHSLDKAQDAYKKACDQLVNGKANLVKQVSDFRQLGVAVKGELAEEWVERADLELALAGHEPNEILQ-------
WP_236081955.1  TTLLTSLNIVRQLWRFEDQNKHTAELAERAGKVYDKLRTFLSSMDA--IGHSLDKAQDAYKKACDQLVSGKANLVKQVSDFRQLGVAVKGELAEEWVERADLELALAGHEPNETLQ-------
WP_109933952.1  TTLLTSLNIVRQLWRFEDQNKHTAELAERAGKVYDKLRTFLGSMDA--IGHSLDKAQDAYKKACDQLVNGKANLVKQVSDFRQLGVAVKGELNEVWVDRADLELNLLSQMPAEQQA-------
WP_070147860.1  TTLLTSLNIVRQLWRFEDQNKHTAELAERAGKVYDKLRTFLGSMDA--IGHSLDKAQDAYKKACDQLVNGKANLVKQVSDFRQLGVAVKGELSEVWGDRADLELNLIPQMPAEQSM-------
WP_108116052.1  TTLLTSLNIVRQLWRFEDQNKHTAELAERAGKVYDKLRTFLGSMDA--IGHSLDKAQDAYKKACDQLVNGKANLVKQVSDFRQLGVAVKGELSEVWGDRADLELNLIPQMPAEQSM-------
WP_079383059.1  TTLLTSLNIVRQLWRFEDQNKHTAELAERAGKVYDKLRTFLSSMDA--IGHSLDRAQDAYKKACDQLVSGKANLVKQVSDFRQLGVAVKGELNEVWVDRADLELNLISQMPAEQQA-------
WP_196476661.1  TTLLTSLNIVRQLWRFEDQNKHTAELAERAGKVYDKLRTFLSSMDA--IGHSLDRAQDAYKKACDQLVSGKANLVKQVSDFRQLGVAVKGELNEVWVDRADLELNLISQMPAEQQA-------
WP_236079907.1  TTLLTSLNIVRQLWRFEDQNKHTAELAERAGKVYDKLRTFLSSMDA--IGHSLDRAQDAYKKACDQLVSGKANLVKQVSDFRQLGVAVKGELNEVWVDRADLELNLISQMPAEQQA-------
WP_073666500.1  TTLLTSLNIVRQLWRFEDQNKHTAELAERAGKVYDKLRTFLSSMDA--IGHSLDRAQDAYKKACDQLVSGKANLVKQVSDFRQLGVAVKGELNEVWVDRADLELNLISQMPAEQQA-------
WP_125881257.1  TTLLTSLNIVRQLWRFEDQNKHTAELAERAGKVYDKLRTFLSSMDA--IGHSLDRAQDAYKKACDQLVSGKANLVKQVSDFRQLGVAVKGELNEVWVDRADLELNLISQMPAEQQA-------
WP_049264406.1  TTLLTSLNIVRQLWRFEDQNKHSAELAERAGKVYDKLRTFLGSMDA--IGHSLDKAQDAYKKACDQLVSGKANLVKQVSDFRQLGVAVKGELNEVWVDRADLELNLISQMPAEQQA-------
WP_031633112.1  TTLLTSLNIVRQLWRFEDQNKHSAELAERAGKVYDKLRTFLGSMDA--IGHSLDKAQDAYKKACDQLVSGKANLVKQVSDFRQLGVAVKGELNEVWVDRADLELNLISQMPAEQQA-------
WP_065426329.1  TTLLTSLNIVRQLWRFEDQNKHSAELAERAGKVYDKLRTFLGSMDA--IGHSLDKAQDAYKKACDQLVSGKANLVKQVSDFRQLGVAVKGELNEVWVDRADLELNLISQMPAEQQA-------
WP_238326948.1  TTLLTSLNIVRQLWRFEDQNKHSAELAERAGKVYDKLRTFLGSMDA--IGHSLDKAQDAYKKACDQLVSGKANLVKQVSDFRQLGVAVKGELNEVWVDRADLELNLISQMPAEQQA-------
WP_238954630.1  TTLLTSLNIVRQLWRFEDQNKHSAELAERAGKVYDKLRTFLGSMDA--IGHSLDKAQDAYKKACDQLVSGKANLVKQVSDFRQLGVAVKGELNEVWVDRADLELNLISQMPAEQQA-------
WP_023115263.1  TTLLTSLNIVRQLWRFEDQNKHSAELAERAGKVYDKLRTFLGSMDA--IGHSLDKAQDAYKKACDQLVSGKANLVKQVSDFRQLGVAVKGELNEVWVDRADLELNLISQMPAEQQA-------
WP_241510754.1  TTLLTSLNIVRQLWRFEDQNKHSAELAERAGKVYDKLRTFLGSMDA--IGRSLDKAQDAYKKACDQLVSGKANLVKQVSDFRQLGVAVKGELNEVWVDRADLELNLISQMPAEQQA-------
WP_124140907.1  TTLLTSLNIVRQLWRFEDQNKHSAELAERAGKVYDKLRTFLGSMDA--IGHSLDKAQDAYKKACDQLVSGKANLVKQVSDFRQLGVAVKGELNEVWVDRADLELNLISQMPAEQQA-------
WP_231740201.1  TTLLTSLNIVRQLWRFEDQNKHSAELAERAGKVYDKLRTFLGSMDA--IGHSLDKAQDAYKKACDQLVSGKANLVKQVSDFRQLGVAVKGELNEVWVDRADLELNLISQMPAEQQA-------
WP_237752443.1  ---------MRQLWRFEDQNKHTAELAERAGKVYDKLRTFLGSMDA--IGHSLDKAQDAYKKACDQLVSGKANLVKQVSDFRQLGVAVKGELNEVWVDRADLELNLISQMPAEQQA-------
WP_233787826.1  TTLLTSLNIVRQLWRFEDQNKHTAELAERAGKVYDKLRTFLGSMDA--IGHSLDKAQDAYKKACDQLVSGKANLVKQVSDFRQLGVAVKGELSEVWVDRADLELNLISQMPAEQQA-------
WP_240443035.1  TTLLTSLNIVRQLWRFEDQNKHTAELAERAGKVYDKLRTFLGSMDA--IGHSLDKAQDAYKKACDQLVSGKANLVKQVSDFRQLGVAVKGELNEVWVDRADLELNLISQMPAEQQA-------
WP_071540622.1  TTLLTSLNIVRQLWRFEDQNKHTAELAERAGKVYDKLRTFLGSMDA--IGHSLDKAQDAYKKACDQLVSGKANLVKQVSDFRQLGVAVKGELNEVWVDRADLELNLISQMPAEQQA-------
WP_235581972.1  TTLLTSLNIVRQLWRFEDQNKHTAELAERAGKVYDKLRTFLGSMDA--IGHSLDKAQDAYKKACDQLVSGKANLVKQVSDFRQLGVAVKGELNEVWVDRADLELNLISQMPAEQQA-------
WP_140786428.1  TTLLTSLNIVRQLWRFEDQNKHTAELAERAGKVYDKLRTFLGSMDA--IGHSLDKAQDAYKKACDQLVSGKANLVKQVSDFRQLGVAVKGELNEVWVDRADLELNLISQMPAEQQA-------
WP_228778169.1  TTLLTSLNIVRQLWRFEDQNKHTAELAERAGKVYDKLRTFLGSMDA--IGHSLDKAQDAYKKACDQLVSGKANLVKQVSDFRQLGVAVKGELNEVWVDRADLELNLISQMPAEQQA-------
WP_034017985.1  TTLLTSLNIVRQLWRFEDQNKHTAELAERAGKVYDKLRTFLGSMDA--IGHSLDKAQDAYKKACDQLVSGKANLVKQVSDFRQLGVAVKGELNEVWVDRADLELNLISQMPAEQQA-------
WP_128704223.1  TTLLTSLNIVRQLWRFEDQNKHTAELAERAGKVYDKLRTFLGSMDA--IGHSLDKAQDAYKKACDQLVSGKANLVKQVSDFRQLGVAVKGELNEVWVDRADLELNLISQMPAEQQA-------
WP_172792899.1  TTLLTSLNIVRQLWRFEDQNKHTAELAERAGKVYDKLRTFLGSMDA--IGHSLDKAQDAYKKACDQLVSGKANLVKQVSDFRQLGVAVKGELNEVWVDRADLELNLISQMPAEQQA-------
WP_210982944.1  TTLLTSLNIVRQLWRFEDQNKHTAELAERAGKVYDKLRTFLGSMDA--IGHSLDKAQDAYKKACDQLVSGKANLVKQVSDFRQLGVAVKGELNEVWVDRADLELNLISQMPAEQQA-------
WP_230964886.1  TTLLTSLNIVRQLWRFEDQNKHTAELAERAGKVYDKLRTFLGSMDA--IGHSLDKAQDAYKKACDQLVSGKANLVKQVSDFRQLGVAVKGELNEVWVDRADLELNLISQMPAEQQA-------
WP_088135368.1  TTLLTSLNIVRQLWRFEDQNKHTAELAERAGKVYDKLRTFLGSMDA--IGHSLDKAQDAYKKACDQLVSGKANLVKQVSDFRQLGVAVKGELNEVWVDRADLELNLISQMPAEQQA-------
WP_078452129.1  TTLLTSLNIVRQLWRFEDQNKHTAELAERAGKVYDKLRTFLGSMDA--IGHSLDKAQDAYKKACDQLVSGKANLVKQVSDFRQLGVAVKGELNEVWVDRADLELNLISQMPAEQQA-------
WP_126571791.1  TTLLTSLNIVRQLWRFEDQNKHTAELAERAGKVYDKLRTFLGSMDA--IGHSLDKAQDAYKKACDQLVSGKANLVKQVSDFRQLGVAVKGELNEVWVDRADLELNLISQMPAEQQA-------
WP_043106458.1  TTLLTSLNIVRQLWRFEDQNKHTAELAERAGKVYDKLRTFLGSMDA--IGHSLDKAQDAYKKACDQLVSGKANLVKQVSDFRQLGVAVKGELNEVWVDRADLELNLISQMPAEQQA-------
WP_228761142.1  TTLLTSLNIVRQLWRFEDQNKHTAELAERAGKVYDKLRTFLGSMDA--IGHSLDKAQDAYKKACDQLVSGKANLVKQVSDFRQLGVAVKGELNEVWVDRADLELNLISQMPAEQQA-------
WP_063837559.1  TTLLTSLNIVRQLWRFEDQNKHTAELAERAGKVYDKLRTFLGSMDA--IGHSLDKAQDAYKKACDQLVSGKANLVKQVSDFRQLGVAVKGELNEVWVDRADLELNLISQMPAEQQA-------
WP_087786958.1  TTLLTSLNIVRQLWRFEDQNKHTAELAERAGKVYDKLRTFLGSMDA--IGHSLDKAQDAYKKACDQLVSGKANLVKQVSDFRQLGVAVKGELNEVWVDRADLELNLISQMPAEQQA-------
WP_025297452.1  TTLLTSLNIVRQLWRFEDQNKHTAELAERAGKVYDKLRTFLGSMDA--IGHSLDKAQDAYKKACDQLVSGKANLVKQVSDFRQLGVAVKGELNEVWVDRADLELNLISQMPAEQQA-------
WP_235584266.1  TTLLTSLNIVRQLWRFEDQNKHTAELAERAGKVYDKLRTFLGSMDA--IGHSLDKAQDAYKKACDQLVSGKANLVKQVSDFRQLGVAVKGELNEVWVDRADLELNLISQMPAEQQA-------
WP_198421420.1  TTLLTSLNIVRQLWRFEDQNKHTAELAERAGKVYDKLRTFLGSMDA--IGHSLDKAQDAYKKACDQLVSGKANLVKQVSDFRQLGVAVKGELNEVWVDRADLELNLISQMPAEQQA-------
WP_232527384.1  TTLLTSLNIVRQLWRFEDQNKHTAELAERAGKVYDKLRTFLGSMDA--IGHSLDKAQDAYKKACDQLVSGKANLVKQVSDFRQLGVAVKGELNEVWVDRADLELNLISQMPAEQQA-------
WP_234034845.1  --MLTSLNIVRQLWRFEDQNKHTAELAERAGKVYDKLRTFLGSMDA--IGHSLDKAQDAYKKACDQLVSGKANLVKQVSDFRQLGVAVKGELNEVWVDRADLELNLISQMPAEQQA-------
```

Figure S3. Alignment of ShdA homologues from different subtypes. Alignment of ShdA homologues using MUSCLE. The alignment was visualised in Boxshade and coloured by percentage of identity.
