# Supplementary material for: The novel anti-phage system Shield co-opts an RmuC domain to mediate phage defense across Pseudomonas species
Source: PLoS Genet. 2023 Jun 5;19(6):e1010784. doi: 10.1371/journal.pgen.1010784 (PMC10270631; doi:10.1371/journal.pgen.1010784)
Supplement: S4 Fig — (a) Schematic representation of ShdA homologues encoded within DISARM operons. The full set of DISARM-associated ShdA loci is shown in S4 Fig. (b) Phylogenetic tree based on the ShdA homologues from S1 Fig in addition to DISARM-associated ShdA. Coloured blocks were used to show ShdA homologues belonging to distinct Shield subtypes or DISARM-like loci. (PDF) [file pgen.1010784.s016.pdf]

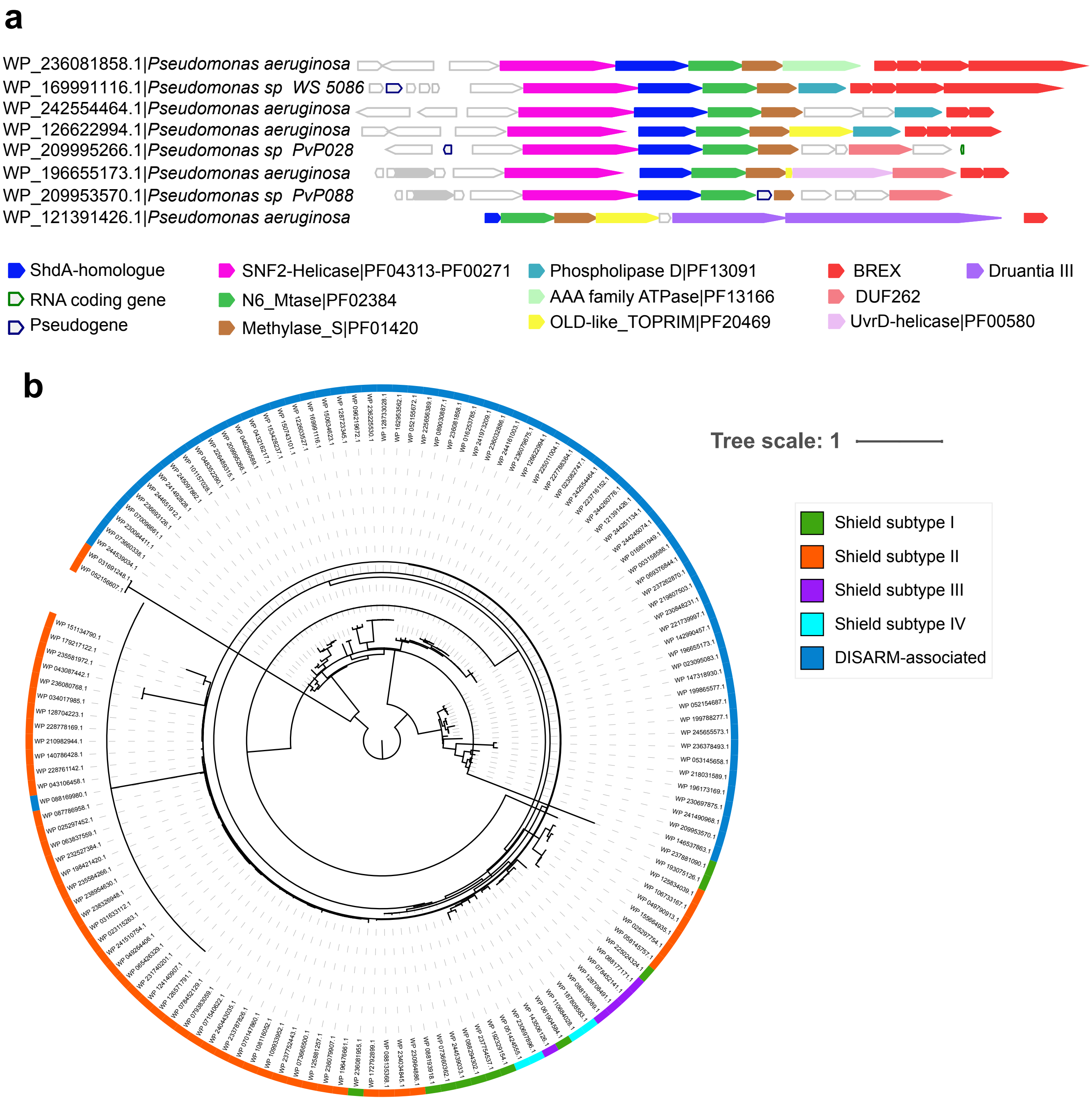
a
WP_236081858.1|Pseudomonas aeruginosa
WP_169991116.1|Pseudomonas sp WS 5086
WP_242554464.1|Pseudomonas aeruginosa
WP_126622994.1|Pseudomonas aeruginosa
WP_209995266.1|Pseudomonas sp PvP028
WP_196655173.1|Pseudomonas aeruginosa
WP_209953570.1|Pseudomonas sp PvP088
WP_121391426.1|Pseudomonas aeruginosa
ShdA-homologue
RNA coding gene
Pseudogene
SNF2-Helicase|PF04313-PF00271
N6_Mtase|PF02384
Methylase_S|PF01420
Phospholipase D|PF13091
AAA family ATPase|PF13166
OLD-like_TOPRIM|PF20469
BREX
DUF262
UvrD-helicase|PF00580
Druantia III
b
Tree scale: 1
Shield subtype I
Shield subtype II
Shield subtype III
Shield subtype IV
DISARM-associated

Figure S4: ShdA homologues are encoded in DISARM loci. (a) Schematic representation of ShdA homologues encoded within DISARM operons. The full set of DISARM-associated ShdA loci is shown in Figure S4. **(b)** Phylogenetic tree based on the ShdA homologues from Figure S1 in addition to DISARM-associated ShdA. Coloured blocks were used to show ShdA homologues belonging to distinct Shield subtypes or DISARM-like loci.
