# Supplementary material for: The novel anti-phage system Shield co-opts an RmuC domain to mediate phage defense across Pseudomonas species
Source: PLoS Genet. 2023 Jun 5;19(6):e1010784. doi: 10.1371/journal.pgen.1010784 (PMC10270631; doi:10.1371/journal.pgen.1010784)
Supplement: S5 Fig — FlaGs output representing the genomic neighbourhood of distant ShdA homologues shows that these can be encoded within DISARM-like operons. Neighbouring genes are clustered together based on similarity and each cluster is numbered. Definition of neighbouring gene clusters is reported in S8 Table. (PDF) [file pgen.1010784.s017.pdf]

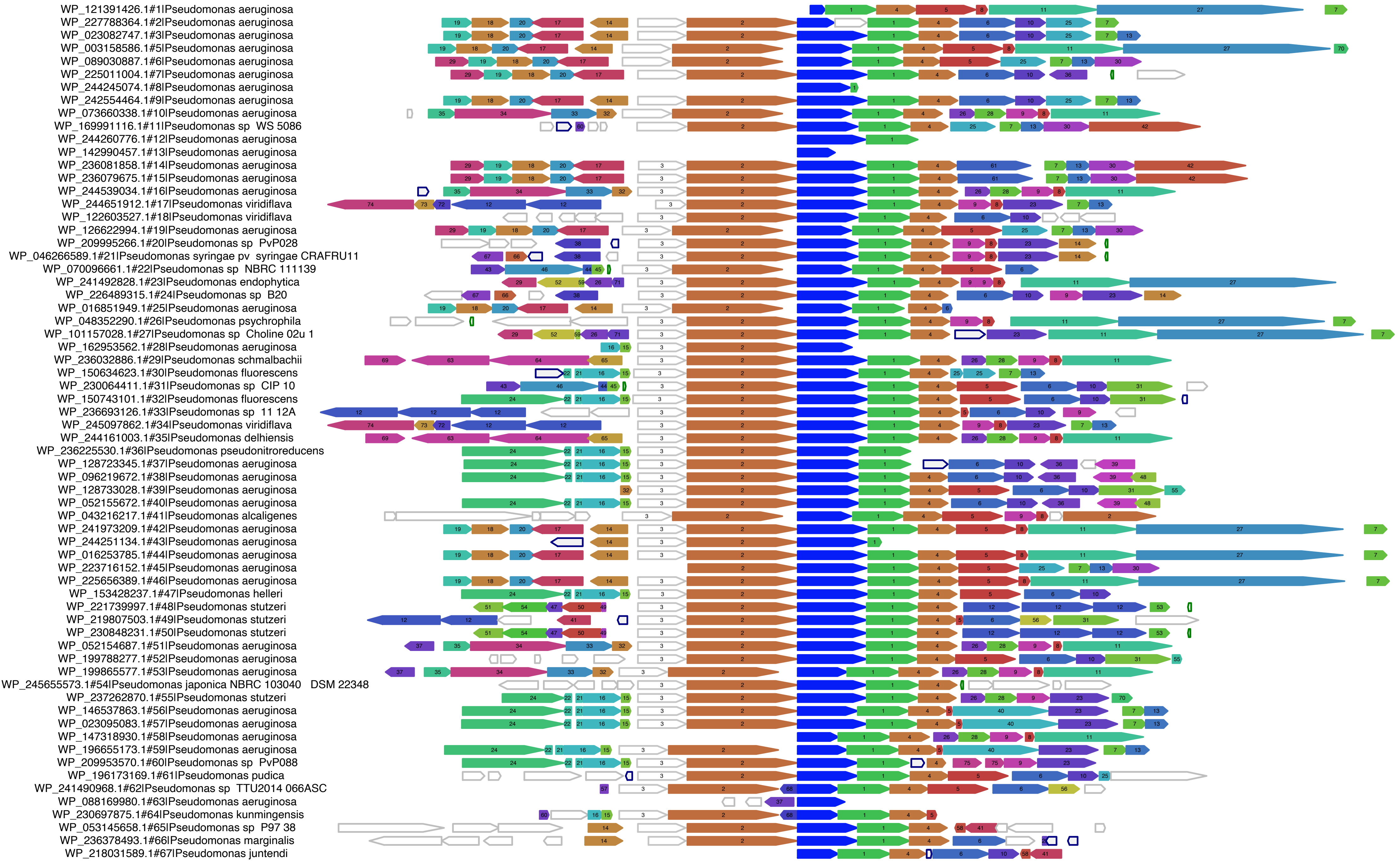
WP_121391426.1#1|Pseudomonas aeruginosa
WP_227788364.1#2|Pseudomonas aeruginosa
WP_023082747.1#3|Pseudomonas aeruginosa
WP_003158586.1#5|Pseudomonas aeruginosa
WP_089030887.1#6|Pseudomonas aeruginosa
WP_225011004.1#7|Pseudomonas aeruginosa
WP_244245074.1#8|Pseudomonas aeruginosa
WP_242554464.1#9|Pseudomonas aeruginosa
WP_073660338.1#10|Pseudomonas aeruginosa
WP_169991116.1#11|Pseudomonas sp WS 5086
WP_244260776.1#12|Pseudomonas aeruginosa
WP_142990457.1#13|Pseudomonas aeruginosa
WP_236081858.1#14|Pseudomonas aeruginosa
WP_236079675.1#15|Pseudomonas aeruginosa
WP_244539034.1#16|Pseudomonas aeruginosa
WP_244651912.1#17|Pseudomonas viridiflava
WP_122603527.1#18|Pseudomonas viridiflava
WP_126622994.1#19|Pseudomonas aeruginosa
WP_209995266.1#20|Pseudomonas sp PvP028
WP_046266589.1#21|Pseudomonas syringae pv syringae CRAFRU11
WP_070096661.1#22|Pseudomonas sp NBRC 111139
WP_241492828.1#23|Pseudomonas endophytica
WP_226489315.1#24|Pseudomonas sp B20
WP_016851949.1#25|Pseudomonas aeruginosa
WP_048352290.1#26|Pseudomonas psychrophila
WP_101157028.1#27|Pseudomonas sp Choline 02u 1
WP_162953562.1#28|Pseudomonas aeruginosa
WP_236032886.1#29|Pseudomonas schmalbachii
WP_150634623.1#30|Pseudomonas fluorescens
WP_230064411.1#31|Pseudomonas sp CIP 10
WP_150743101.1#32|Pseudomonas fluorescens
WP_236693126.1#33|Pseudomonas sp 11 12A
WP_245097862.1#34|Pseudomonas viridiflava
WP_244161003.1#35|Pseudomonas delhiensis
WP_236225530.1#36|Pseudomonas pseudonitroreducens
WP_128723345.1#37|Pseudomonas aeruginosa
WP_096219672.1#38|Pseudomonas aeruginosa
WP_128733028.1#39|Pseudomonas aeruginosa
WP_052155672.1#40|Pseudomonas aeruginosa
WP_043216217.1#41|Pseudomonas alcaligenes
WP_241973209.1#42|Pseudomonas aeruginosa
WP_244251134.1#43|Pseudomonas aeruginosa
WP_016253785.1#44|Pseudomonas aeruginosa
WP_223716152.1#45|Pseudomonas aeruginosa
WP_225656389.1#46|Pseudomonas aeruginosa
WP_153428237.1#47|Pseudomonas helleri
WP_221739997.1#48|Pseudomonas stutzeri
WP_219807503.1#49|Pseudomonas stutzeri
WP_230848231.1#50|Pseudomonas stutzeri
WP_052154687.1#51|Pseudomonas aeruginosa
WP_199788277.1#52|Pseudomonas aeruginosa
WP_199865577.1#53|Pseudomonas aeruginosa
WP_245655573.1#54|Pseudomonas japonica NBRC 103040 DSM 22348
WP_237262870.1#55|Pseudomonas stutzeri
WP_146537863.1#56|Pseudomonas aeruginosa
WP_023095083.1#57|Pseudomonas aeruginosa
WP_147318930.1#58|Pseudomonas aeruginosa
WP_196655173.1#59|Pseudomonas aeruginosa
WP_209953570.1#60|Pseudomonas sp PvP088
WP_196173169.1#61|Pseudomonas pudica
WP_241490968.1#62|Pseudomonas sp TTU2014 066ASC
WP_088169980.1#63|Pseudomonas aeruginosa
WP_230697875.1#64|Pseudomonas kunmingensis
WP_053145658.1#65|Pseudomonas sp P97 38
WP_236378493.1#66|Pseudomonas marginalis
WP_218031589.1#67|Pseudomonas juntendi

Figure S5. Distant ShdA homologues can be encoded in DISARM-like operons. FlaGs output representing the genomic neighbourhood of distant ShdA homologues shows that these can be encoded within DISARM-like operons. Neighbouring genes are clustered together based on similarity and each cluster is numbered. Definition of neighbouring gene clusters is reported in Table S8
