# Supplementary material for: The novel anti-phage system Shield co-opts an RmuC domain to mediate phage defense across Pseudomonas species
Source: PLoS Genet. 2023 Jun 5;19(6):e1010784. doi: 10.1371/journal.pgen.1010784 (PMC10270631; doi:10.1371/journal.pgen.1010784)
Supplement: S6 Fig — Alignment of ShdA II homologues using MUSCLE. The alignment was coloured in Boxshade by percentage of identity. Sequence conservation between Shield- and DISARM-associated ShdA homologues is still present but is reduced to what observed in S2 Fig. (PDF) [file pgen.1010784.s018.pdf]

```
               ....:...10....:...20....:...30....:...40....:...50....:...60....:...70....:...80....:...90....:..100....:..110....:..120....:..130....:..140....:..150....:..160
WP_031691248.1 -----MEMAPVTLILIALVALVFLAVGWFIGGRMSTLKSARTQLEIQASHQSQLAQLNSDLALSRQSEAHIRGELEIAVQVRQQSVQQLDAVRAAANKLNAELSAAQATLQAELEKSQEKSRLLDEQAQQVIEHFNRQEALQQHVSALREQLGMSQEQ-A
WP_052156607.1 -----MEMAPVTLIFIALVALVFLAVGWFIGGRMTTLKSARTQLEIQASHQSQLAQLNSDLALSRQSEAHIRGELEMAVQARQQSVQQLDGVRAAANKLNAELSAAQATLQAELEKSQEKSRLLDEQAQQVIEHFNRQETLQQHVSALREQLGMSQEQ-A
WP_236693126.1 --MQASQWTTDFIVITAVLALGSALLGYFAAALRQGKQAGQLAAQLEQALQAQTSAIADQASLAEQLKSAQTSSNELQVAESGLHA-RLQSTIENMERLRAEQQESKDVSATLQLKLEDASRQHYETAKRLETAQAEGRALLEQVTDLRDRLAAAQTSMH
WP_241492828.1 --MQASQWTTDIIVITAVLAVVFALLGYFVAALRQGRQLSQLTAQLEQAHATQASAIAGQAALGEQLKAAEGRSHELQVAESKLNA-QLQSAFENVTRLNTEQQQSKDATATVQLKLEDANRQHHEAAKRLDTVQAEGRALQEQVTDLRDRLAAAQTSVL
WP_244651912.1 --MQASQWTTDIIVITAVLAVVFALLGYFVAALRQGRQLSQLTAQLEQAHATQASAIAGQAALGEQLKAAEGRSHELQVAESKLNA-QLQSAFENVTRLNTEQQQSKDATAAVQLKLEDANRQHHEAAKRLDTVQAEGRALQEQVTDLRDRLAAAQTSVL
WP_226489315.1 --MQSSQWTTDIVVITTVLAVASALLGYFAAALRQGRQLSQLTAQLEQSHTAQASISAEKAALTEQLKVADGRGHVLQVAESKLNA-QLQSAFENVTRLNTEQQHSRD----------------------------------------------------
WP_046266589.1 --MQSSQWTTDIVVITTVLAVASALLGYFAAALRQGRQLSQLTAQLEQSHTAQASISAEKAALTEQLKVADGRGHELQVAESKLNA-QLQSAFENVTRLNTEQQQSRD----------------------------------------------------
WP_209995266.1 --MQSSQWTTDIVVITTVLAVASALLGYFAAALRQGRQLSQLTAQLEQSHTAQASISAEKAALTEQLKVADGRGHELQVAESKLNA-QLQSAFENVTRLNTEQQQSRD----------------------------------------------------
WP_245097862.1 --MQASQWTTDIIVISTVLAVASALLGYFAAALRQGRQLSQLTAQLEQSHTAQASVIAEKTALTEQLKVAEGRGHELQVAESKLNA-QLQSAFENVTRLNAEQQQSRDVAAAVQLKLEDANRQHHEAAKRLDTAQAEGRAIHEQVTDLRDRLSAAQTSVL
WP_048352290.1 --MQASQWTTDIIVISTVLAVASALLGYFAAALRQGRQLSQLTAQLEQSHTAQASVIAEKAALTEQLKVAEGRGHELQVAESKLNA-QLQSAIENVTRLNTEQQQSRD----------------------------------------------------
WP_101157028.1 --MQASQWTTDIIVITTVLAVVSALLGYFAAALRQGRQLSQLTAQLEQSHTAQASVIAEKAALTEQLKVAEERGHGLQVAEGKLNA-QLQSALENVTRLNTEQQQSRD----------------------------------------------------
WP_150634623.1 --MLASQWMTEIIVISVVLAIASAVIGYFIAALRQGRQVSMLTEQLAQAQQGQASADAQQTVLQGQLKAAEEQCHNLQVEQGKLQT-QLHAAADTATRLSTDLQTSKD----------------------------------------------------
WP_122603527.1 --MLASQWTMEIIVISVVLAIASAVIGYFIAALRQGRQVSMLTAKLEQAQQGQASADAQQTALQGQLKAEEAQCHNLQVEQGKLQT-QLHAVADTATRLSADLLISKD----------------------------------------------------
WP_169991116.1 --MLASQWTMEIIVISVVLAIASAVIGYFIAALRQGRQVSTLTAQLEQAQQGQASADTQQTAQQGQLKAAEAQCHNLQVEQGRLQT-QLHAAADTATRLTADLITSKD----------------------------------------------------
WP_150743101.1 --MQASQLTTEIIVICVIAALASLVLGYVVAALRQGRQLSRLNSQLEQAQQAQVSAVAASTALSEQLNAAETRRQALQIEESALKA-KLQAAYENVTRLSQEQQAGKDAQVALQLKLDDASRQHHETAKRLETAQADGRALQEQVTGLRDRLNTAQTTII
WP_153428237.1 --MQASQLTTEIIVICVIAALASLVLGYVVAALRQGRQLSRLNSQLEQAQQAQVSAVAASTALSEQLNAAETRRQALQIEESALKA-KLQAAYENVTRLSQEQQAGKDAQVALQLKLDDASRQHHETAKRLETAQADGRALQEQVTGLRDRLNTAQTTII
WP_043216217.1 ----------------------------------------------------------------------------------------------------------------------------------------------------------------
WP_096219672.1 --MQASQWTPEIIVICAVLALAAAVIGFVLATLRQSRQINQLGAQLEQVAEAKTSTEAQSQILQSQLRESEGRSQALQVEEGKLKV-QLQASVETNARLMQELQDHKA----------------------------------------------------
WP_128723345.1 --MQASQWTPEIIVICAVLALAAAVIGFVLATLRQSRQINQLGAQLEQVAEAKTSTEAQSQILQSQLRESEGRSQALQVEEGKLKV-QLQASVETNARLMQELQDHKV----------------------------------------------------
WP_236225530.1 --MQASQWTPEIIVICAVLALAAAVIGFVLATLRQSRQINQLGAQLEQVAEAKTSTEAQSQILQSQLRESEGRSQALQVEEGKLKV-QLQASVETNARLMQELQDHKA----------------------------------------------------
WP_128733028.1 --MQASQWTPEIIVICAVLALAAAVIGFVLATLRQSRQINQLGAQLEQVAEAKTSTEAQSQILQSQLRESEGRSQALQVEEGKLKV-QLQASVETNARLMQELQDHKA----------------------------------------------------
WP_052155672.1 --MQASQWTPEIIVICAVLALAAAVIGFVLATLRQSRQINQLGAQLEQVAEAKTSTEAQSQILQSQLRESEGRSQALQVEEGKLKV-QLQASVETNARLMQELQDHKA----------------------------------------------------
WP_162953562.1 --MQASQWTPEIIVICAVLALAAAVIGFVLATLRQSRQINQLGAQLEQVAEAKTSTEAQSQILQSQLRESEGRSQALQVEEGKLKV-QLQASVETNARLMQELQDHKA----------------------------------------------------
WP_225656389.1 --MQASQWTTEIIVICGVLAIAAVVIGYVVAALRQGRQISQLSMQLEQAELARASTEAQQQSLQTQLKAAEARSHELQIEEGKLKA-QLHASADTAARLSTELHERKAAGSDLQVKLDEANRQHHEAAKRLEAVQAGARGLQEQVTELRGRLDTSTITNA
WP_236081858.1 --MQASQWTTEIIVICSVLAIATAVIGYVIAALRQGRQISQLSMQLEQAEQARASAETQQQSLQIQFKAAEACSHELQIEEGKLKA-QLQAAADTAARLSTELHERKAAGSDLQVKLEEANRQHHEAAKRLEAVQAGARGLQEQVTELRGRLDTSTTTNA
WP_089030887.1 ----------------------------------------------------------------------------------------------------------------------------------------------------------------
WP_003158586.1 ----------------------------------------------------------------------------------------------------------------------------------------------------------------
WP_016253785.1 --MQASQWTTEIIVICGVLAIAAAVIGYVVAALRQGRQISQLSMQLEQAELARASTEAQQQSLQIQLKAAEARSHELQIEEGKLKA-QLQASADTMARLSAELHERKAAGSDLQVKLEEANRQHHESARRLEAVQAGAQGLQEQVTELRGRLDTSTTTNA
WP_223716152.1 --MQASQWTTEIIVICGVLAIAAAVIGYVVAALRQGRQISQLSMQLEQAELARASTEAQQQSLQIQLKAAEARSHELQIEEGKLKA-QLQASADTMARLSAELHERKAAGSDLQVKLEEANRQHHESARRLEAVQAGAQGLQEQVTELRGRLDTSTTTNA
WP_241973209.1 --MQASQWTTEIIVICGVLAIAAAVIGYVVAALRQGRQISQLSMQLEQAELARASTEAQQQSLQIQLKAAEARSHELQIEEGKLKA-QLQASADTMARLSAELHERKAAGSDLQVKLEEANRQHHESARRLEVVQAGAQGLQEQVTELRGRLDTSTTTNA
WP_244245074.1 ----------------------------------------------------------------------------------------------------------------------------------------------------------------
WP_244260776.1 ----------------GVLAIAAAVIGYVVAALRQGRQISQLSMQLEQAELARASTEAQQQSLQIQLKAAEARSHELQIEEGKLKA-QLQASADTMARLSAELHERKAAGSDLQVKLEEANRQHHESARRLEAVQAGAQGLQEQVTELRGRLDTSTTTNA
WP_244251134.1 --MQASPWTTEIIVICGVLAIAAAVIGYVVAALRQGRQISQLSMQLEQAELARASTEAQQQSLQIQLKAAEARSHELQIEEGKLKA-QLQASADTMARLSAELHERKAAGSDLQVKLEEANRQHHESARRLEAVQAGAQGLQEQVTELRGRLDTSTTTNA
WP_121391426.1 ----------------------------------------------------------------------------------------------------------------------------------------------------------------
WP_225011004.1 --MQASQWTTEIIVICGVLAIAAAVIGYVVAALRQGRQIGQLSMQLEQAEQARASTETQQQSLQIQLKAVEARSHELQIEEGKLKA-QLQASADTAARLSTELYERKAAGSDLQVKLDEANRQHHESARRLEAVQAGARGLQEQVTELRGRLDTSTTTNA
WP_016851949.1 ----------------------------------------------------------------------------------------------------------------------------------------------------------------
WP_242554464.1 --MQASQWTTEIIVICGVLAIAAAVIGYVLAALRQGRQIGQLSMQLEQAEQARASTETQQQSLQIQLKAVEARSHELQIEEGKLKA-QLQASADTAARLSTELYERKAAGSDLQVKLDEANRQHHESARRLEAVQAGARGLQEQVTELRGRLDTSTTTNA
WP_023082747.1 --MQASQWTTEIIVICGVLAIAAAVIGYVLAALRQGRQIGQLSMQLEQAEQARASTETQQQSLQIQLKAVEARSHELQIEEGKLKA-QLQASADTAARLSTELYERKAAGSDLQVKLDEANRQHHESARRLEAVQAGARGLQEQVTELRGRLDTSTTTNA
WP_227788364.1 --MQASQWTTEIIVICGVLAIAAAVIGYVLAALRQGRQIGQLSMQLEQAEQARASTETQQQSLQIQLKAVEARSHELQIEEGKLKA-QLQASADTAARLSTELYERKAAGSDLQVKLDEANRQHHESARRLEAVQAGARGLQEQVTELRGRLDTSTTTNA
WP_236032886.1 --MQASQWTTEIIVICGGLAIAAAVISYVIAALRQGRQIGQLSMQLEQAEQAKASTETQQQSLQIQLKAVEARSHELQIEEGKLKA-QLQASADTAARFSTELHERKAAGSDLQVKLDEATRQHHEAARRLEAVQAGARGLQDQVTELRQRLDTSTTTNA
WP_244161003.1 --MQASQWTTEIIVICGVLAIAAAVIGYVIAALRQGRLLSQLSMQLEQAEQARASTETQQQSLQIQLKAAEARSHELQIEEGKLKA-QLQASADTAARFSTELHERKAVGSDLQVKLDEATRQHHEAAKRLEAVQVGARGLQDQVTELCQRLDTSTTTNA
WP_126622994.1 ----------------------------------------------------------------------------------------------------------------------------------------------------------------
WP_236079675.1 --MQASQWTTEIIVICGVLAIAAAVIGYVIAALRQGRQISQLSMQLEQAEQAKASTETQQQSLQIQLKAAEVRSHELQIEEGKLKA-QLQASADTAARFSTELHERKAAGSDLQVKLDEATRQHHEAAKRLEAVQAGARGLQDQMTELRQRLDTSTTTNA
WP_244539034.1 --MQASQWTTDIIVITAVLALAGVLLGYFAAALRQGRQLSLLAAQLEQAQQAQATAVAGSTVLSEQLKAAEARGHELQVAESKLKA-QLQAAFENVTRLNQEQQAGKDAQATLQLKLDDASRQHHEAAKRLETAQAEGRALQGQVSELRDRLTAEQTAVT
WP_070096661.1 ----------------------------------------------------------------------------------------------------------------------------------------------------------------
WP_230064411.1 --MQASQWTTDIIVITAVLALACALLGYFAAALRQGRQLSLLAAQLELAQQAQTTAVAGSAALSEQLKAAEARGHELQVAESKLKA-QLQAAFDNVTRLNQEQQEGKDAQAALQLKLDDSSRQYNEAAKRLETAQAEGRALQEQVSELRDRLTAAQTAIT
WP_058145757.1 MFGLGVSWL--VVGVCLVIVQGAGAALYWQVLKQREARA-------------------------------------------------------------------------------------------------------------------------
WP_025297754.1 MFGLGLSWL--VIGVCLLLVHGAGVALYWQILKQREARA-------------------------------------------------------------------------------------------------------------------------
WP_155664935.1 ----------------MLLVHGAGVALYWQILKQREARA-------------------------------------------------------------------------------------------------------------------------
WP_125834039.1 ----------------MLIVQGAGIALYWQVLKRREALA-------------------------------------------------------------------------------------------------------------------------
WP_106733167.1 ----------------MLLVQGAGIALYWQLLRRREAFA-------------------------------------------------------------------------------------------------------------------------
WP_049790913.1 ----------------MLLVQGAGIALYWQVLKRREAFA-------------------------------------------------------------------------------------------------------------------------
WP_237881090.1 -----MENLVILIVLPLVAFIVAFIVAWVIASWRAQRNNQQLQTDLDVTQADLRRVQAELSEHQATLGSLSGEKAALDIAYGRLET-ERDSGKARIERLEGDLVTQQAEIDRLRQSEQVARETSSKAQEAADKLQ-----------------DQLASNNT
WP_053145658.1 --MPASQWTPDIIAICVIAMIASATIAYLLAGLRQGRNVTELSARFKQSQQALEKVTSEQLETQAQLKGAELLNHQQQVAESRVQE-QLSSAKATADRLSAELIGRTA----------------------------------------------------
WP_236378493.1 --MPASQWTADFIAICVFAMIASATIAYLFAGLRQGRKVSELSARLEQSQQALEKVTSEQLEAHAQLKSAEMLNHQQQVTESRVQE-QLSSAKSTADRLSAELIGRTA----------------------------------------------------
WP_236080768.1 ----------------------------------------------------------------------------------------------------------------------------------------------------------------
WP_043087442.1 ----------------------------------------------------------------------------------------------------------------------------------------------------------------
WP_245655573.1 --MQASQWTTDIVVICAVLAVCCMLLGYLAAALRQGRQASQLAARLEQAEQARASAAAAQLDLGEQLKAAQARSQELLVGEGRLNE-QLQASTDNLARLVREQQDSKDAVALLQQKLEEGSRQHHEAVTRLETTQIEGQGLQRQVTELRERLGAEQTTVN
WP_199788277.1 --MQASQWTTDIIVITAVIALACALLGYCVTALRQGRQVSQLAAQLELAQQAQASAVAASAALSDQLKAADARGHELQVAESKLKA-QLQAAFDNVARLNQEQQESKDANARLQLKLEDAGRQHHEAAKRLETAQAEGRALQDQVTDLRDRLAVAQSGVT
WP_237262870.1 --MQASQWTTEIIVISAVLALASLVIGYFIATLRQGRQIGTLTAQLEQAQQAQCSAVAASAVLSEQLKAAEARSQELQVVESTLKA-QLQASFENVTRLNHELQESKDAKAAQNLKLEDANRQHHETAKRLETAQADSRSLQEQVTDLRDRLSTAQAAIT
WP_219807503.1 --MQASQWTTEIIVISAVLALASLVIGYFVATLRQGRQIGTLTAKLEQAQQGQTSAMAASTALSEQLKAAEARSQELQVAESTLKA-KLQAAFDNVTRLNHELQESKDAKAAQNLKLEDANRQHHETAKRLETAQADSRSLHEQVTDLRDRLSTAQAAIT
WP_221739997.1 --MQASQWTTEIIVISAVLALASLVIGYFIATLRQGRQIGTLTAQLEQAQQGQASAMAASTALSEQLKAAEARSQELQVAESTLKA-KLQAAFDNVTRLNHELQESKDAKAAQNLKLEDANRQHHETAKRLETAQADSRSLHEQVTDLRDRLSTAQAAIT
WP_230848231.1 --MQASQWTTEIIAISAVLALASLVIGYFIATLRQGRQIGTLTAQLEQAQQGQTSAMAASTALSEQLKAAEARSQELQVAESTLKA-KLQAAFDNVTRLNHELQESKDAKAAQNLKLEDANRQYHETAKRLETAQADSRSLHEQVTDLRDRLSTAQAAIT
WP_052154687.1 --MQASQWTTEIIVITAVLALTGVLLGYFAAALRQGRQLSLLAAQLEQAQQAQTTALAGSTALSDQLKAAEARGHELQVAESKLKA-QLQAAFDNVTRLNQEQQADKDAQAALQLKLDDASRQHHEAAKRLETAQAEGRALQEQVSELRDRLTAAQTAIT
WP_199865577.1 ----------------------------------------------------------------------------------------------------------------------------------------------------------------
WP_142990457.1 ----------------------------------------------------------------------------------------------------------------------------------------------------------------
WP_023095083.1 --MQASQWTTEIIVITAILALVCVLLGYFTAALRQGRQLSLLAAQLEQAQQAQATAVAGSAALSEQLKAAEARAHELQLAESTLKA-KLQAAFDNEARLNKEQQECKDSLAALQLKLDDASRQHHEVAKRLETAQAEGRALQGQVSELRDRLTAEQTAVT
WP_196655173.1 ----------------------------------------------------------------------------------------------------------------------------------------------------------------
WP_147318930.1 ----------------------------------------------------------------------------------------------------------------------------------------------------------------
WP_146537863.1 --MQASQWTTEIIVICVIAALASLVLGYVVAALRQGRQLSRLAVELEQAQQAQASAVAAGTALGEQLRAADARTQELQVAESALKA-KLQAALNNEARLSHEQQEAKD----------------------------------------------------
WP_209953570.1 --MQASQWTTEIIVICVIAALASLVLGYVVAALRQGRQLSRLAVELEQAQQAQASAVAAGTALSEQLRAADARTQELQVAESALKA-KLQAALNNEARLSHEQQEAKD----------------------------------------------------
WP_241490968.1 ----------------------------------------------------------------------------------------------------------------------------------------------------------------
WP_230697875.1 ----------------------------------------------------------------------------------------------------------------------------------------------------------------
WP_196173169.1 --MQASQWTTDIIVITVVLALACALLGHFAAALRQGRQLSQLAAELEQAQLAQTTAVAGSAALSEKLMAAEALVHELQVAESTLKT-KLQGAFDGVTRLTQEHQEAKD----------------------------------------------------
WP_218031589.1 ----------------------------------------------------------------------------------------------------------------------------------------------------------------
WP_193075126.1 ---MPLNEMRFLLPLLSLALLGGWLLTGWVLLRRE-----------------------------------------------------------------------------------------------------------------------------
WP_088177171.1 -----MSWG--MVSAALVLAAGLGLALFWQSMRRKEAHT-------------------------------------------------------------------------------------------------------------------------
WP_078452141.1 -----MSWG--MVAAALVLAAGLGLALFWQSMRRQEAHT-------------------------------------------------------------------------------------------------------------------------
WP_128708491.1 -----MSWG--MVAAALVLAAGLGLALFWQSMRRKEAHT-------------------------------------------------------------------------------------------------------------------------
WP_088136089.1 -----MSWG--MVAAALVLAAGLGLALFWQSMRRKEAHT-------------------------------------------------------------------------------------------------------------------------
WP_192329154.1 -----MSWG--VVGITLLVACVMALALFWQIMRRREAVA-------------------------------------------------------------------------------------------------------------------------
WP_187808583.1 -----MSWG--VAGIALLSVCVMALALFWQIMRRREAVA-------------------------------------------------------------------------------------------------------------------------
WP_110684028.1 -----MSWG--VAGIALLSVCVMALALFWQIMRRREAVA-------------------------------------------------------------------------------------------------------------------------
WP_051424555.1 ------------MGIALLAVCMTALAMFWQIMRRREAVS-------------------------------------------------------------------------------------------------------------------------

WP_061904584.1 -----MSWA--VMGVALLVVCVMALALFWQIMRRREVVG-------------------------------------------------------------------------------------------------------------------------
WP_143506126.1 -----MSWA--VMGIALLAVCMMALAMFWQIMRRREAVS-------------------------------------------------------------------------------------------------------------------------
WP_230697896.1 MNGIGMSWA--VVSIALLVVCVMALALFWQIMRRREALA-------------------------------------------------------------------------------------------------------------------------
WP_151134790.1 ----------------------------------------------------------------------------------------------------------------------------------------------------------------
WP_225024324.1 MSDIGMSWG--LLAVAMFLVIVLGVMVLWQGMRRKEVQD-------------------------------------------------------------------------------------------------------------------------
WP_073660362.1 --MIGLSWM--VISGSLLLVVLAGVAAYWQVLRRREAVA-------------------------------------------------------------------------------------------------------------------------
WP_244539033.1 MNMIGLSWM--VISGSLLLVVLAGVAAYWQVLRRREAVA-------------------------------------------------------------------------------------------------------------------------
WP_088294302.1 ----------MVISGSLLLVVLAGVAVYWQVLRRREAVT-------------------------------------------------------------------------------------------------------------------------
WP_237754537.1 MNMIALSWM--VISGSLLLVVLAGVAVYWQVLRRREAVT-------------------------------------------------------------------------------------------------------------------------
WP_179217122.1 ----------------------------------------------------------------------------------------------------------------------------------------------------------------
WP_088193918.1 --MIGLSWM--VISGSLLLVVLAGVAAYWQVLRRREAVA-------------------------------------------------------------------------------------------------------------------------
WP_088169980.1 --MIALSWM--VISGSLLLVVLAGVAVYWQVLRRREAVA-------------------------------------------------------------------------------------------------------------------------
WP_236081955.1 MNMIGLSWM--VISGSLLLVVLAGVAAYWQVLRRRKAVT-------------------------------------------------------------------------------------------------------------------------
WP_109933952.1 --MIGLSWM--VMSAGLLLVLLAGVAVYWQVLRRREAVT-------------------------------------------------------------------------------------------------------------------------
WP_070147860.1 MNMIGLSWM--VISGSLLLVVLAGVAVYWQVLRRREAVT-------------------------------------------------------------------------------------------------------------------------
WP_108116052.1 ----------MVISGSLLLVVLAGVAVYWQVLRRREAVT-------------------------------------------------------------------------------------------------------------------------
WP_079383059.1 MNMIGLSWM--AISGSLLLVVLAGVAVYWQVLRRREAVT-------------------------------------------------------------------------------------------------------------------------
WP_196476661.1 MNMIGLSWM--VISGSLLLVVLAGVAAYWQVLRRLEAVT-------------------------------------------------------------------------------------------------------------------------
WP_236079907.1 MNMIGLSWM--AISGSLLLVVLAGVAAYWQVLRRREAVT-------------------------------------------------------------------------------------------------------------------------
WP_073666500.1 --MIGLSWM--AISGSLLLVVLAGVAAYWQVLRRREAVT-------------------------------------------------------------------------------------------------------------------------
WP_125881257.1 --MIALSWM--AISGSLLLVVLAGVAAYWQVLRRREAVT-------------------------------------------------------------------------------------------------------------------------
WP_049264406.1 --MIGLSWI--VISSSLLLVVLAGVAVYWQVLRRREAIT-------------------------------------------------------------------------------------------------------------------------
WP_031633112.1 --MIGLSWI--VISSSLLLVVLAGVAVYWQVLRRREAIT-------------------------------------------------------------------------------------------------------------------------
WP_065426329.1 MNMIGLSWI--VISSSLLLVVLAGVAVYWQVLRRREAIT-------------------------------------------------------------------------------------------------------------------------
WP_238326948.1 MNMIGLSWI--VISSSLLLVVLAGVAVYWQVLRRREAIT-------------------------------------------------------------------------------------------------------------------------
WP_238954630.1 MNMIGLSWI--VISSSLLLVVLAGVAVYWQVLRRREAIT-------------------------------------------------------------------------------------------------------------------------
WP_023115263.1 MNMIGLSWI--VISSSLLLVVLAGVAVYWQVLRRREAIT-------------------------------------------------------------------------------------------------------------------------
WP_241510754.1 MNMIGLSWI--VISSSLLLVVLAGVAVYWQVLRRREAIT-------------------------------------------------------------------------------------------------------------------------
WP_124140907.1 ----------MVISGSLLLVVLAGVAVYWQVLRRREAVT-------------------------------------------------------------------------------------------------------------------------
WP_231740201.1 MNMIGLSWM--VISGSLLLVVLAGVAVYWQVLRRREAVT-------------------------------------------------------------------------------------------------------------------------
WP_237752443.1 ----------------------------------------------------------------------------------------------------------------------------------------------------------------
WP_233787826.1 ----------------------------------------------------------------------------------------------------------------------------------------------------------------
WP_240443035.1 MNMIALSWM--VISGSLLLVVLVGVAVYWQVLRRREAVA-------------------------------------------------------------------------------------------------------------------------
WP_071540622.1 MNMIGLSWM--AISGSLLLVVLAGVAVYWQVLRRREAVT-------------------------------------------------------------------------------------------------------------------------
WP_235581972.1 ----------------------------------------------------------------------------------------------------------------------------------------------------------------
WP_140786428.1 MNMIGLSWM--VISGSLLLIVLAVVAAYWQVLRRREAVA-------------------------------------------------------------------------------------------------------------------------
WP_228778169.1 MNMIGLSWI--VISSSLLLVVLAGVAVYWQVLRRREAIT-------------------------------------------------------------------------------------------------------------------------
WP_034017985.1 --MIGLSWI--VISSSLLLVVLAGVAVYWQVLRRREAIT-------------------------------------------------------------------------------------------------------------------------
WP_128704223.1 --MIGLSWI--VISSSLLLVVLAGVXVYWQVLRRREAIT-------------------------------------------------------------------------------------------------------------------------
WP_172792899.1 --MIGLSWM--AISGSLLLVVLAGVAAYWQVLRRREAVT-------------------------------------------------------------------------------------------------------------------------
WP_210982944.1 ----------------------------------------------------------------------------------------------------------------------------------------------------------------
WP_230964886.1 MNMIGLSWM--VISGSLLLVVLAGVAAYWQVLRRREAVA-------------------------------------------------------------------------------------------------------------------------
WP_088135368.1 --MIGLSWM--VISGSLLLVVLAGVAAYWQVLRRREAVA-------------------------------------------------------------------------------------------------------------------------
WP_078452129.1 MNMIGLSWM--VISGSLLLVVLAGVAVYWQVLRRREAIT-------------------------------------------------------------------------------------------------------------------------
WP_126571791.1 ----------MVISGSLLLVVLAGVAVYWQVLRRREAIT-------------------------------------------------------------------------------------------------------------------------
WP_043106458.1 --MIALSWM--VISGSLLLVVLAGVAVYWQVLRRREAVA-------------------------------------------------------------------------------------------------------------------------
WP_228761142.1 MNMIALSWM--VISGSLLLVVLAGVAVYWQVLRRREAVA-------------------------------------------------------------------------------------------------------------------------
WP_063837559.1 MNMIALSWM--VISGSLLLVVLAGVAVYWQVLRRREAVA-------------------------------------------------------------------------------------------------------------------------
WP_087786958.1 --MIALSWM--VISGSLLLVVLAGVAVYWQGLRRREAVA-------------------------------------------------------------------------------------------------------------------------
WP_025297452.1 --MIALSWM--VISGSLLLVVLAGVAVYWQVLRRREAVA-------------------------------------------------------------------------------------------------------------------------
WP_235584266.1 MNMIALSWM--VISGSLLLVVLAGVAVYWQVLRRREAVA-------------------------------------------------------------------------------------------------------------------------
WP_198421420.1 ----------MVISGSLLLVVLAGVAVYWQVLRRREAVT-------------------------------------------------------------------------------------------------------------------------
WP_232527384.1 MNMIALSWM--VISGSLLLVVLAGVAVYWQVLRRREAVT-------------------------------------------------------------------------------------------------------------------------
WP_234034845.1 ----------------------------------------------------------------------------------------------------------------------------------------------------------------
```

```
WP_052156607.1 NLSGELKASLAALQGRYEQAQAELQEHREQLGQSNEQAAQLVELKEQIRRFEQRHRETEDVVKSQREQLGQSSEQASQLLELKEKCRQFEQLLRDSE-----------EVLKGQREQLGQANE--------QRIQLGELRDKHIAREREISE--------
WP_236693126.1 SLNGERDALKAELANVDASAKVAATGEREARELLAELRQKLADQVQQYNELLGRYQPLSNQHAELNTSLQKREELVAELRDRLGQTQATNEQLHADR-----------DRLKDELAIEGKRAQSLETSERDVREQLSEVKLALTERAQANNA--------
WP_241492828.1 SLQSERGTLKDELANLDASAKVAATSEREAREQLAESRQKLASQVQGYNDLLERYQPLSNQHAELNTSLQKREEVVVELRDRLGQAQTTNEQLHADR-----------DRLKDELANEGKRAKSLETSERDVREQLGEAKQGLIERAQAINA--------
WP_244651912.1 SLQSERGTLKDELANLDASAKVAATSEREAREQLAESRQKLASQVQGYNDLLGRYQPLSNQHAELNTSLQKREEVVVELRDRLGQAQTTNEQLHADR-----------DRLKDELANEGKRAKSLETSERDVREQLGEAKHGLIERAQAINA--------
WP_226489315.1 ---------------------------------------AIAAVQVKLEEANQQHHEAAKRLDTAQAEGRALQERITELRDRLEKAQTSNEHLQADR-----------DRLKDELANEGKRAKSLETSERDVREQLGEAKQVLIERAQALNA--------
WP_046266589.1 ---------------------------------------AIAAVQVKLEEANRQHHEAAKRLDTAQAEGRALQERITELRDRLEKAQTSNEHLQADR-----------DRLKDELANEGKRAKSLETSDRDVREQLGEAKQVLIERAQALNA--------
WP_209995266.1 ---------------------------------------AIAAVQVKLEEANRQHHEAAKRLDTAQAEGRALQERITELRDRLEKAQTSTEHLQADR-----------DRLKDELANEGKRAKSLETSDRDVREQLGEAKQVLIERAQALNA--------
WP_245097862.1 SLQSERETIKDELANLDVSAKVAATSEREAREQLAELRQKLESQVQGYNELLGRYQPLSNQHAELNMSLQKREELVAELRDRLGKAQTSNDQLHADR-----------DRLKDELANEGKRAKSLETSERDVREQLSEAKQGLAERAHALNA--------
WP_048352290.1 ---------------------------------------VIAAVQLKLEDASRQHHEAAKRLDTVQAEGRAFQERVTDLRDRLEKAQTSNDQLQADR-----------DRLKDELANEGKRAKSLETSERDVREQLSEAKQALTERAQVLNA--------
WP_101157028.1 ---------------------------------------VIAAVHLKLEDANRQHHEAAKRLDTAQAEGRALQERVTDLRDRLEKAQTSNDQLQADR-----------DRLKDELANEGKRAKSLETSERDVREQLSEAKQALTERAQVLNA--------
WP_150634623.1 ---------------------------------------TGAALQLKLDEANQQHHETAKRLETAQAEGRSFQEQVTELRERLTAVETKNASMQTER-----------DGLKDELASEGKRAKALETSEREVREQLAEAKQTLAQQMESFNQ--------
WP_122603527.1 ---------------------------------------TGAALQLKLEEANQHHHETAKRLETVQAEVRGFQEQVAELRERLTAAETKNASMQTER-----------DGLKDELASEGKRAKALETSEREVREQLAEAKQTHAQQIESFNQ--------
WP_169991116.1 ---------------------------------------TSVALQLKLEEANQHHHETAKRLETAQAEVRGFQEQVAELRERLTAAETKNASMQTER-----------DCLKDELASEGKRAKALETSEREVREQLAETKQTLAQQMESFNQ--------
WP_150743101.1 SLQGERDGLKNELASLDASVQVAASEVREAREQLTETKQKLAEQTQSRAELQGRYEPLSNAHAELKTALEKREELVADLNDRLARTQLAGDQLNAER-----------DRLKDELASEGKRAKALETAEHEVREQLAETKQTLAKQVQSFSE--------
WP_153428237.1 SLQGERDGLKNELASLDASVQVAASEVREAREQLTETKQKLAEQTQSRAELQGRYEPLSNAHAELKTALEKREELVADLNDRLARTQLAGDQLNAER-----------DRLKDELASEGKRAKALETAEHEVREQLAETKQTLAKQVQSFSE--------
WP_043216217.1 -MQSERDGLKNELASLDASAQVAASEVREAREQLNDTKQKLAEQTQSRAELQGRYEPLSNAHAELKTALEKREELVADLNERLAKAQAAGLQLQAER-----------ESLKDELASEGKRAKALETAEREVREQLAETKQTLVKQVQSFSE--------
WP_096219672.1 ---------------------------------------VASDLQVKLDQANRQHHEAAKRLEAAQTSVQGQQEQMRELRERLDTTSTAKTALQEER-----------DRLKDALANEETRAKVAETSERDARAQLAETKQQLHEQVKAFGE--------
WP_128723345.1 ---------------------------------------VASDLQVKLDQANRQHHEAAKRLEAAQTSVQGQQEQMRELRERLDTTSTAKTALQEER-----------DRLKDALANEETRAKVAETSERDARAQLAETKQQLHEQVKAFGE--------
WP_236225530.1 ---------------------------------------VASDLQVKLDQANRQHHEAAKRLEAAQTSVQGQQEQMRELRERLDTTSTAKTALQEER-----------DRLKDALANEETRAKVAETSEHDARAQLAETKQQLHEQVKAFGE--------
WP_128733028.1 ---------------------------------------VASDLQVKLDQANRQHHEAAKRLEAAQTSVQGQQEQMRELRERLDTTSTAKTALQEER-----------DRLKDALANEETRAKVAETSEHDARAQLAETKQQLHEQVKAFGE--------
WP_052155672.1 ---------------------------------------VASDLQVKLDQANRQHHEAAKRLEAAQTSVQGQQEQMRELRERLDTTSTAKTALQEER-----------DRLKDALANEETRAKVAETSEHDARAQLAETKQQLHEQVKAFGE--------
WP_162953562.1 ---------------------------------------VASDLQVKLDQANRQHHEAAKRLEAAQTSVQGQQEQMRELRERLDTTSTAKTALQEER-----------DRLKDALANEETRAKVAETSEHDARAQLAETKQQLHEQVKAFGE--------
WP_225656389.1 ALQEERDRLKDALASEETRAKVAETAEREAREQLSETKHKLTEHVQALDTLQERYQSTSNEHAELKTSLDKSEKQVTELRERLAQAQATNEQLHTDR-----------DRLKDGLADEGKRAKALETAERDVRSQLLETKEALSQQVRSFNE--------
WP_236081858.1 ALQEERDRLKDALASEETRAKVAETAEREAREQLSETKYKLTEHVQALDALQKRYQSTSNEHAELKTSLDKSEKQVAELRERLAQAQTTNEQLHTDR-----------DRLKDGLADEGKRAKALETAERDVRSQLLETKEALSQQVRSFNE--------
WP_089030887.1 -MQEERDRLKDALASEETRAKVAETAEREAREQLREVKHKLTEHAQALDALQKRYQSTSNEHAELKTSLDKSEKQVTELRERLAQAQATNEQLHTDR-----------DRLKDGLADEGKRAKALETAERDVRSQLLETKEALSQQVRSFNE--------
WP_003158586.1 -MQEERDRLKDALASEETRAKVAETAEREAREQLNEIKQKLVEHAQAFDALQARYQSTSNEHAELGTALDKSEKQVAELRERLAQAQTTNEQLHTDR-----------DRLKDGLADEGKRAKALETAERDVRSQLLETKEALSQQVRSFNE--------
WP_016253785.1 ALQEERDRLKDALASEETRAKVAETAEREAREQLSETKHKLTEHVQALDTLQKRYQSTSNEHAELKTSLDKSEKQVAELRERLAQAQATNEQLHTDR-----------DRLKDGLADEGKRAKALETAERDVRSQLLETKEALSQQVRSFNE--------
WP_223716152.1 ALQEERDRLKDALASEETRAKVAETAEREAREQLNEIKQKLVEHAQAFDALQARYQSTSNEHAELGTALDKSEKQVAELRERLAQAQTTNEQLHTDR-----------DRLKDGLADEGKRAKALETAERDVRSQLLETKEALSQQVRSFNE--------
WP_241973209.1 ALQEERDRLKDALASEETRAKVAETAEREAREQLSETKHKLTEHVQALDTLQKRYQSTSNEHAELKTSLDKSEKQVAELRERLAQAQATNEQLHTDR-----------DRLKDGLADEGKRAKALETAERDVRSQLLETKEALSQQVRSFNE--------
WP_244245074.1 ------------MASEETRAKVAETAEREAREQLNEIKQKLVEHAQAFDALQARYQSTSNEHAELGTALDKSEKQVAELRERLAQAQTTNEQLHTDR-----------DRLKDGLADEGKRAKALETAERDVRSQLLETKEALSQQVRSFNE--------
WP_244260776.1 ALQEERDRLKDALASEETRAKVAETAEREAREQLNEIKQKLVEHAQAFDALQARYQSTSNEHAELGTALDKSEKQVAELRERLAQAQTTNEQLHTDR-----------DRLKDGLADEGKRAKALETAERDVRSQLLETKEALSQQVRSFNE--------
WP_244251134.1 ALQEERDRLKDALASEETRAKVAETAEREAREQLNEIKQKLVEHAQAFDALQARYQSTSNEHAELGTALDKSEKQVAELRERLAQAQTTNEQLHTDR-----------DRLKDGLADEGKRAKALETAERDVRSQLLETKEALSQQVRSFNE--------
WP_121391426.1 ----------------------------------------------------------------------------------------------------------------------------------------------------------------
WP_225011004.1 ALQEERDRLKDALASEETRAKVAETAEREAREQLNEIKQKLVEHAQAFDALQARYQSTSNEHAELGTALDKSEKQVAELRERLVQAQATNEQLHTDR-----------DRLKDGLADEGKRAKALETAERDVRNQLLETKEALSQQVRSFNE--------
WP_016851949.1 -MQEERDRLKDALASEETRAKVAETAEREAREQLNEIKQKLVEHAQAFDALQARYQSTSNEHAELGTALDKSEKQVAELRERLAQAQATNEQLHTDR-----------DRLKDGLADEGKRAKALETAERDVRNQLLETKEALSQQVRSFNE--------
WP_242554464.1 ALQEERDRLKDALASEETRAKVAETAEREAREQLNEIKQKLVEHAQAFDALQARYQSTSNEHAELGTALDKSEKQVAELRERLAQAQATNEQLHTDR-----------DRLKDGLADEGKRAKALETAERDVRNQLLETKEALSQQVRSFNE--------
WP_023082747.1 ALQEERDRLKDALASEETRAKVAETAEREAREQLNEIKQKLVEHAQAFDALQARYQSTSNEHAELGTALDKSEKQVAELRERLAQAQATNEQLHTDR-----------DRLKDGLADEGKRAKALETAERDVRNQLLETKEALSQQVRSFNE--------
WP_227788364.1 ALQEERDRLKDALASEETRAKVAETAEREAREQLNEIKQKLVEHAQAFDALQARYQSTSNEHAELGTALDKSEKQVAELRERLAQAQATNEQLHTDR-----------DRLKDGLADEGKRAKALETAERDVRNQLLETKEALSQQVRSFNE--------
WP_236032886.1 ALQEERDSLKDALATEETRAKVAETAEREAREQLNEIKQKLVEHAQAFDALQARYQSTSNEHAELGTALDKSEKQVAELRERLAQAQATNEQLHTDR-----------DRLKDGLADEGKRAKALETAERDVRNQLLETKEALSQQVRSFNE--------
WP_244161003.1 ALQEERDSLKDALATEETRAKVAETAEREAREQLNEIKQKLVEHAQAFDALQARYQSTSNEHAELGTALDKSEKQVAELRERLAQAQATNEQLHTDR-----------DRLKDGLADEGKRAKALETAERDVRNQLLETKEALSQQVRSFNE--------
WP_126622994.1 -MQEERDSLKDALATEETRAKVAETAEREAREQLNEIKQKLVEHAQAFDALQARYQSTSNEHAELGTALDKSEKQVAELRERLAQAQATNEQLHTDR-----------DRLKDGLADEGKRAKALETAERDVRNQLLETKEALSQQVRSFNE--------
WP_236079675.1 ALQEERDSLKDALATEETRAKVAETAEREAREQLNEIKQKLVEHAQAFDALQARYQSTSNEHAELGTALDKSEKQVAELRERLAQAQATNEQLHTDR-----------DRLKDGLADEGKRAKALETAERDVRNQLLETKEALSQQVRSFNE--------
WP_244539034.1 SLQGERDTLKDELADLDASAKVAATGEREAREQLADIRQKMAAQAQEYNELLGRYQPLSNQHAELKTSLQKREELVAELRDRLAQAQTTNEQLHADR-----------DRLKDELAAEGKRAKSLETSEHDARAQLAETKQQLHEQVKTFGE--------
WP_070096661.1 -MQGERDTLKDELANLDASAKVAATGEREAREQLAEIRQKMAAQALEYNELLGRYQPLSNQHAELKTALDNSGKQVTELRDRLAQAQTTNEQLHADR-----------DRLKDELAAEGKRAKSLETSERDAREQLAETKQHLNEQVKAFGE--------
WP_230064411.1 SLQGERDTLKDELANLDASAKVAATGEREAREQLAEIRQKMAAQALEYNELLGRYQPLSNQHAELKTALDNSGKQVTELRDRLAQAQTTNEQLHADR-----------DRLKDELAAEGKRAKSLETSERDAREQLAETKQHLNEQVKAFGE--------
WP_058145757.1 ---------------------------------------NLQASQTKLEYLQMRVHQQEDEIRELTAGLRAERAHVEQLKRQLEFAQGQAMQMRHDS---------------------------------------------------------------
WP_025297754.1 ---------------------------------------NLHASQTKLEYLQMRVHQQEDEIRELTAGLRAERAHVEQLKRQLEFAQGQAMQMRHDS---------------------------------------------------------------
WP_155664935.1 ---------------------------------------NLHASQTKLEYLQMRVHQQEDEIRELTAGLRAERAHVEQLKRQLEFAQGQAMQMRHDS---------------------------------------------------------------
WP_125834039.1 ---------------------------------------SLQSLQAQLKQSLMRVYQQEDEIRELTAGLRAEKAHVEQLQRQLEFAQEQARQIHHNG---------------------------------------------------------------
WP_106733167.1 ---------------------------------------SLQNVQAQLKQSLMRVYQQEDEIRELTAGLRAEKAHVEQLQRQLEFTQEQARQRHHDC---------------------------------------------------------------
WP_049790913.1 ---------------------------------------SLQNVQAQLKQSLMRVYQQEDEIRELTAGLRAEKAHVEQLQRKLEFAQEQARQRHHDC---------------------------------------------------------------
WP_237881090.1 AFKERLEEKDQRILDLDRKQVAAELQQTNTAKLLSESREALKQAQTQLEEKQVVQERFERDWGTQKAELETLRKTEKQAREVGATAQESANKLKEQLVINGTTSKERIEEKDQRIVQLDEKLATADVAQKHTAAQLAEIRETLKKAQTQLEEKQEALTNY
WP_053145658.1 ---------------------------------------DLDALAQKLEQAIQQRHMAEKLNENAQVDIRALKEQVSELRDRLGQAESAVQEERKAV-----------SDLKEELAQEGKKAKTFEAGEKEAREQLQERKSALGAAAEKHDA--------
WP_236378493.1 ---------------------------------------DLDALAQKLEQAIQQGHSVEKQNENAQVDIRALKAQTSELRDRLGQAESAVQEERKVV-----------SDLKEELAQEGKKAKTFEAGEKEAREQLQERKLALGAAAEKHDA--------
WP_236080768.1 ----------------------------------------------------------------------------------------------------------------------------------------------------------------
WP_043087442.1 ----------------------------------------------------------------------------------------------------------------------------------------------------------------
WP_245655573.1 TLQGERNTLKDELSAMDASAKVATTGEREAREQLAELRQKLAEQVQVYKELMGRYQPLSNQHAELRTALGKGEEVVTDLRDRLNAAQLASAQLQTGW-----------NQCKDVLASEGQRSKALETSEQELRQQLQDIKAQQISAAERYED--------
WP_199788277.1 SLQGERDTLKDELANLDASAKVAATGEREAREQLAEIRQKMVIQAQDYNELLGRYQPLSNQHAELKTSLHKREELVAELRDRLNTAQAASAQLQVDR-----------DQLKDELANEGKRSRALETSEREVREQLLDIKAQQVAAAERYEA--------
WP_237262870.1 SLQGERDNLKNELASLDASAKVAATVEREAREQLAEIRQQLASRTQQYNELLGRYQPLSNQHAELNTSLQKREELVAELRDRLNAAETVNAQLQTDR-----------DQLKDSLANEGKRSQSLATSEREVREQLQDIKAQQVAAAERYEA--------
WP_219807503.1 SLQGERDNLKNELGCLDASAKVAATGEREAREQLAEIRQQLASRTQQYNELLGRYQPLSNQHAELNTSLQKREELVAELRDRLSAAETVNAQLQTGR-----------DQLKDSLANEGKRSQSLATSEREVREQLQDIKAQQVAATERYEA--------
WP_221739997.1 SLQGERDNLKNELASLDASAKVAATGEREAREQLAEIRQQLASRTQQYNELLGRYQPLSNQHAELNTSLQKREELVAELRDRLNAAEAVNAQLQADR-----------DQLKDSLANEGKRSQSLATSEREVREQLQDIKAQQVAAAERYEA--------
WP_230848231.1 SLQGERDKLKNELASLDASAKVAATGEREAREQLAEIRQQLASRTQQYNELLGRYQPLSNQHAELNTSLQKREELVAELRDRLNAAETVNAQLQTDR-----------DQLKDSLANEGKRSQSLATSEREVREQLQDIKAQQVAAAERYEA--------
WP_052154687.1 SLQGERDTLKDELANLDASAKVAATGEREAREQLAEIRQKMAAQAQDYNELLGRYQPLSNQHAELKTSLHKREELVAELRDRLQAAEAVSAQLQTDR-----------DQLKDALASEGQRSKALETSEREVREQLQDIKAQQVAAAERYDA--------
WP_199865577.1 ----------------------------------------MAAQAQDYNELLGRYQPLSNQHAELKTSLHKREELVAELRDRLQAAEAVSAQLQTDR-----------DQLKDALASEGQRSKALETSEREVREQLQDIKAQQVAAAERYDA--------
WP_142990457.1 ---------------------------------------KMAAQAQDYNELLGRYQPLSNQHAELKTSLHKREELVAELRDRLQAAEAVSAQLQTDR-----------DQLKDALASEGQRSKALETSEREVREQLQDIKAQQVAAAERYDA--------
WP_023095083.1 SLQGERDNLKDELADLDASAKVAATGEREAREQLAEIRQKMAAQAQDYNELLGRYQPLSNQHAELKTSLHKREELVAELRDRLQAAEAVSAQLQTDR-----------DQLKDALASEGQRSKALETSEREVREQLQDIKAQQVAAAERYDA--------
WP_196655173.1 ----------------------------------------MAAQAQDYNELLGRYQPLSNQHAELKTSLHKREELVAELRDRLQAAEAVSAQLQTDR-----------DQLKDALASEGQRSKALETSEREVREQLQDIKAQQVAAAERYDA--------
WP_147318930.1 ---------------------------------------------------------------------------------SRRGFSPKSVSGISRPL--------------------------------------------------------------
WP_146537863.1 ---------------------------------------RYATLEIKLEQSTKQHHQAVTQHETAQTEVRSLKANVEDYQKRLELAQADLQKEREAV-----------SQLKSELAQEGKNAKGFEEANNEARAQLADLKGQLKDADQKLET--------
WP_209953570.1 ---------------------------------------RYATLEIKLEQSTKQHHLAVTQHETAQTEVRSLKANVEDYQKRLELAQADLQKEREAV-----------SQLKSELAQEGKNAKGFEEANNEARAQLADLKGQLKGADDKLAA--------
WP_241490968.1 -------------------------------------------------------------------------------------MKVADEKLET-----------------------------------------------------------------
WP_230697875.1 -------------------------------------------------------------------------------------MKVADEKLET-----------------------------------------------------------------
WP_196173169.1 ---------------------------------------RHATLEAKLEQSVKQHHEAVIQHETAVTEIRSLNAKVEDYQSRLEQANTLLQQERESV-----------SGLKSALAEEGKKAKGFEAANTDARTQLAELKEQMKTAGEKIET--------
WP_218031589.1 -------------------------------------------------------------------------------------MKTAGDKVET-----------------------------------------------------------------
WP_193075126.1 -----------------------------------------RLLQQRVDELQEESYRQQLELHQQLGSQLSLETRLEHLQESVARLRTERDRLATRL------------TEAEGQGNEWQRSAERFQTQVQEQQRQREQLHGERQTLEQRLQQ--------
WP_088177171.1 ---------------------------------------HLQACKDQLEQLQALGQSRELELRELTAAVRAEKAHVEQLQRQLSFVHEESAGLRDEQ-----------KQLLTQLAEAQSQASAARAQHGQLLQQHAELKQAHERLQNSHEV--------
WP_078452141.1 ---------------------------------------SLQACKDQLEQLQALSQSRELELRELTAAVRAEKAHVEQLQRQLSFAHEESAGLRDEQ-----------KQLLTQLADAQSQASAARAQHGQLLQQHAELKQAHERLQNSHEV--------
WP_128708491.1 ---------------------------------------SLQACKDQLEQLQALSESRELELRELTAAVRAEKAHVEQLQRQLSFAHEESAGLRDEQ-----------KQLLTQLADAQSQASAARAQYGQLLQQXAELKQAHECLQNSHEV--------
WP_088136089.1 ---------------------------------------SLQACKDQLEQLQALSESRELELRELTAAVRAEKAHVEQLQRQLSFAHEESAGLRDEQ-----------KQLLTQLADAQSQASAARAQYGQLLQQHAELKQAHECLQNSHEV--------
WP_192329154.1 ---------------------------------------GLQHYLLQLGQMQQQSQQQEAELREQTAAARAEKAHAEQLQRRMEFAQEELATLRDEQ-----------KQQLARLVEAQSQASAASAQHTQLAQQHSELKQTHERLQSNHEV--------
WP_187808583.1 ---------------------------------------TLQHYQLQLGQLQLQNQQQEAELREQTAAARAEKAHAEQLQRQLDFAQEELAGLRDEQ-----------KQLLTQLADAQSQASAARAQHSQLLQQHAELKQSHERLQNSHEV--------
WP_110684028.1 ---------------------------------------TLQHYQLQLGQLQQQNQQQETDLREQTAAARAEKAHAEQLQRQLDFAQEELAGLRDEQ-----------KQLLAQLADAQSQASAARAQHGQLLQQHAELKQSHERLQSSHEV--------
WP_051424555.1 ---------------------------------------SLQHYQLQLEQLQQQSQQQEADLREQTAAARAEKAHAEQLQRQLDFAQEELAALRDEQ-----------RQQLARFAESQSQASAARAQHGQLVQQHAELKQAHERLQNSHEV--------

WP_061904584.1 ---------------------------------------TLQHYQQQLGQLQQQNQQQEADLREQTAAARAEKAHAEQLQRQLDFAQEELTALRDEQ-----------KLQLSRFAEVQSQASAARAQHGQLLQQHAELKQSHERLQNSHEV--------
WP_143506126.1 ---------------------------------------SLQHYQLQLEQLQQQSQQQEADLREQTAAARAEKAHAEQLQRQLDFAQEELAALRDEQ-----------RQQLARFAEAQSQASASRAQHGQLLQQHAELKQSHERLQNSHEV--------
WP_230697896.1 ---------------------------------------ALQHYQLQLEQLQQQSQQQEADLREQTAAARAEMAHAEQLQRQLDFAQEELTALRDEQ-----------RQQLARFAEAQSQASAARAQHGQLVQQHSELKQAHERLQNSHEV--------
WP_151134790.1 ----------------------------------------------------------------------------------------------------------------------------------------------------------------
WP_225024324.1 ---------------------------------------NLQECKDQLEQAQGISQSRELELRELSAAFRAEKAHVEQLQRQVSYAQEEILGLREEQ-----------KQLLEKLAEAQSQTSAARAQHGQLLQQHGELKHTHERLQSSHEA--------
WP_073660362.1 ---------------------------------------ALQVCQSQLEQHMAASHQKDDELRELSAGLRAEKAHVEQLQRQVEFAQQEASRLRTEY-----------RDQSAQLVEVQSAASAARAQYGQLLQQHDELKQASARLQQVHDV--------
WP_244539033.1 ---------------------------------------ALQVCQSQLEQHMAASHQKDDELRELSAGLRAEKAHVEQLQRQVEFAQQEASRLRTEY-----------RDQSAQLVEVQSAASAARAQYGQLLQQHDELKQASARLQQVHDV--------
WP_088294302.1 ---------------------------------------ALQICQSQLEQHMAASHQKDDELRELSAGLRAEKAHVEQLQRQVEFAQQEAGRLRTEY-----------RDQFAQLAEVQSAASAARAQYGQLLQQHDELKQASARLQQVHDV--------
WP_237754537.1 ---------------------------------------ALQICQSQLEQHMAASHQKDDELRELSAGLRAEKAHVEQLQRQVEFAQQEAGRLRTEY-----------RDQFAQLAEVQSAASAARAQYGQLLQQHDELKQASARLQQVHDV--------
WP_179217122.1 ----------------------------------------------------------------------------------------------------------------------------------------------------------------
WP_088193918.1 ---------------------------------------ALQVCQSQLEQHMAASHQKDDELRELSAGLRAEKAHVEQLQRQVEFAQQEASRLRTEY-----------RDQSAQLVEVQSAASAARAQYGQLLQQHDELKQASVRLQQVHDV--------
WP_088169980.1 ---------------------------------------ALQICQSQLEQHMAASHQKDDELRELSAGLRAEKAHVEQLQRQVEFAQQEASRLRTEY-----------HDQFAQLVEVQSAASAARAQYGQLLQQHDELKQASIRLQQVHDV--------
WP_236081955.1 ---------------------------------------ALQICQSQLEQHMVASHQKDDELRELSAGLRAEKAHVEQLQRQVEFAQQEAGRLRTEY-----------RDQSAQLVEVQSAASAARAQYGQLLQQHDELKQASTRLQQVHDV--------
WP_109933952.1 ---------------------------------------ALQICQSQLEQRMAASQQKDDELRELSAGLRAEKAHVEQLHRQVEFAQQEAGRLRAEY-----------RDQSSQLAEVQSAASAARAQYGQLLQQHDELKQASTRLQQVHDV--------
WP_070147860.1 ---------------------------------------ALQICQSQLEQHMAASHQKDDELRELSAGLRAEKAHVEQLQRQVEFAQQEAGRLRTEY-----------RDQSAQLAEVQSAASAARAQYGQLLQQHDELKQASVRLQQVHDV--------
WP_108116052.1 ---------------------------------------ALQICQSQLEQHMAASHQKDDELRELSAGLRAEKAHVEQLQRQVEFAQQEAGRLRTEY-----------RDQSAQLAEVQSAASAARAQYGQLLQQHDELKQASVRLQQVHDV--------
WP_079383059.1 ---------------------------------------ALQICQSQLEQHMAASHQKDDELRELSAGLRAEKAHVEQLQRQVEFAQQEAGRLRTEY-----------RDQSAQLAEVQSAASAARAQYGQLLQQHDELKQASARLQQVHDV--------
WP_196476661.1 ---------------------------------------ALQICQSQLEQHMVASHQKDDELRELSAGLRAEKAHVEQLQRQVEFAQQEAGRLRTEY-----------RDQSAQLVEVQSAASAARAQYGQLLQQHDELKQASTRLQQVHDV--------
WP_236079907.1 ---------------------------------------ALQICQSQLEQHMVASHQKDDELRELSAGLRAEKAHVEQLQRQVEFAQQEAGRLRTEY-----------RDQSAQLVEVQSAASAARAQYGQLLQQHDELKQASTRLQQVHDV--------
WP_073666500.1 ---------------------------------------ALQICQSQLEQHMVASHQKDDELRELSAGLRAEKAHVEQLQRQVEFAQQEAGRLRTEY-----------RDQSAQLVEVQSAASAARAQYGQLLQQHDELKQASTRLQQVHDV--------
WP_125881257.1 ---------------------------------------ALQICQSQLEQHMVASHQKDDELRELSAGLRAEKAHVEQLQRQVEFAQQEAGRLRTEY-----------RDQSAQLVEVQSAASAARAQYGQLLQQHDELKQASTRLQQVHDV--------
WP_049264406.1 ---------------------------------------ALQICQSQLEQHMAASHQKDDELRELSAGLRAEKAHVEQLHRQVEFAQQEAGRLRTEY-----------RDQSAQLAEVQSAASAARAQYGQLLQQHDELKQASVRLQQVHDV--------
WP_031633112.1 ---------------------------------------ALQICQSQLEQHMAASHQKDDELRELSAGLRAEKAHVEQLHRQVEFAQQEAGRLRTEY-----------RDQSAQLAEVQSAASAARAQYGQLLQQHDELKQASVRLQQVHDV--------
WP_065426329.1 ---------------------------------------ALQICQSQLEQHMAASHQKDDELRELSAGLRAEKAHVEQLHRQVEFAQQEAGRLRTEY-----------RDQSAQLAEVQSAASAARAQYGQLLQQHDELKQASVRLQQVHDV--------
WP_238326948.1 ---------------------------------------ALQICQSQLEQHMAASHQKDDELRELSAGLRAEKAHVEQLHRQVEFAQQEAGRLRTEY-----------RDQSAQLAEVQSAASAARAQYGQLLQQHDELKQASVRLQQVHDV--------
WP_238954630.1 ---------------------------------------ALQICQSQLEQHMAASHQKDDELRELSAGLRAEKAHVEQLHRQVEFAQQEAGRLRTEY-----------RDQSAQLAEVQSAASAARAQYGQLLQQHDELKQASVRLQQVHDV--------
WP_023115263.1 ---------------------------------------ALQICQSQLEQHMAASHQKDDELRELSAGLRAEKAHVEQLHRQVEFAQQEAGRLRTEY-----------RDQSAQLAEVQSAASAARAQYGQLLQQHDELKQASVRLQQVHDV--------
WP_241510754.1 ---------------------------------------ALQICQSQLEQHMAASHQKDDELRELSAGLRAEKAHVEQLHRQVEFAQQEAGRLRTEY-----------RDQSAQLAEVQSAASAARAQYGQLLQQHDELKQASVRLQQVHDV--------
WP_124140907.1 ---------------------------------------ALQVCQSQLEQHMAASHLKDDELRELSAGLRAEKAHVEQLQRQVEFAQQEASRLRTEY-----------RDQSAQLAEVQSAASAARAQYGQLLQQHDELKQASVRLQQVHDV--------
WP_231740201.1 ---------------------------------------ALQVCQSQLEQHMAASHLKDDELRELSAGLRAEKAHVEQLQRQVEFAQQEASRLRTEY-----------RDQSAQLAEVQSAASAARAQYGQLLQQHDELKQASVRLQQVHDV--------
WP_237752443.1 ----------------------------------------------------------------------------------------------------------------------------------------------------------------
WP_233787826.1 ---------------------------------------------------------------------------------------------MRTEY-----------RDQSAQLAEVQSAASAARAQYGQLLQQHDELKQASIRLQQVHDV--------
WP_240443035.1 ---------------------------------------ALQICQSQLEQHMAESHQKDDELRELSAGLRAEKAHVEQLQRQVEFAQQEAGRLRTEY-----------RDQSAQLAEVQSAASAARAQYGQLLQQHDELKQASARLQQVHDV--------
WP_071540622.1 ---------------------------------------ALQICQSQLEQHMAASHQKDDELRELSAGLRAEKAHVEQLQRQVELAQQEAGRLRTEY-----------RDQSAQLAEVQSAASAARAQYGQLLQQHDELKQASARLQQVHDV--------
WP_235581972.1 ----------------------------------------------------------------------------------------------------------------------------------------------------------------
WP_140786428.1 ---------------------------------------ALQVCQSQLEQHMAASHQKDDELRELSAGLRAEKAHVEQLQRQVEFAQQEAGRLRTEY-----------RDQSAQLVEVQSAASAARAQYGQLLQQHDELKQASIRLQQVHNV--------
WP_228778169.1 ---------------------------------------ALQICQSQLEQHMAASHQKDDALRELSAGLRAEKAHVEQLQRQVEFAQQEAGRLRTEY-----------RDQSAQLVEVQSAASAARAQYGQLLQQHDELKQASIRLQQVHDV--------
WP_034017985.1 ---------------------------------------ALQICQSQLEQHMAASHQKDDALRELSAGLRAEKAHVEQLQRQVEFAQQEAGRLRTEY-----------RDQSAQLVEVQSAASAARAQYGQLLQQHDELKQASIRLQQVHDV--------
WP_128704223.1 ---------------------------------------ALQICQSQLEQHMAASHQKDDALRELSAGLRAEKAHVEQLQRQVEFAQQEAGRLRTEY-----------RDQSAQLVEVQSAASAARAQYGQLLQQHDELKQASIRLQQVHDV--------
WP_172792899.1 ---------------------------------------ALQICQSQLEQHMVASHQKDDELRELSAGLRAEKAHVEQLQRQVEFAQQEAGRLRTEY-----------RDQSAQLVEVQSTASAARAQYSQLLQQHDELKQASTRLQQVHDV--------
WP_210982944.1 ----------------------------------------------------------------------------------------------------------------------------------------------------------------
WP_230964886.1 ---------------------------------------ALQVCQSQLEQHMAASHQKDDELRELSAGLRAEKAHVEQLQRQVEFAQQEASRLRTEY-----------RDQSAQLIEVQSAASAARAQYGQLLQQHDELKQASIRLQQVHDV--------
WP_088135368.1 ---------------------------------------ALQVCQSQLEQHMAASHQKDDELRELSAGLRAEKAHVEQLQRQVEFAQQEASRLRTEY-----------RDQSAQLIEVQSAASAARAQYGQLLQQHDELKQASIRLQQVHDV--------
WP_078452129.1 ---------------------------------------ALQICQSQLEQHMAASHQKDDELRELSAGLRAEKAHVEQLQRQVEFAQQEASRLRTEY-----------RDQSAQLAEVQSAASAARAQYGQLLQQHDELKQASVRLQQVHDV--------
WP_126571791.1 ---------------------------------------ALQICQSQLEQHMAASHQKDDELRELSAGLRAEKAHVEQLQRQVEFAQQEASRLRTEY-----------RDQSAQLAEVQSAASAARAQYGQLLQQHDELKQASVRLQQVHDV--------
WP_043106458.1 ---------------------------------------ALQICQSQLEQHMAASHQKDDELRELSAGLRAEKAHVEQLQRQVEFAQQEASRLRTEY-----------HDQFAQLVEVQSAASAARAQYGQLLQQHDELKQASIRLQQVHDV--------
WP_228761142.1 ---------------------------------------ALQICQSQLEQHMAASHQKDDELRELSAGLRAEKAHVEQLQRQVEFAQQEASRLRTEY-----------HDQFAQLVEVQSAASAARAQYGQLLQQHDELKQASIRLQQVHDV--------
WP_063837559.1 ---------------------------------------ALQICQSQLEQHMAASHQKDDELRELSAGLRAEKAHVEQLQRQVEFAQQEASRLRTEY-----------HDQFAQLVEVQSAASAARAQYGQLLQQHDELKQASIRLQQVHDV--------
WP_087786958.1 ---------------------------------------ALQICQSQLEQHMAASHQKDDELRELSAGLRAEKAHVEQLQRQVEFAQQEASRLRTEY-----------HDQFAQLVEVQSAASAARAQYGQLLQQHDELKQASIRLQQVHDV--------
WP_025297452.1 ---------------------------------------ALQICQSQLEQHMAASHQKDDELRELSAGLRAEKAHVEQLQRQVEFAQQEASRLRTEY-----------HDQFAQLVEVQSAASAARAQYGQLLQQHDELKQASIRLQQVHDV--------
WP_235584266.1 ---------------------------------------ALQICQSQLEQHMAASHQKDDELRELSAGLRAEKAHVEQLQRQVEFAQQEASRLRTEY-----------HDQFAQLVEVQSAASAARAQYGQLLQQHDELKQASIRLQQVHDV--------
WP_198421420.1 ---------------------------------------ALQICQSQLEQHMAASHQKDDELRELSAGLRAEKAHVEQLQRQVEFAQQEASRLRTEY-----------HDQFAQLVEVQSAASAARAQYGQLLQQHDELKQASIRLQQVHDV--------
WP_232527384.1 ---------------------------------------ALQICQSQLEQHMAASHQKDDELRELSAGLRAEKAHVEQLQRQVEFAQQEASRLRTEY-----------HDQFAQLVEVQSAASAARAQYGQLLQQHDELKQASIRLQQVHDV--------
WP_234034845.1 ----------------------------------------------------------------------------------------------------------------------------------------------------------------
```

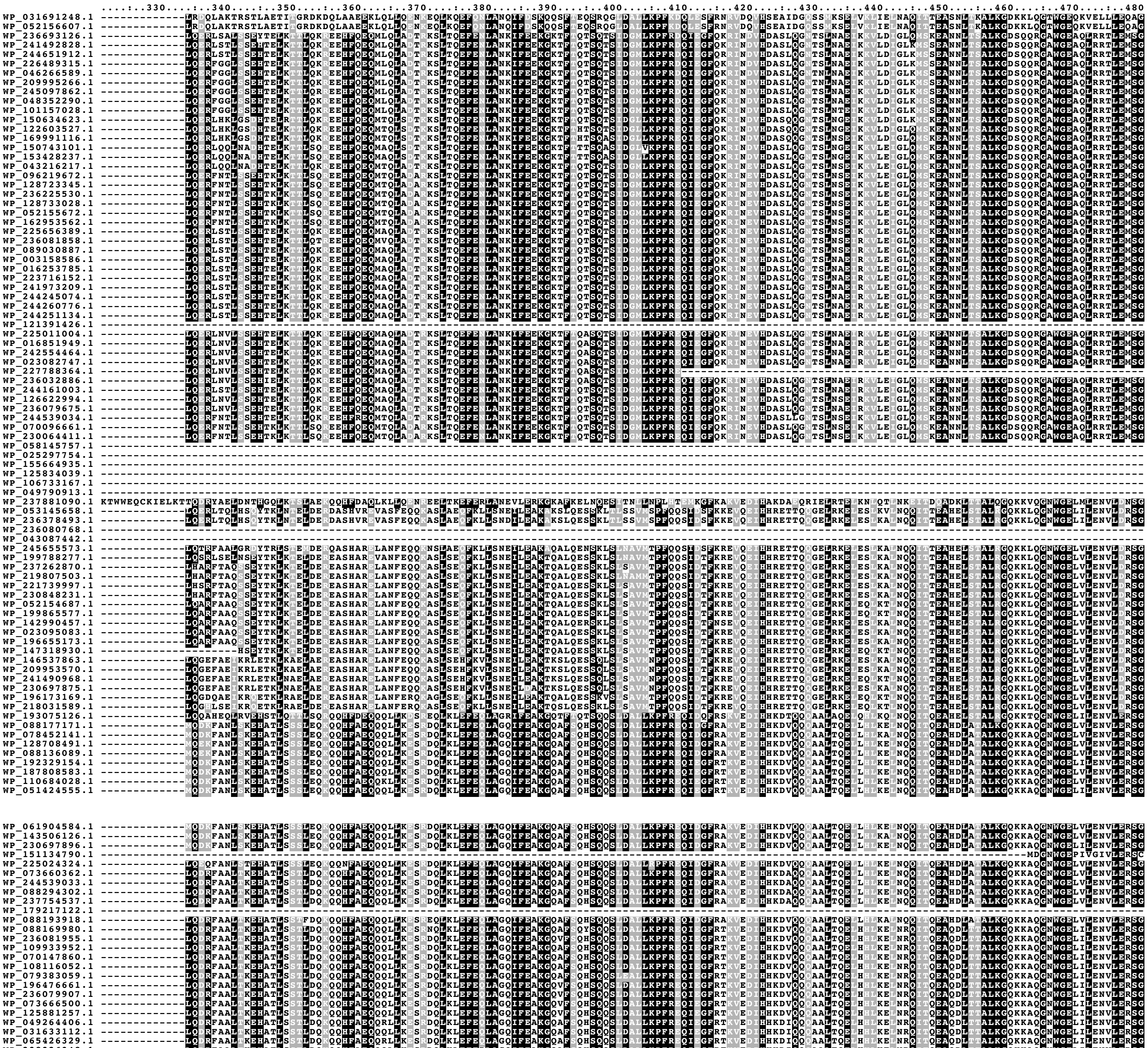
....:..330....:..340....:..350....:..360....:..370....:..380....:..390....:..400....:..410....:..420....:..430....:..440....:..450....:..460....:..470....:..480
WP_031691248.1
WP_052156607.1
WP_236693126.1
WP_241492828.1
WP_244651912.1
WP_226489315.1
WP_046266589.1
WP_209995266.1
WP_245097862.1
WP_048352290.1
WP_101157028.1
WP_150634623.1
WP_122603527.1
WP_169991116.1
WP_150743101.1
WP_153428237.1
WP_043216217.1
WP_096219672.1
WP_128723345.1
WP_236225530.1
WP_128733028.1
WP_052155672.1
WP_162953562.1
WP_225656389.1
WP_236081858.1
WP_089030887.1
WP_003158586.1
WP_016253785.1
WP_223716152.1
WP_241973209.1
WP_244245074.1
WP_244260776.1
WP_244251134.1
WP_121391426.1
WP_225011004.1
WP_016851949.1
WP_242554464.1
WP_023082747.1
WP_227788364.1
WP_236032886.1
WP_244161003.1
WP_126622994.1
WP_236079675.1
WP_244539034.1
WP_070096661.1
WP_230064411.1
WP_058145757.1
WP_025297754.1
WP_155664935.1
WP_125834039.1
WP_106733167.1
WP_049790913.1
WP_237881090.1
WP_053145658.1
WP_236378493.1
WP_236080768.1
WP_043087442.1
WP_245655573.1
WP_199788277.1
WP_237262870.1
WP_219807503.1
WP_221739997.1
WP_230848231.1
WP_052154687.1
WP_199865577.1
WP_142990457.1
WP_023095083.1
WP_196655173.1
WP_147318930.1
WP_146537863.1
WP_209953570.1
WP_241490968.1
WP_230697875.1
WP_196173169.1
WP_218031589.1
WP_193075126.1
WP_088177171.1
WP_078452141.1
WP_128708491.1
WP_088136089.1
WP_192329154.1
WP_187808583.1
WP_110684028.1
WP_051424555.1
WP_061904584.1
WP_143506126.1
WP_230697896.1
WP_151134790.1
WP_225024324.1
WP_073660362.1
WP_244539033.1
WP_088294302.1
WP_237754537.1
WP_179217122.1
WP_088193918.1
WP_088169980.1
WP_236081955.1
WP_109933952.1
WP_070147860.1
WP_108116052.1
WP_079383059.1
WP_196476661.1
WP_236079907.1
WP_073666500.1
WP_125881257.1
WP_049264406.1
WP_031633112.1
WP_065426329.1
WP_238326948.1
WP_238954630.1
WP_023115263.1
WP_241510754.1
WP_124140907.1
WP_231740201.1
WP_237752443.1
WP_233787826.1
WP_240443035.1
WP_071540622.1
WP_235581972.1
WP_140786428.1
WP_228778169.1
WP_034017985.1
WP_128704223.1
WP_172792899.1
WP_210982944.1
WP_230964886.1
WP_088135368.1
WP_078452129.1
WP_126571791.1
WP_043106458.1
WP_228761142.1
WP_063837559.1
WP_087786958.1
WP_025297452.1
WP_235584266.1
WP_198421420.1
WP_232527384.1
WP_234034845.1

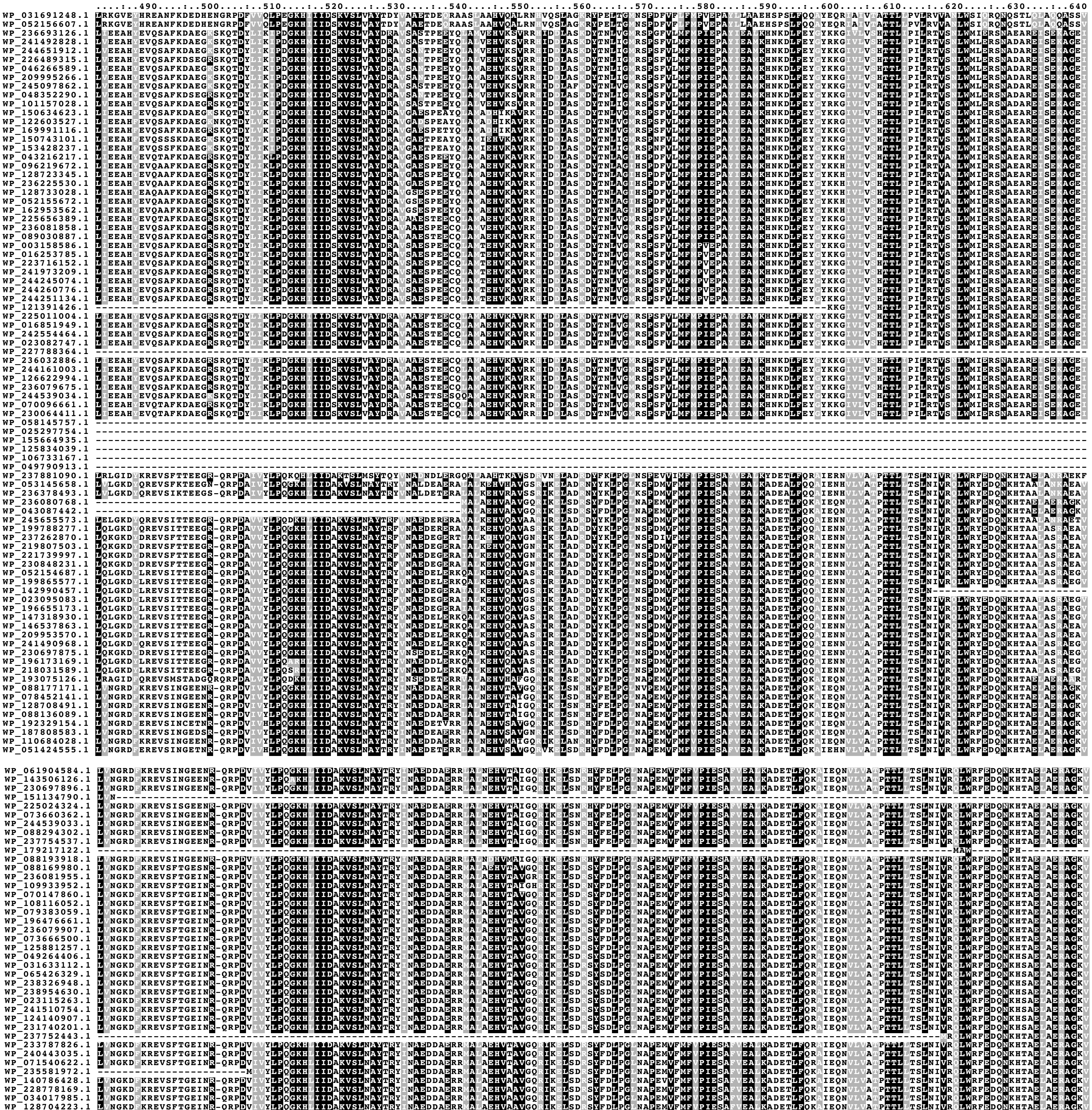

```
               ....:..650....:..660....:..670....:..680....:..690....:..700....:..710....:..720....:..730....:..740....:..750....:..
WP_031691248.1 YNKLRVFVEKMEKLGNQIGTVQKTYADTFNTLTGPHGSLTRTVDKFVDLGVKVVKRLP--------------ASVVGAGDDPAGEGDVLAMEETTE---------------------
WP_052156607.1 YNKLRVFVEKMEKLGNQIGTVQKTYADTFNTLTGPHGSLTRTVDKFVDLGVKVVKRLP--------------ASVVGAGDDPAGEGDVLAMEETTE---------------------
WP_236693126.1 YNQVCIVAERLSKLGGTLNTVSNHYNDTVKAL-AGKQGLYGKVERFNKLSAKVSKTLPQLDPTHMDFEGDRLSLIVEAIEEAPASNEPL-PAGAHDPFAGDVAAQQ-----------
WP_241492828.1 YNQVCIVAERLSKLGGSLNTASNHYNNTVKAL-AGQQGLYGKVERFDKLSAKVSKTLPQLEPVHMDFEAERLSLIFEAIEDAPASNEPL-LHSAHDPVAGEVSAQQ-----------
WP_244651912.1 YNQVCIVAERLSKLGGSLNTASNHYNGTVKAL-AGQQGLYGKVERFDKLSAKVSKTLPQLEPVHMDFEAERLSLIVEAIEETPASNEPL-LAGAHDPFAGELSAQQ-----------
WP_226489315.1 YNQVCIVAERLSKLGGSLNTASNHYNATVKAL-AGQQGLYGKVERFDKLSAKVSKTLPQLEPVHMDFEAERLSLIVEAIEETPASNGPL-LAGAHDPFAGELSAQQ-----------
WP_046266589.1 YNQVCIVAERLSKLGGSLNTASNHYNASVKAL-AGQQGLYGKVERFDKLSAKVSKTLPQLEPVHMDFEAERLSMIVEAIEETPASNGPL-LAGAHDPFAGELSAQQ-----------
WP_209995266.1 YNQVCIVAERLSKLGGSLNTASNHYNASVKAL-AGQQGLYGKVERFDKLSAKVSKTLPQLEPVHMDFEAERLSMIVEAIEETPASNGPL-LAGAHDPFAGELSAQQ-----------
WP_245097862.1 YNQVCIVAERLSKLGGSLNTASNHYNGTVKAL-AGQQGLYGKVERFDKLSAKVSKTLPQLEPVHMDFEAERLSLIVEAIEEAPASNEPL-LAGALDPFAGEFSAQQ-----------
WP_048352290.1 YNQVCIVAERLSKLGGSLNTASNHYNNTVKAL-AGQQGLYGKVERFDKLSAKVSKTLPQLEPVHMDFEAERLSLIVEAIEETPASNELL-PAGAHDPFAGELSAQQ-----------
WP_101157028.1 YNQVCIVAERLSKLGGSLNTASNHYNNTVKAL-AGQQGLYGKVERFDKLSAKVSKTLPQLEPVHVDFEVERLSLIFEAIEDTPASNEPL-LPSAHDRVAGEVSAQQ-----------
WP_150634623.1 YNQVCTVAERLGKLGGTLNTVSNHYNETVRAL-AGKQGLYGKVERFNKLSAKVSKILPALEPTHMDFESERLSLIVEAIDEAPEATEAV-QILAQQSSEEELAVQL-----------
WP_122603527.1 YNQVCTVAERLGKLGGTLNTVSNHYNETVKAL-AGKQGLYGKVERFNKLSAKVSKTLPALEPTHMDFESERLSLIVEAIDEAPEVTEAV-PLLAQQSSEEELAVQH-----------
WP_169991116.1 YNQVCTVAERLGKLGGTLNTVSNHYNETVKAL-AGKQGLYGKVERFNKLSAKVSKILPALEPTHMDFESERLSLIVEAIDEAPEATEAV-QIIAQQSSEEELAVQL-----------
WP_150743101.1 CNQVCLVAERIGKLGGTLNTASNHYNSAITAL-AGQQGLYGKIDRFSQLSAKVSKTLPALEPVHVDFQNERLSLIVEALDESAELNEIL-PQLPADEHEGEQPSLTETTPKHEFEFF
WP_153428237.1 YNQVCLVAERIGKLGGTLNTASNHYNSAITAL-AGQQGLYGKIDRFSQLSAKVSKTLPALEPVHVDFQNERLSLIVEALDESAELNEIL-PQLPADEHEGEQPSLTETTPKHEFEFF
WP_043216217.1 YNQVCTVAERIGKLGGTLNTVSNHYNDTVKAL-AGKQGLYGKVERFNKLSAKVSKTLPALEPTHMDFESERLSLIVEALDEADEPTAELPPLMNHIANDSEALPQA-----------
WP_096219672.1 YNQVCLVAERIGKLGGTLNTASNHYNSAITAL-AGQQGLYGKIDRFSQLSAKVSKTLPTLEPVHVDFQNERLSLIVEALDESPEPNESL-LQLPADDHEGEQSSHTETSPKNEFEFF
WP_128723345.1 YNQVCLVAERIGKLGGTLNTASNHYNSAITAL-AGQQGLYGKIDRFSQLSAKVSKTLPTLEPVHVDFQNERLSLIVEALDESPEPNESL-PQLPADDHEGEQSSHTETSPKNEFEFF
WP_236225530.1 YNQVCLVAERIGKLGGTLNTASNHYNSAITAL-AGQQGLYGKIDRFSQLSAKVSKTLPTLEPVHVDFQNERLSLIVEALDESPEPNESL-PQLPADDHEGEQSSHTETSPKNEFEFF
WP_128733028.1 YNQVCLVAERIGKLGGTLNTASNHYNSAITAL-AGQQGLYGKIDRFSQLSAKVSKTLPTLEPVHVDFQNERLSLIVEALDESPEPNESL-PQLPADDHEGEQSSHTETSPKNEFEFF
WP_052155672.1 YNQVCLVAERIGKLGGTLNTASNHYNSAITAL-AGQQGLYGKIDRFSQLSAKVSKTLPTLEPVHVDFQNERLSLIVEALDESPEPNESL-PQLPADDHEGEQSSHTETSLKNEFEFF
WP_162953562.1 YNQVCLVAERIGKLGGTLNTASNHYNSAITAL-AGQQGLYGK---------------------------------------------------------------------------
WP_225656389.1 YNQVCIVAERLGKLGGTLNTVSNHYNDTVKAL-AGKQGLYGKVERFNKLSAKVSKTLPALEPTHMDFESERLSLIVEALDEADESAAELPPQINHNSHDSEALPQA-----------
WP_236081858.1 YNQVCTVAERLGKLGGTLNTVSNHYNDTVKAL-AGKQGLYGKVERFNKLSAKVSKTLPALEPTHMDFESERLALIVEALDEAPDENP---LQLIQNSHEGELLPQA-----------
WP_089030887.1 YNQVCTVAERLGKLGGTLNTVSNHYNDTVKAL-AGKQGLYGKVERFNKLSAKVSKTLPALEPTHMDFESERLALIVEAIEEAPEPADDL-PQLIQNRHEGELLPQA-----------
WP_003158586.1 YNQVCTVAERLGKLGGTLNTVSNHYNDTVKAL-AGKQGLYGKVERFNKLSAKVSKTLPALEPTHMDFENERLALIVEALDEAPEPADDL-PQLIQNSHEGELLPQA-----------
WP_016253785.1 YNQVCTVAERLGKLGGTLNTVSNHYNDTVKAL-AGKQGLYGKVERFNKLSAKVSKTLPALEPTHMDFENERLALIVEALDEAPEPADDL-PQLIQNSHEGELLPQA-----------
WP_223716152.1 YNQVCTVAERLGKLGGTLNTVSNHYNDTVKAL-AGKQGLYGKVERFNKLSAKVSKTLPALEPTHMDFENERLALIVEALDEAPEPADDL-PQLIQNSHEGELLPQA-----------
WP_241973209.1 YNQVCTVAERLGKLGGTLNTVSNHYNDTVKAL-AGKQGLYGKVERFNKLSAKVSKTLPALEPTHMDFENERLALIVEALDEAPEPADDL-PQLIQNSHEGELLPQA-----------
WP_244245074.1 YNQVCTVAERLGKLGGTLNTVSNHYNDTVKAL-AGKQGLYGKVERFNKLSAKVSKTLPALEPTHMDFENERLALIVEALDEAPEPADDL-PQLIQNSHEGELLPQA-----------
WP_244260776.1 YNQVCTVAERLGKLGGTLNTVSNHYNDTVKAL-AGKQGLYGKVERFNKLSAKVSKTLPALEPTHMDFENERLALIVEALDEAPEPADDL-PQLIQNSHEGELLPQA-----------
WP_244251134.1 YNQVCTVAERLGKLGGTLNTVSNHYNDTVKAL-AGKQGLYGKVERFNKLSAKVSKTLPALEPTHMDFENERLALIVEALDEAPEPADDL-PQLIQNSHEGELLPQA-----------
WP_121391426.1 YNQVCTVAERLGKLGGTLNTVSNHYNDTVKAL-AGKQGLYGKVERFNKLSAKVSKTLPALEPTHMDFENERLALIVEALDEAPEPADDL-PQLIQNSHEGELLPQA-----------
WP_225011004.1 YNQVCTVAERLGKLGGTLNTVSNHYNDTVKAL-AGKQGLYGKVERFNKLSAKVSKTLPALEPAHMDFENERLVLIVEALDEAPEPADDL-PQLIQNSHEGELLPQA-----------
WP_016851949.1 YNQVCTVAERLGKLGGTLNTVSNHYNDTVKAL-AGKQGLYGKVERFNKLSAKVSKTLPALEPAHMDFENERLVLIVEALDEAPEPADDL-PQLIQNSHEGELLPQA-----------
WP_242554464.1 YNQVCTVAERLGKLGGTLNTVSNHYNDTVKAL-AGKQGLYGKVERFNKLSAKVSKTLPALEPAHMDFENERLVLIVEALDEAPEPADDL-PQLIQNSHEGELLPQA-----------
WP_023082747.1 YNQVCTVAERLGKLGGTLNTVSNHYNDTVKAL-AGKQGLYGKVERFNKLSAKVSKTLPALEPAHMDFENERLVLIVEALDEAPEPADDL-PQLIQNSHEGELLPQA-----------
WP_227788364.1 ---------------------------------------------------------------------------------------------------------------------
WP_236032886.1 YNQVCTVAERLGKLGGTLNTVSNHYNDTVKAL-AGKQGLYGKVERFNKLSAKVSKTLPALEPTHMDFENERLALIVEALEEASEPADDL-PQLIQNSHEGELLPQA-----------
WP_244161003.1 YNQVCTVAERLSKLGGTLNTVSNHYNDTVKAL-AGKQGLYGKVERFNKLSAKVSKTLPALEPTHMDFESERLALIVEALEEAPEPADDL-PQLIQNRHEGELLPQA-----------
WP_126622994.1 YNQVCTVAERLGKLGGTLNTVSNHYNDTVKAL-AGKQGLYGKVERFNKLSAKVSKTLPALEPTHMDFESERLALIVEALEEAPEPADDL-PQLIQNRHEGELLPQA-----------
WP_236079675.1 YNQVCTVAERLGKLGGTLNTVSNHYNDTVKAL-AGKQGLYGKVERFNKLSAKVSKTLPALEPTHMDFESERLALIVEALEEAPESADDL-PQLIQNRHEGELLPQA-----------
WP_244539034.1 YNQVCTVAERLGKLGGTLNTVSNHYNDTVKAL-AGKQGLYGKVERFNKLSAKVSKTLPALEPTHMDFESERLSLIVEALDEADEPTAELPPLMNHIANDSEALPQA-----------
WP_070096661.1 YNQVCTVAERLGKLGGTLNTVSNHYNDTVKAL-AGKQGLYGKVERFNKLSAKVSKTLPALEPTHMDFESERLSLIVEALDEADESTEELPLQINHNSHDSEALPQA-----------
WP_230064411.1 YNQVCTVAERLGKLGGTLNTVSNHYNDTVKAL-AGKQGLYGKVERFNKLSAKVSKTLPALEPTHMDFESERLSLIVEALDEADESTEELPLQINHNSHDSEALPQA-----------
WP_058145757.1 ---------------------------------------------------------------------------------------------------------------------
WP_025297754.1 ---------------------------------------------------------------------------------------------------------------------
WP_155664935.1 ---------------------------------------------------------------------------------------------------------------------
WP_125834039.1 ---------------------------------------------------------------------------------------------------------------------
WP_106733167.1 ---------------------------------------------------------------------------------------------------------------------
WP_049790913.1 ---------------------------------------------------------------------------------------------------------------------
WP_237881090.1 YNKLNGFLTSMQGVGNQLNRARESYDKAFGQLYIGKGNLIKQAAEFKDLGVSVQKELP-------VDLVERANLELSSAVAELAEIEA--TVLIASS--------------------
WP_053145658.1 FNKLNTFLGSFERIKKGLDTATEAYTKAEGQLVSGKGNLVKQVGEFKNLAPAIKAELP-------SYFADKAALEIDFIPVDPAQGAD-----CSGTESEDALIETITELEVENI--
WP_236378493.1 FNKLNTFLGSFERIKKGLDTATEAYTKAEGQLVSGKGNLVKQVGEFKNLAPAIKAELP-------SYFADKAALEIDFIPPDPLPSAD-----SNGTESEDFLTENTTELEIENI--
WP_236080768.1 YDKLRA----------AWTPSATAWTR--RRMPTRKPTISWSVARLTWSSKSVISVSWAWRLK--EVWVDRADLELNLISQMPAEQQA-----------------------------
WP_043087442.1 YDKLRA----------AWTPSATAWTR--RRMPTRKPTISWSVARLTWSSKSVISVSWAWRLK--EVWVDRADLELNLISQMPAEQQA-----------------------------
WP_245655573.1 FKKLNTFLGSFEKIKKGLETATDAYVKAEGQLVSGKGNLVKQVGEFKNLAPAIKAQLP-------AYFVDKAALEIDFI---PVVQDV--ETLNDSANDENLLAATDTDTETEHEMN
WP_199788277.1 FKKLNTFLGSFEKIKKGLETAAEAYVKAEGQLVSGKGNLVKQVGEFKNLAPAIKAQLP-------AYFVDKAALEIDFI---PTEQSI--AALTDSADDDDLLAETDTETEFEMN--
WP_237262870.1 FKKLNTFLASFEKIKKGLETATEAYVKAEGQLVSGKGNLVKQVGEFKNLAPAIKAQLP-------AYFADKAALEIDFI---PTEQNI--EVLIDSADDEELLTETDTETDYETA--
WP_219807503.1 FKKLNTFLASFEKIKKGLETATEAYVKAEGQLVSGKGNLVKQVGEFKNLAPAIKAQLP-------AYFADKAALEIDFI---PTEQSI--EVLTDSADDEELLTETDTETDYETA--
WP_221739997.1 FKKLNTFLASFEKIKKGLETATEAYVKAEGQLVSGKGNLVKQVGEFKNLAPAIKAQLP-------AYFADKAALEIDFI---PTEQSI--EVLTDSADDEELLAEADTETDYETA--
WP_230848231.1 FKKLNTFLASFERIKKGLETATEAYVKAEGQLVSGKGNLVKQVGEFKNLAPAIKAQLP-------AYFADKAALEIDFI---PTEQSI--KVLTDSADDEELLAEADTETDYETA--
WP_052154687.1 FKKLNTFLASFEKIKKGLETATEAYVKAEGQLVSGKGNLVKQVGEFKNLAPAIKAQLP-------AYFVDKAALEIDFI---PAEQHI--EVLTDSAVDEDLLDETDTETDYETA--
WP_199865577.1 FKKLNTFLASFEKIKKGLETATEAYVKAEGQLVSGKGNLVKQVGEFKNLAPAIKAQLP-------AYFVDKAALEIDFI---PAEQHI--EVLTDSAVDEDLLDETDTETDYETA--
WP_142990457.1 ---------------------------------------------------------------------------------------------------------------------
WP_023095083.1 FKKLNTFLASFEKIKKGLETATEAYVKAEGQLVSGKGNLVKQVGEFKNLAPAIKAQLP-------AYFVDKAALEIDFI---PAEQNI--EVLTDSAVDEDLLDETDTETDYETA--
WP_196655173.1 FKKLNTFLASFEKIKKGLETATEAYVKAEGQLVSGKGNLVKQVGEFKNLAPAIKAQLP-------AYFVDKAALEIDFI---PAEQNI--EVLTDSAVDEDLLDETDTETDYETA--
WP_147318930.1 FKKLNTFLASFEKIKKGLETATEAYVKAEGQLVSGKGNLVKQVGEFKNLAPAIKAQLP-------AYFVDKAALEIDFI---PAEQHI--EVLTDSAVDEDLLDETDTETDYETA--
WP_146537863.1 FKKLNTFLASFEKIKKGLENATEAYVKAEGQLVSGKGNLVKQVGEFKNLAPAIKAQLP-------AYFADKAALEIDFI---PAEQNI--TALIDSADDEDLLAETDTETDYETA--
WP_209953570.1 FKKLNTFLASFEKIKKGLENATEAYVKAEGQLVSGKGNLVKQVGEFKNLAPAIKAQLP-------AYFADKAALEIDFI---PAEQNI--TALIDSADDEDLLAETDTETDYETA--
WP_241490968.1 FKKLNTFLASFEKIKKGLETATEAYVKAEGQLVSGKGNLVKQVGEFKNLTPAIKAQLP-------AYFVDKAALEIDFI---PAEQHI--EVLTDSAVDEDLLDETDTETDYETA--
WP_230697875.1 FKKLNSFLASFEKIKKGLETATEAYVKAEGQLVSGKGNLVKQVGEFKNLAPAIKAQLP-------AYFADKAALEIDFI---PAEQNI--EALTDSADDEDLLAETDTETDYETA--
WP_196173169.1 FKKLNTFLASFEKIKKGLETATEAYVKAEGQLVSGKGNLVKQVGEFKNLAPAIKAQLP-------AYFADKAALEIDFI---PAEQNI--EALTGSTEDQDLLAESDTETDYENA--
WP_218031589.1 FKKLNTFLASFEKIKKGLENATEAYVRAEGQLVSGKGNLVKQVGEFKNLAPAIKAQLP-------AYFSDKAALEIDFI---PVEQNI--EALTDSTEDQDLLAESDTETDYENA--
WP_193075126.1 HDKLRTFLGSMDQIEKGLDRAGEAYRRARDQLINGRGNLVKQVQDFKELGVSVKGELG-------EHWTDRAELEL---GHDASADTL-----------------------------
WP_088177171.1 YDKLRTFLGSMDAIGNSLDKAQDAYRKACDQLLSGKANLVKQVSDFRQLGVAVKGELD-------ETWVDRAELELGLVEQEAFEAQEET---------------------------
WP_078452141.1 YDKLRTFLGSMDAIGNSLDKAQDAYRKARDQLVSGKANLVKQVSDFRQLGVAVKGELD-------ETWVDRAELELGLVEQEAFEVQEQA---------------------------
WP_128708491.1 YDKLRTFLGSMDAIGNSLDKAQDAYRKARDQLVSGKANLVKQVSDFRQLGVAVKGELD-------ETWVDRAELELGLVEQEAFEAQEQA---------------------------
WP_088136089.1 YDKLRTFLGSMDAIGNSLDKAQDAYRKARDQLVSGKANLVKQVSDFRQLGVAVKGELD-------ETWVDRAELELGLVEQEAFEAQEQA---------------------------
WP_192329154.1 YDKLRTFLGSMDAIGNSLDKAQDAYRKARDQLVSGKANLVKQVNDFRQLGVAVKGELA-------GEWVDRADLELALVGHEPNEILQ-----------------------------
WP_187808583.1 YDKLRTFLGSMDAIGNNLDKAQDAYRKARDQLVSGKANLVKQVSDFRQLGVAVKGELD-------ETWVDRADLELALVEHEPSELPQ-----------------------------
WP_110684028.1 YDKLRTFLGSMDAIGNSLDKAQDAYRKARDQLVSGKANLVKQVNDFRQLGVAVKGELD-------ETWVDRADLELALVEHEPSESAQ-----------------------------
WP_051424555.1 YDKLRTFLGSMDAIGNSLDKAQDAYRKARDQLVSGKANLVKQVSDFRQLGVAVKGELD-------ETWVDRADLELTLVGHKTSEMAQ-----------------------------


WP_061904584.1 YDKMRTFLGSMDAIGNSLDKAQDAYRKARDQLVSGKANLVKQVNDFRQLGVAVKGELA-------EEWVDRADLELVLVGHEPNEILQ-----------------------------
WP_143506126.1 YDKLRTFLGSMDAIGNSLDKAQDAYRKARDQLVSGKANLVKQVSDFRQLGVAVKGELD-------ETWVDRAELELGLVEQEAFEAQEEA---------------------------
WP_230697896.1 YDKLRTFLGSMDAIGNSLDKAQDAYRKARDQLVSGKANLVKQVSDFRQLGVAVKGELD-------ETWVDRADLELALVEREPSELAQ-----------------------------
WP_151134790.1 ----------------------------------GKAILVKQVSDFRQLGVSVKGELN-------EVWLDRADLELNLISQVPAEEQA-----------------------------
WP_225024324.1 YDKLRTFLGSMDAIGNSLDKAQDAYRKARDQLVSGKGNLVKQVSDFRQLGVAVKGELD-------ESWVDRADLELSLIEQGSLETQE-----------------------------
WP_073660362.1 YDKLRTFLGSMDAIGNSLDKAQDAYRKARDQLVSGKANLVKQVNDFRQLGVAVKGELA-------EEWVDRAELELTLIERESSEMAQ-----------------------------
WP_244539033.1 YDKLRTFLGSMDAIGNSLDKAQDAYRKARDQLVSGKANLVKQVNDFRQLGVAVKGELA-------EEWVDRAELELTLIERESSEMAQ-----------------------------
WP_088294302.1 YDKLRTFLGSMDAIGNSLDKAQDAYRKARDQLVSGKANLVKQVNDFRQLGVAVKGELA-------EEWVDRAELELTLIERESSEMAQ-----------------------------
WP_237754537.1 YDKLRTFLGSMDAIGNSLDKAQDAYRKARDQLVSGKANLVKQVNDFRQLGVAVKGELA-------EEWVDRAELELTLIERESSEMAQ-----------------------------
WP_179217122.1 ---------------HSLDKAQDAYKKACDQLVSGKANLVKQVSDFRQLGVAVKGELN-------EVWVDRADLELNLISQMPAEQQA-----------------------------
WP_088193918.1 YDKLRTFLGSMDVIGHSLDKAQDAYKKACDQLVNGKANLVKQVSDFRQLGVAVKGELA-------EEWVERADLELALAGHEPNEILQ-----------------------------
WP_088169980.1 YDKLRTFLGSMDAIGHS----------------------CGFHADSDTHSTHIRTVIPR----------------------------------------------------------
WP_236081955.1 YDKLRTFLSSMDAIGHSLDKAQDAYKKACDQLVSGKANLVKQVSDFRQLGVAVKGELA-------EEWVERADLELALAGHEPNETLQ-----------------------------
WP_109933952.1 YDKLRTFLGSMDAIGHSLDKAQDAYKKACDQLVNGKANLVKQVSDFRQLGVAVKGELN-------EVWVDRADLELNLISQMPAEQQA-----------------------------
WP_070147860.1 YDKLRTFLGSMDAIGHSLDKAQDAYKKACDQLVNGKANLVKQVSDFRQLGVAVKGELS-------EVWGDRADLELNLIPQMPAEQSM-----------------------------
WP_108116052.1 YDKLRTFLGSMDAIGHSLDKAQDAYKKACDQLVNGKANLVKQVSDFRQLGVAVKGELS-------EVWGDRADLELNLIPQMPAEQSM-----------------------------
WP_079383059.1 YDKLRTFLSSMDAIGHSLDRAQDAYKKACDQLVSGKANLVKQVSDFRQLGVAVKGELN-------EVWVDRADLELNLISQMPAEQQA-----------------------------
WP_196476661.1 YDKLRTFLSSMDAIGHSLDRAQDAYKKACDQLVSGKANLVKQVSDFRQLGVAVKGELN-------EVWVDRADLELNLISQMPAEQQA-----------------------------
WP_236079907.1 YDKLRTFLSSMDAIGHSLDRAQDAYKKACDQLVSGKANLVKQVSDFRQLGVAVKGELN-------EVWVDRADLELNLISQMPAEQQA-----------------------------
WP_073666500.1 YDKLRTFLSSMDAIGHSLDRAQDAYKKACDQLVSGKANLVKQVSDFRQLGVAVKGELN-------EVWVDRADLELNLISQMPAEQQA-----------------------------
WP_125881257.1 YDKLRTFLSSMDAIGHSLDRAQDAYKKACDQLVSGKANLVKQVSDFRQLGVAVKGELN-------EVWVDRADLELNLISQMPAEQQA-----------------------------
WP_049264406.1 YDKLRTFLGSMDAIGHSLDKAQDAYKKACDQLVSGKANLVKQVSDFRQLGVAVKGELN-------EVWVDRADLELNLISQMPAEQQA-----------------------------
WP_031633112.1 YDKLRTFLGSMDAIGHSLDKAQDAYKKACDQLVSGKANLVKQVSDFRQLGVAVKGELN-------EVWVDRADLELNLISQMPAEQQA-----------------------------
WP_065426329.1 YDKLRTFLGSMDAIGHSLDKAQDAYKKACDQLVSGKANLVKQVSDFRQLGVAVKGELN-------EVWVDRADLELNLISQMPAEQQA-----------------------------
WP_238326948.1 YDKLRTFLGSMDAIGHSLDKAQDAYKKACDQLVSGKANLVKQVSDFRQLGVAVKGELN-------EVWVDRADLELNLISQMPAEQQA-----------------------------
WP_238954630.1 YDKLRTFLGSMDAIGHSLDKAQDAYKKACDQLVSGKANLVKQVSDFRQLGVAVKGELN-------EVWVDRADLELNLISQMPAEQQA-----------------------------
WP_023115263.1 YDKLRTFLGSMDAIGHSLDKAQDAYKKACDQLVSGKANLVKQVSDFRQLGVAVKGELN-------EVWVDRADLELNLISQMPAEQQA-----------------------------
WP_241510754.1 YDKLRTFLGSMDAIGRSLDKAQDAYKKACDQLVSGKANLVKQVSDFRQLGVAVKGELN-------EVWVDRADLELNLISQMPAEQQA-----------------------------
WP_124140907.1 YDKLRTFLGSMDAIGHSLDKAQDAYKKACDQLVSGKANLVKQVSDFRQLGVAVKGELN-------EVWVDRADLELNLISQMPAEQQA-----------------------------
WP_231740201.1 YDKLRTFLGSMDAIGHSLDKAQDAYKKACDQLVSGKANLVKQVSDFRQLGVAVKGELN-------EVWVDRADLELNLISQMPAEQQA-----------------------------
WP_237752443.1 YDKLRTFLGSMDAIGHSLDKAQDAYKKACDQLVSGKANLVKQVSDFRQLGVAVKGELN-------EVWVDRADLELNLISQMPAEQQA-----------------------------
WP_233787826.1 YDKLRTFLGSMDAIGHSLDKAQDAYKKACDQLVSGKANLVKQVSDFRQLGVAVKGELS-------EVWVDRADLELNLISQMPAEQQA-----------------------------
WP_240443035.1 YDKLRTFLGSMDAIGHSLDKAQDAYKKACDQLVSGKANLVKQVSDFRQLGVAVKGELN-------EVWVDRADLELNLISQMPAEQQA-----------------------------
WP_071540622.1 YDKLRTFLGSMDAIGHSLDKAQDAYKKACDQLVSGKANLVKQVSDFRQLGVAVKGELN-------EVWVDRADLELNLISQMPAEQQA-----------------------------
WP_235581972.1 YDKLRTFLGSMDAIGHSLDKAQDAYKKACDQLVSGKANLVKQVSDFRQLGVAVKGELN-------EVWVDRADLELNLISQMPAEQQA-----------------------------
WP_140786428.1 YDKLRTFLGSMDAIGHSLDKAQDAYKKACDQLVSGKANLVKQVSDFRQLGVAVKGELN-------EVWVDRADLELNLISQMPAEQQA-----------------------------
WP_228778169.1 YDKLRTFLGSMDAIGHSLDKAQDAYKKACDQLVSGKANLVKQVSDFRQLGVAVKGELN-------EVWVDRADLELNLISQMPAEQQA-----------------------------
WP_034017985.1 YDKLRTFLGSMDAIGHSLDKAQDAYKKACDQLVSGKANLVKQVSDFRQLGVAVKGELN-------EVWVDRADLELNLISQMPAEQQA-----------------------------
WP_128704223.1 YDKLRTFLGSMDAIGHSLDKAQDAYKKACDQLVSGKANLVKQVSDFRQLGVAVKGELN-------EVWVDRADLELNLISQMPAEQQA-----------------------------
WP_172792899.1 YDKLRTFLGSMDAIGHSLDKAQDAYKKACDQLVSGKANLVKQVSDFRQLGVAVKGELN-------EVWVDRADLELNLISQMPAEQQA-----------------------------
WP_210982944.1 YDKLRTFLGSMDAIGHSLDKAQDAYKKACDQLVSGKANLVKQVSDFRQLGVAVKGELN-------EVWVDRADLELNLISQMPAEQQA-----------------------------
WP_230964886.1 YDKLRTFLGSMDAIGHSLDKAQDAYKKACDQLVSGKANLVKQVSDFRQLGVAVKGELN-------EVWVDRADLELNLISQMPAEQQA-----------------------------
WP_088135368.1 YDKLRTFLGSMDAIGHSLDKAQDAYKKACDQLVSGKANLVKQVSDFRQLGVAVKGELN-------EVWVDRADLELNLISQMPAEQQA-----------------------------
WP_078452129.1 YDKLRTFLGSMDAIGHSLDKAQDAYKKACDQLVSGKANLVKQVSDFRQLGVAVKGELN-------EVWVDRADLELNLISQMPAEQQA-----------------------------
WP_126571791.1 YDKLRTFLGSMDAIGHSLDKAQDAYKKACDQLVSGKANLVKQVSDFRQLGVAVKGELN-------EVWVDRADLELNLISQMPAEQQA-----------------------------
WP_043106458.1 YDKLRTFLGSMDAIGHSLDKAQDAYKKACDQLVSGKANLVKQVSDFRQLGVAVKGELN-------EVWVDRADLELNLISQMPAEQQA-----------------------------
WP_228761142.1 YDKLRTFLGSMDAIGHSLDKAQDAYKKACDQLVSGKANLVKQVSDFRQLGVAVKGELN-------EVWVDRADLELNLISQMPAEQQA-----------------------------
WP_063837559.1 YDKLRTFLGSMDAIGHSLDKAQDAYKKACDQLVSGKANLVKQVSDFRQLGVAVKGELN-------EVWVDRADLELNLISQMPAEQQA-----------------------------
WP_087786958.1 YDKLRTFLGSMDAIGHSLDKAQDAYKKACDQLVSGKANLVKQVSDFRQLGVAVKGELN-------EVWVDRADLELNLISQMPAEQQA-----------------------------
WP_025297452.1 YDKLRTFLGSMDAIGHSLDKAQDAYKKACDQLVSGKANLVKQVSDFRQLGVAVKGELN-------EVWVDRADLELNLISQMPAEQQA-----------------------------
WP_235584266.1 YDKLRTFLGSMDAIGHSLDKAQDAYKKACDQLVSGKANLVKQVSDFRQLGVAVKGELN-------EVWVDRADLELNLISQMPAEQQA-----------------------------
WP_198421420.1 YDKLRTFLGSMDAIGHSLDKAQDAYKKACDQLVSGKANLVKQVSDFRQLGVAVKGELN-------EVWVDRADLELNLISQMPAEQQA-----------------------------
WP_232527384.1 YDKLRTFLGSMDAIGHSLDKAQDAYKKACDQLVSGKANLVKQVSDFRQLGVAVKGELN-------EVWVDRADLELNLISQMPAEQQA-----------------------------
WP_234034845.1 YDKLRTFLGSMDAIGHSLDKAQDAYKKACDQLVSGKANLVKQVSDFRQLGVAVKGELN-------EVWVDRADLELNLISQMPAEQQA-----------------------------
```

Figure S6: Alignment of ShdA homologues from different Shield subtypes and DISARM-associated ShdA. Alignment of ShdA II homologues using MUSCLE. The alignment was coloured in Boxshade by percentage of identity. Sequence conservation between Shield- and DISARM-associated ShdA homologues is still present but is reduced to what observed in Figure S2.
