# Supplementary material for: The novel anti-phage system Shield co-opts an RmuC domain to mediate phage defense across Pseudomonas species
Source: PLoS Genet. 2023 Jun 5;19(6):e1010784. doi: 10.1371/journal.pgen.1010784 (PMC10270631; doi:10.1371/journal.pgen.1010784)
Supplement: S7 Fig — (a) One-step growth curve for E. coli MG1655 carrying pBAD 18 (VC,) or the same plasmid encoding Shield II, ShdA II only or ShdB II only when infected with φSipho. Strains were grown in LB supplemented with 0.2% L- arabinose and infected at time zero with MOI = 0.1. PFU/mL were evaluated at t = 5 min, t = 10 min, t = 15 min, t = 20 min, t = 30 min, t = 45 min, t = 60 min, t = 75 min, t = 90 min, t = 105 min and t = 120 min. (b) Growth curves of E. coli MG1655 carrying pBAD 18 (VC,) or the same plasmid encoding Shield II, ShdA II only or ShdB II only. Strains were grown in LB supplemented with 0.2% L-arabinose and infected at time zero with MOI = 1, MOI = 0.1, MOI = 0.01 of φSipho and φTB34. (c) Evaluation of efficiency of centre of infection (ECOI) for E. coli MG1655 carrying pBAD 18 (VC) or the same plasmid encoding Shield II, ShdA II only or ShdB II only when challenged with phage φSipho at MOI = 0.1. grown in LB supplemented with 0.2% L-arabinose and assays were performed as described in Material and Methods. For all panels, points show mean +/- SEM (n = 3 biological replicates). Statistical relevance was measured using one-way ANOVA with Dunnett’s multiple comparison test. No significance was detected unless indicated (*p≤0.05). (PDF) [file pgen.1010784.s019.pdf]

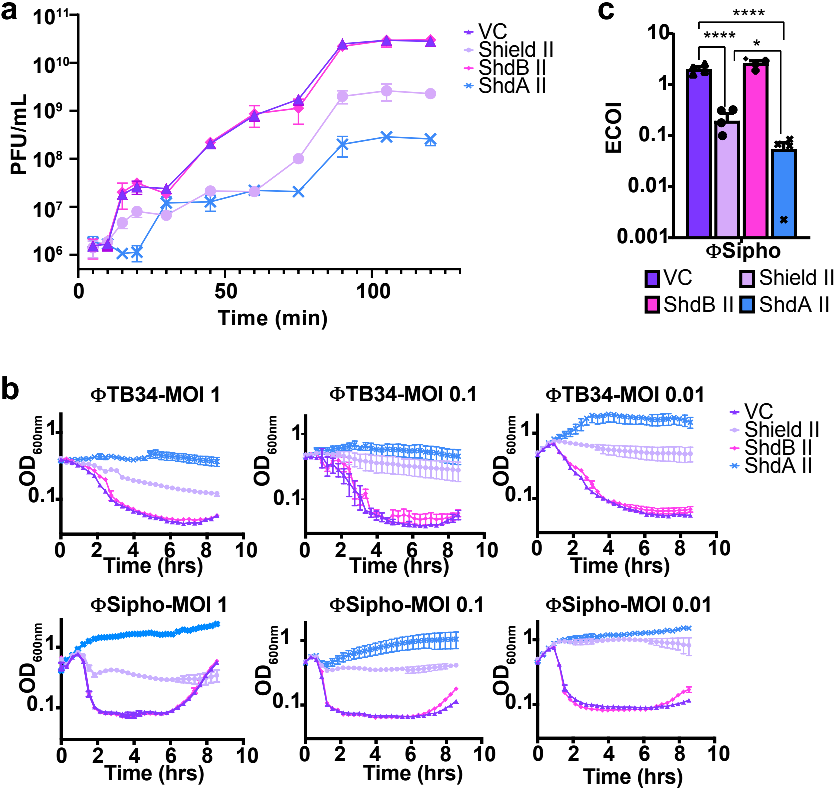

a
PFU/mL
Time (min)
VC
Shield II
ShdB II
ShdA II
b
ΦTB34-MOI 1
ΦTB34-MOI 0.1
ΦTB34-MOI 0.01
ΦSipho-MOI 1
ΦSipho-MOI 0.1
ΦSipho-MOI 0.01
OD600nm
Time (hrs)
c
ECOI
ΦSipho
****
****
*

Figure S7: ShdA II reduces the phage burden. (a) One-step growth curve for *E. coli* MG1655 carrying pBAD 18 (VC,) or the same plasmid encoding Shield II, ShdA II only or ShdB II only when infected with ϕSipho. Strains were grown in LB supplemented with 0.2 % L-arabinose and infected at time zero with MOI = 0.1. PFU/mL were evaluated at t = 5 min, t = 10 min, t = 15 min, t = 20 min, t = 30 min, t = 45 min, t = 60 min, t = 75 min, t = 90 min, t = 105 min and t = 120 min. **(b)** Growth curves of *E. coli* MG1655 carrying pBAD 18 (VC,) or the same plasmid encoding Shield II, ShdA II only or ShdB II only. Strains were grown in LB supplemented with 0.2 % L-arabinose and infected at time zero with MOI = 1, MOI = 0.1, MOI = 0.01 of ϕSipho and ϕTB34. **(c)**Evaluation of efficiency of centre of infection (ECOI) for E. coli MG1655 carrying pBAD 18 (VC) or the same plasmid encoding Shield II, ShdA II only or ShdB II only when challenged with phage ϕSipho at MOI = 0.1. grown in LB supplemented with 0.2% L-arabinose and assays were performed as described in Material and Methods. For all panels, points show mean +/- SEM (n = 3 biological replicates). Statistical relevance was measured using one-way ANOVA with Dunnett’s multiple comparison test. No significance was detected unless indicated (*$p \leq 0.05$).
