# Supplementary material for: The novel anti-phage system Shield co-opts an RmuC domain to mediate phage defense across Pseudomonas species
Source: PLoS Genet. 2023 Jun 5;19(6):e1010784. doi: 10.1371/journal.pgen.1010784 (PMC10270631; doi:10.1371/journal.pgen.1010784)
Supplement: S8 Fig — The same region of interest (ROI) used for quantification of the DAPI fluorescence intensity at the cell periphery shown in Fig 6B were used to quantify the a) the variance of pixel intensity and b) the integrated pixel intensity of the cellular DAPI signals for the t = 2 hrs timepoint (see Material and Methods for details). Statistical analysis was performed using one-way ANOVA with Dunnett’s multiple comparison test. No significance was detected, unless indicated (*p ≤ 0.05). (c-f) To test if the variance of pixel intensity observed in cell expressing ShdA or Shield is influenced by the concurrent increased in DAPI staining, the correlation was analysed. The graphs depict the cell-to-cell correlation of fluorescence variance and integrated intensity, together with a linear regression and its R2 values. Only a very low correlation was observed with between DAPI intensity and its variance throughout the tested strains, thus ruling out that the increased variance observed ShdA or Shield -expressing cells is due to higher overall DAPI staining levels. (PDF) [file pgen.1010784.s020.pdf]

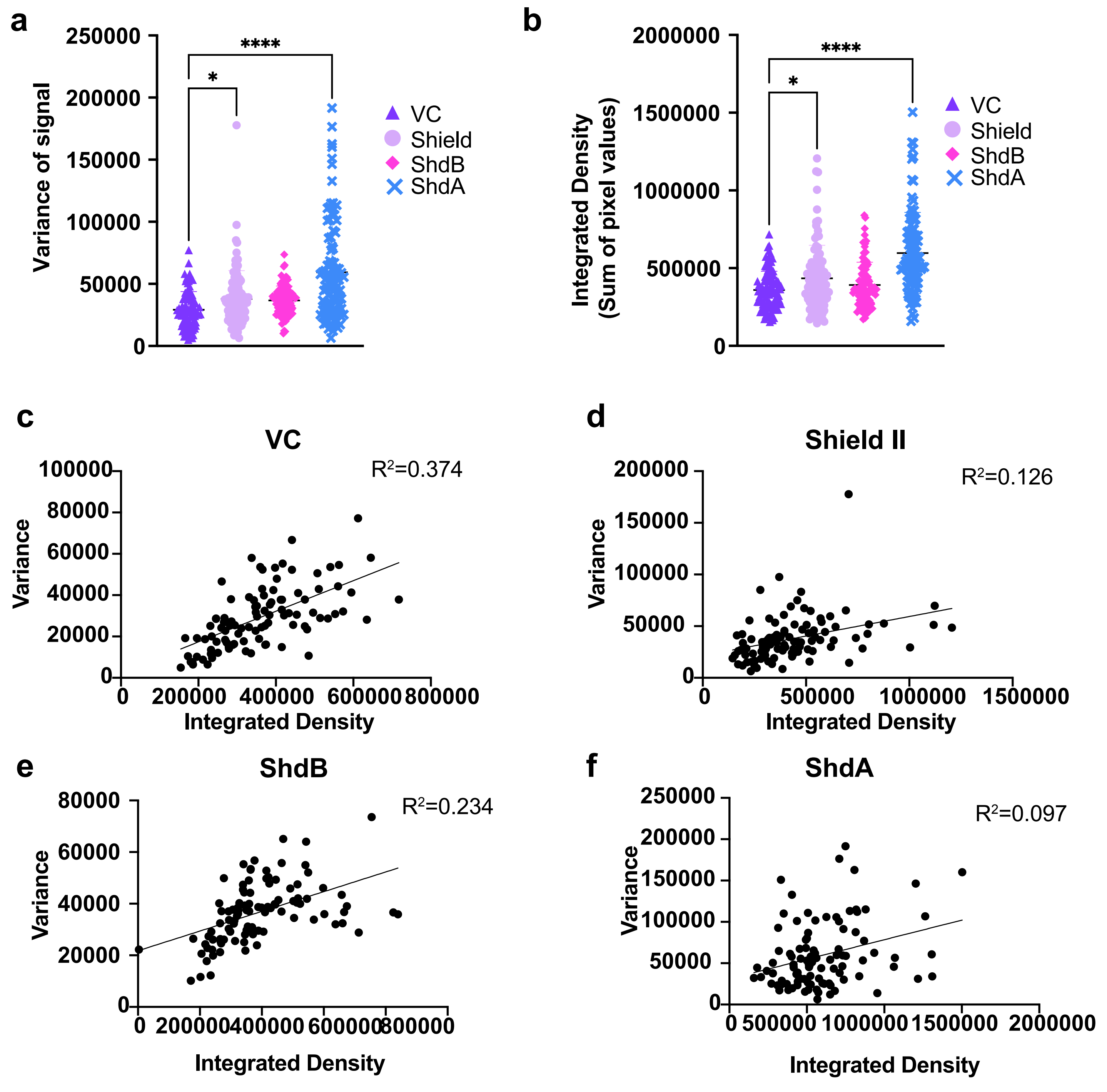

a
Variance of signal
250000
200000
150000
100000
50000
0
*
****
VC
Shield
ShdB
ShdA
b
Integrated Density (Sum of pixel values)
2000000
1500000
1000000
500000
0
*
****
VC
Shield
ShdB
ShdA
c
VC
R²=0.374
Variance
Integrated Density
d
Shield II
R²=0.126
Variance
Integrated Density
e
ShdB
R²=0.234
Variance
Integrated Density
f
ShdA
R²=0.097
Variance
Integrated Density

Figure S8: ShdA II mediates chromosome re-arrangements but does not lead to degradation *in vivo*. The same region of interest (ROI) used for quantification of the DAPI fluorescence intensity at the cell periphery shown in Figure 6b were used to quantify the **a)** the variance of pixel intensity and **b)** the integrated pixel intensity of the cellular DAPI signals for the t=2 hrs timepoint (see Material and Methods for details). Statistical analysis was performed using one-way ANOVA with Dunnett's multiple comparison test. No significance was detected, unless indicated (*$p \leq 0.05$). **(c-f)** To test if the variance of pixel intensity observed in cell expressing ShdA or Shield is influenced by the concurrent increased in DAPI staining, the correlation was analysed. The graphs depict the cell-to-cell correlation of fluorescence variance and integrated intensity, together with a linear regression and its R^2 values. Only a very low correlation was observed with between DAPI intensity and its variance throughout the tested strains, thus ruling out that the increased variance observed ShdA or Shield -expressing cells is due to higher overall DAPI staining levels.
