# Supplementary material for: The novel anti-phage system Shield co-opts an RmuC domain to mediate phage defense across Pseudomonas species
Source: PLoS Genet. 2023 Jun 5;19(6):e1010784. doi: 10.1371/journal.pgen.1010784 (PMC10270631; doi:10.1371/journal.pgen.1010784)
Supplement: S9 Fig — (a) Alphafold predicted structure of ShdB II was used for Dali predictions (S4 Table), showing it harbours a predicted peptidase M15 domain. (b) Local Distance Difference Test (lDDT) relative to ShdB II predicted structure. IDDT shows a per-residue measure of local confidence for the prediction and it is high across the whole ShdB II structure. (c) Predicted Aligned Error (PAE) for ShdB II Alphafold-predicted structure. PAE reports the expected error at each residue position. For ShdB II structure PAE was low across the whole sequence. (PDF) [file pgen.1010784.s021.pdf]

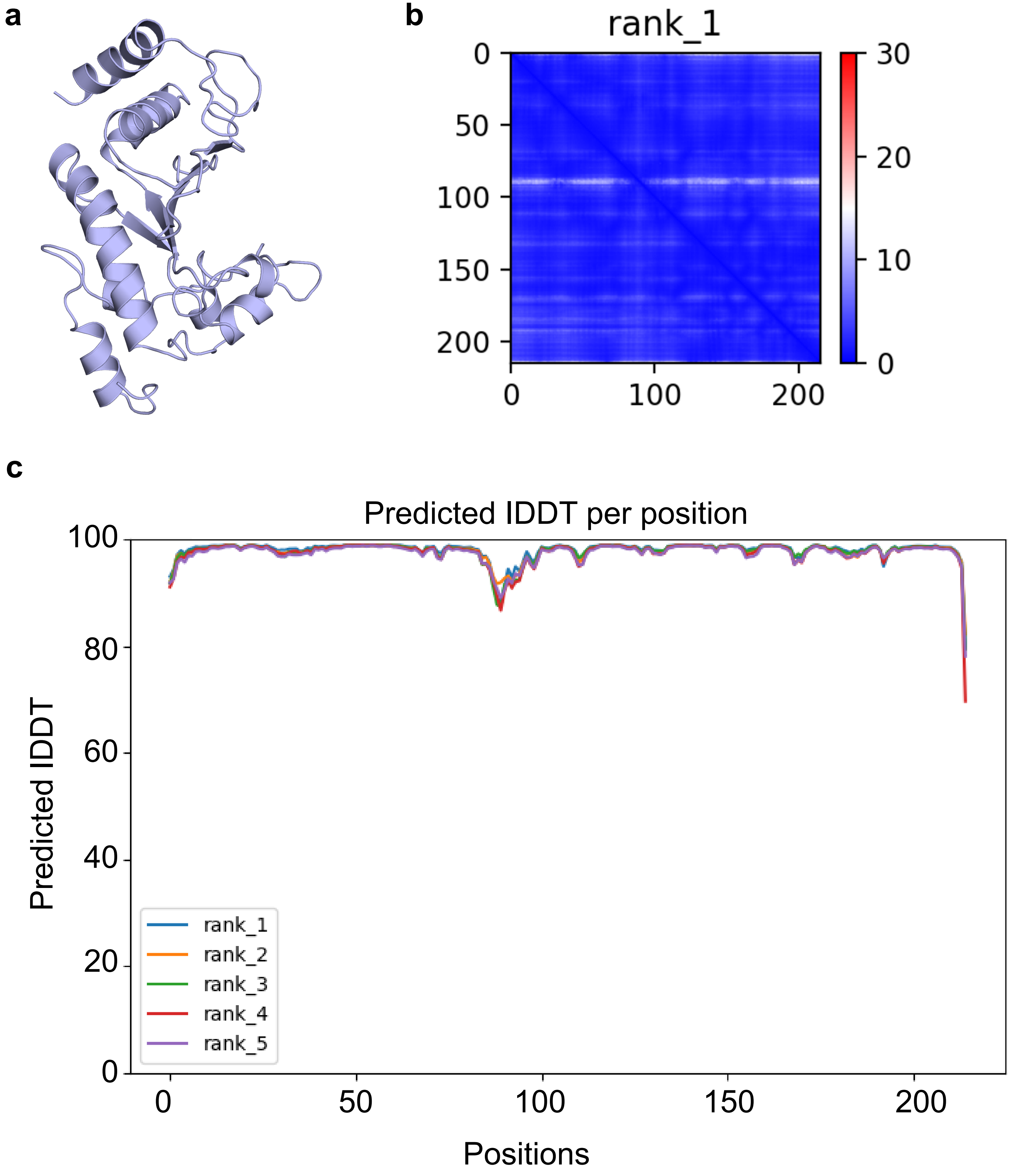

a
b
rank_1
0
50
100
150
200
0
100
200
30
20
10
0
c
Predicted IDDT per position
Predicted IDDT
100
80
60
40
20
0
rank_1
rank_2
rank_3
rank_4
rank_5
0
50
100
150
200
Positions

**Figure S9: ShdB II structural prediction suggests a peptidase M15 fold*.* (a)** Alphafold predicted structure of ShdB II was used for Dali predictions (Table S4), showing it harbours a predicted peptidase M15 domain. **(b)** Local Distance Difference Test (lDDT) relative to ShdB II predicted structure. lDDT shows a per-residue measure of local confidence for the prediction and it is high across the whole ShdB II structure. **(c)** Predicted Aligned Error (PAE) for ShdB II Alphafold-predicted structure. PAE reports the expected error at each residue position. For ShdB II structure PAE was low across the whole sequence.
