# Supplementary material for: The novel anti-phage system Shield co-opts an RmuC domain to mediate phage defense across Pseudomonas species
Source: PLoS Genet. 2023 Jun 5;19(6):e1010784. doi: 10.1371/journal.pgen.1010784 (PMC10270631; doi:10.1371/journal.pgen.1010784)
Supplement: S10 Fig — (a) ShdB II expression with a signal peptide does not impair cell growth. Growth in liquid LB media of E. coli MG1655 carrying (VC, pBAD18), ShdB II or ShdB II with an OmpA signal sequence (sp-ShdB II). Expression was repressed with 0.2% D-glucose or induced with addition of 0.2% L-arabinose. Points show mean +/− SEM (n = 3 biological replicates). (b) Analysis of E. coli BTH101 carrying combinations of ShdA II and ShdB II, when cloned in bacterial two-hybrid vectors pUT18 or pT25 vectors as indicated. Cloning in pUT18 and pT25 allows fusion of candidate proteins to the UT18 and T25 fragments of the adenylate cyclase. Upon interaction of candidate proteins, the adenylate cyclase is reconstituted, producing the cAMP signal, in turn activating the maltose catabolism operon, resulting in red colonies on MacConkey medium. The empty pUT18 or pT25 vectors were used as negative controls, while the interaction between NarG and NarJ proteins was employed as a positive control (58). (c) ShdA II-His6 or ShdA II-His6 + ShdB II were expressed from an arabinose-inducible plasmid pBAD18. and ShdA II-His6 levels were assessed by western blot analysis (See Material and Methods). GroEL was used as loading control. (PDF) [file pgen.1010784.s022.pdf]

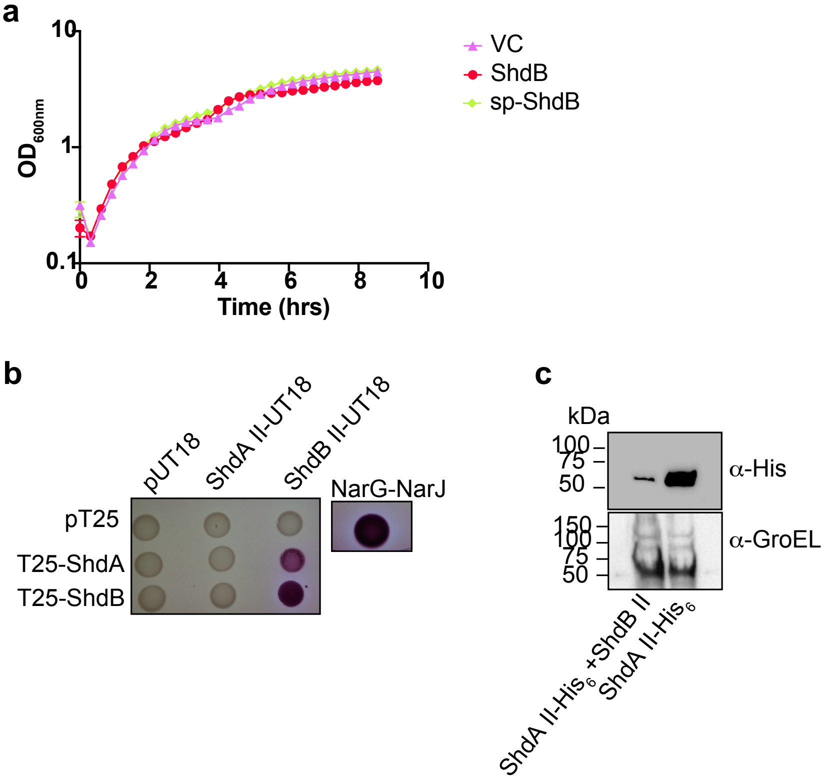
a
10
1
0.1
OD_{600nm}
0
2
4
6
8
10
Time (hrs)
VC
ShdB
sp-ShdB
b
pUT18
ShdA II-UT18
ShdB II-UT18
NarG-NarJ
pT25
T25-ShdA
T25-ShdB
c
kDa
100
75
50
α-His
150
100
75
50
α-GroEL
ShdA II-His6 +ShdB II
ShdA II-His6

Figure S10: ShdB is a probable peptidase that negatively affects the cellular level of ShdA (a) ShdB II expression with a signal peptide does not impair cell growth**.** Growth in liquid LB media of E. coli MG1655 carrying (VC, pBAD18), ShdB II or ShdB II with an OmpA signal sequence (sp-ShdB II). Expression was repressed with 0.2% D-glucose or induced with addition of 0.2% L-arabinose. Points show mean +/− SEM (n = 3 biological replicates). **(b)** Analysis of *E. coli* BTH101 carrying combinations of ShdA II and ShdB II, when cloned in bacterial two-hybrid vectors pUT18 or pT25 vectors as indicated. Cloning in pUT18 and pT25 allows fusion of candidate proteins to the UT18 and T25 fragments of the adenylate cyclase. Upon interaction of candidate proteins, the adenylate cyclase is reconstituted, producing the cAMP signal, in turn activating the maltose catabolism operon, resulting in red colonies on MacConkey medium. The empty pUT18 or pT25 vectors were used as negative controls, while the interaction between NarG and NarJ proteins was employed as a positive control (58). **(c)** ShdA II-His_6 or ShdA II-His_6 + ShdB II were expressed from an arabinose-inducible plasmid pBAD18. and ShdA II-His_6 levels were assessed by western blot analysis (See Material and Methods). GroEL was used as loading control
